# Development of the first geldanamycin-based HSP90 degraders

**DOI:** 10.3389/fchem.2023.1219883

**Published:** 2023-06-28

**Authors:** Silas Wurnig, Melina Vogt, Julian Hogenkamp, Niklas Dienstbier, Arndt Borkhardt, Sanil Bhatia, Finn K. Hansen

**Affiliations:** ^1^ Department of Pharmaceutical and Cell Biological Chemistry, Pharmaceutical Institute, University of Bonn, Bonn, Germany; ^2^ Department of Pediatric Oncology, Hematology and Clinical Immunology, Medical Faculty, Heinrich Heine University Düsseldorf, Düsseldorf, Germany

**Keywords:** cancer, cereblon, heat shock protein, Hsp90, PROTAC

## Abstract

Despite the early clinical promise, adverse events such as acquired resistance and dose-limiting toxicities have barred the widespread use of HSP90 inhibitors as anticancer drugs. A new approach involving proteolysis-targeting chimeras (PROTACs) to degrade the protein instead of inhibiting it may overcome these problems. In this work, we describe the design, synthesis, and evaluation of cereblon-recruiting geldanamycin-based HSP90 degraders based on the PROTAC technology. Our best degrader, **3a**, effectively decreased HSP90α and HSP90β levels in cells utilizing the ubiquitin–proteasome pathway.

## 1 Introduction

When cells encounter external stressors, such as radiation, heat, hypoxia, or infections, their vital processes need to remain unaffected. This crucial role in the cells is performed by a group of chaperone proteins known as heat shock proteins (HSPs) (Hoter et al., 2018). Cancer cells hijack these pathways to promote their survival and growth. Among the chaperone proteins, HSP of 90 kDa (HSP90) has been extensively studied, maintaining protein homeostasis by assisting in protein folding, stabilization, and degradation (Jackson, 2013; Schopf et al., 2017; Hoter et al., 2018; Biebl and Buchner, 2019). Client proteins of HSP90 fulfill essential roles in signaling cascades, including cell cycle, cell proliferation, differentiation, and apoptosis (Miyata et al., 2013). In mammalian cells, two dominant homologs are found in the cytoplasm: HSP90α, the stress-inducible isoform, and HSP90β, the constitutively expressed isoform (Jackson, 2013). Elevated levels of HSP90 have been found in a variety of cancer types, suggesting that HSP90 plays a central role in malignant cell survival and growth (Csermely et al., 1998; Mayer and Bukau, 1999; Bhatia et al., 2018; Park et al., 2020). For instance, enriched HSP90 expression in the therapy-resistant BCR-ABL1^+^ leukemia subgroup facilitates proper folding of BCR-ABL1 oncoprotein, providing a rationale to target leukemia cells via HSP90 inhibition-mediated BCR-ABL1 oncoprotein degradation (Bhatia et al., 2018). Therefore, the inhibition of HSP90 proteins represents a viable option for the development of new anticancer drugs (Birbo et al., 2021).

The different HSP90 isoforms (HSP90α, HSP90β, GRP94, and TRAP1) share a high sequence identity with respect to their *N*-terminal ATP binding pocket. This pocket is targeted by most inhibitors published to date (Park et al., 2020). Until now, over 15 HSP90 inhibitors with different pharmacological properties have entered clinical trials (Jhaveri et al., 2014; Koren and Blagg, 2020). However, most of these inhibitors displayed poor risk–benefit profiles in the treatment of cancer. This is mostly attributed to dose-dependent hepatotoxicity, ocular toxicity, and the development of chemoresistance (Butler et al., 2015). Notably, the inhibition of HSP90 leads to a dose-dependent depletion of the client proteins, resulting in a heat shock response (HSR) induced by the heat shock factor 1 (HSF1). HSF1 is a transcription factor that, when activated, induces the production of HSP27, HSP40, and HSP70, which in turn protects the cells from apoptosis and cytotoxic stress (Park et al., 2020).

The protein can also be degraded using proteolysis-targeting chimeras (PROTACs) to circumvent problems occurring with traditional protein inhibition, such as overexpression of the target. This approach utilizes a chimeric molecule with an E3 ligase ligand and a warhead that specifically targets the desired protein of interest (POI) connected by a suitable linker moiety (Sosic et al., 2022). Due to the formation of an E3 ligase:PROTAC:POI ternary complex, the POI is recruited into the proximity of the E3 ligase system, leading to POI polyubiquitination and subsequent degradation via the proteasomal system (Sosic et al., 2022).

Geldanamycin (GM), which was isolated from the bacterium *Streptomyces hygroscopicus*, was originally identified as an Src kinase inhibitor (Miller et al., 1994). Further research demonstrated that geldanamycin is a potent inhibitor of HSP90. The ansamycin derivative containing a benzoquinone moiety binds to the *N*-terminal ATP binding pocket of HSP90 proteins (Deboer et al., 1970). Despite the high potency of GM, it has not progressed into the clinic due to its poor physicochemical properties and unacceptable adverse effects, such as high hepatotoxicity and ocular toxicity (Li et al., 2019). Thus, a series of more soluble and stable semi-synthetic GM analogs were developed. These compounds are characterized by a substitution of the methoxy group in position 17 by an amine-based sidechain such as 17-allylamino (17-AAG, tanespimycin) or 17-[2-(dimethylamino)ethyl]amino (17-DMAG, alvespimycin) (Li et al., 2019; Talaei et al., 2019). However, this also did not lead to success at the clinical level, particularly due to HSR induction and toxicity-related concerns (Butler et al., 2015). Consequently, the targeted degradation of HSP90 via PROTACs might provide an alternative approach to treating HSP90-driven diseases. Thus far, only one report on HSP90 degraders was published by Liu et al. (2022), in which the clinical candidate and purine-derivative BIIB021 was utilized as an HSP90 warhead. Herein, we report the design, synthesis, and evaluation of the first GM-based HSP90 degraders.

## 2 Materials and methods

### 2.1 Knock-in for generation of HSP90-HiBiT cell lines

Transfection was carried out using the Amaxa Nucleofection system (SF Cell Line Kit, cat. No. V4XC-2032) as previously described (Bhatia et al., 2022). For 2 × 10^5^ K562 (BCR–ABL1+) leukemia cells, 100 pmol of Cas9 protein (Alt-R S. p. HiFi Cas9 nuclease V3, cat. No. 1081060; IDT) was mixed with 120 pmol of gRNA (crRNA:tracrRNA 1:1) and assembled for 20 min at room temperature. Afterward, ssODN was added, and the mixture was combined with the cell suspension (resuspended cells in Nucleofector solution SF) and the electroporation enhancer. The complete volume was gently transferred to the Nucleocuvette module, placed in the 4D-Nucleofector system, and electroporated using the program CA-137. Pre-warmed culture media were quickly added to the cells and transferred to a 96-well plate. Cells were expanded, and monoclonal selection of the edited cells was performed via a semi-solid methylcellulose medium (MethoCult™ H4100 STEMCELL, #04100) supplemented with FCS (Sigma-Aldrich, St. Louis, MO, United States) and penicillin/streptomycin (Invitrogen, Carlsbad, CA, United States). The Nano-Glo^®^ HiBiT Lytic Detection System (#N3030; Promega) was used to measure the edited gene expression for selecting stable HSP90α/β-HiBit tagged clones. Positive clones with stable and higher bioluminescence signals were selected and later subjected to Sanger sequencing to exclude random mutations in the *HSP90AA1* or *HSP90AB1* gene during the integration of HiBit-tag. For experiments, 1 × 10^4^ cells were incubated at indicated concentrations of PROTACs or inhibitor (geldanamycin) at 6 or 24 h time points. Therefore, the treated cells were mixed 1:1 with Nano-Glo^®^ HiBiT Lytic reagent (containing LgBiT Protein (1:100) and Nano-Glo^®^ HiBiT Lytic Substrate (1:50) in Nano-Glo^®^ HiBiT Lytic Buffer). The mixture was incubated for 10 min at room temperature, and luminescence was measured using a Tecan Spark microplate reader. The background luminescence was measured using unedited cells and subtracted from all readings.

### 2.2 Fluorescence polarization assay

An evaluation of the binding affinity of compounds toward the ATP pocket of the HSP90 *N*-terminal domain (NTD) was determined by a competitive binding assay against FITC-labeled geldanamycin (GM) using the HSP90-NTD assay kit (cat. 50293, BPS Biosciences) as previously described (Bhatia et al., 2022). To this end, the inhibitor sample wells were filled with 15 μL of 1× HSP90 assay buffer, 5 μL of 40 mM DTT, 5 μL of 2 mg/ml BSA, 40 μL of H_2_O, 5 μL of FITC-labeled GM (100 nM), and 10 μL of inhibitor at 10 µM or 1 µM. The reaction was initiated by adding 20 μL of HSP90 (17 ng/μL) and incubating at room temperature (protected from light) for 3 h with slow shaking. Background wells (master mix only), negative controls (FITC-labeled GM, buffer, and DMSO), and positive controls (FITC-labeled GM, buffer, DMSO, and HSP90) were also included within the assay plate. Fluorescence polarization was measured at a 470-nm excitation wavelength and 525-nm emission wavelength in a microtiter plate reader (Infinite M1000pro by Tecan). Polarization was calculated using (III − G (I⊥)/[III + G (I⊥)] × 1,000 and a g-factor of 1.187. The percentage of HSP90-bound FITC-labeled GM was calculated using

$$P_{norm}=\frac{P_{Inhibitor}-P_{neg}}{P_{pos}-P_{neg}}*100.$$

### 2.3 Simple western immunoassay

Fluorescent (5×) master mix, DTT, and biotinylated ladder were prepared following the manufacturer’s instructions (BioTechne). Lysates were diluted with 0.1× sample buffer and mixed 5:1 with fluorescent 5× master mix to obtain a target sample concentration of 0.40 μg/μL per well. Samples were then denatured for 5 min at 95°C in a PCR cycler (Gene AMP PCR System 2700, Applied Biosystems). The assay plate was loaded following the manufacturer’s instructions and centrifuged for 5 min at 1,000 g at room temperature. The immunoassay was performed using a 12- to 230-kDa separation module with 25 cartridges (SM-W004, BioTechne). Lysates were separated for 25 min at 375 V, blocked for 5 min with antibody diluent 2, and incubated for 30 min with primary antibody and for 30 min with secondary antibody, subsequently. Primary antibody multiplex mix consisted of 1:100 anti-HSP90 (4877S, Cell Signaling Technology, Danvers, MA) and 1:50 anti-*β*-actin (MAB8929, R&D) diluted in antibody diluent 2. Signals were detected using a JESS anti-rabbit detection module (DM-001, BioTechne) multiplexed with an anti-mouse secondary NIR antibody (043-821, BioTechne).

List of antibodies used

**Table:** 

Target	species	dilution (JESS)	cat. no.
β-actin	Mouse	1:50	MAB8929
HSP90 total	Rabbit	1:100	CST#4877
HSP90α	Rabbit	1:100	CST#8165
HSP90β	Rabbit	1:100	CST#5087

### 2.4 General information and chemistry

Chemicals were obtained from abcr GmbH, Acros Organics, Carbolution Chemicals, Sigma-Aldrich, TCI Chemicals, BLDpharm, or VWR and used without further purification. Technical grade solvents were distilled prior to use. For all HPLC purposes, acetonitrile in HPLC-grade quality (HiPerSolv CHROMANORM, *VWR*) was used. Water was purified with PURELAB^®^ flex (ELGA VEOLIA). Air-sensitive reactions were carried out under an argon atmosphere utilizing standard Schlenk techniques. The uncorrected melting points were determined using a Büchi Melting Point M-560 apparatus. Thin-layer chromatography (TLC) was carried out on prefabricated plates (silica gel 60, F_254_, *Merck*). Components were visualized either by irradiation with ultraviolet light (254 nm or 366 nm) or by staining appropriately. Column chromatography was carried out on silica gel (60 Å, 40–60 μm, Acros Organics). If no solvent is stated, an aqueous solution was prepared with demineralized water. Mixtures of two or more solvents are specified as “solvent A”/“solvent B,” 3/1, *v*/*v*, meaning that 100 ml of the respective mixture consists of 75 ml of “solvent A” and 25 ml of “solvent B.”

### 2.5 Nuclear magnetic resonance spectroscopy (NMR)

Proton (^1^H), carbon (^13^C), and fluorine (^19^F) NMR spectra were recorded either on a Bruker AVANCE 500 MHz at a frequency of 500 MHz (^1^H) and 126 MHz (^13^C) or a Bruker AVANCE III HD 600 MHz at a frequency of 600 MHz (^1^H), 151 MHz (^13^C), and 565 MHz (^19^F). The chemical shifts are given in parts per million (ppm). As solvents, deuterated chloroform (CDCl_3_), deuterated methanol (methanol-*d*_4_), and deuterated dimethyl sulfoxide (DMSO-*d*_6_) were used. The residual solvent signal (CDCl_3_: ^1^H NMR: 7.26 ppm, ^13^C NMR: 77.1 ppm; DMSO-*d*_6_: ^1^H NMR: 2.50 ppm, ^13^C NMR: 39.52 ppm; methanol-*d*_4_: ^1^H NMR: 3.31 ppm, 4.87 ppm, ^13^C NMR: 49.00 ppm) was used for calibration. The multiplicity of each signal is reported as singlet (s), doublet (d), triplet (t), multiplet (m), or combinations thereof. Multiplicities and coupling constants are reported as measured and might disagree with the expected values. For all final compounds, a racemic mixture of R and S thalidomide was used in the reaction; therefore, certain ^1^H and ^13^C NMR signals can occur as two distinct sets of signals due to the presence of diastereomers. When methanol-*d*_*6*_ was used, some NH and OH protons were undetectable due to the H/D exchange.

### 2.6 Mass spectrometry

High-resolution electrospray ionization mass spectra (HRMS-ESI) were acquired with a micrOTOF-Q mass spectrometer (Bruker) with ESI-source coupled with HPLC Dionex UltiMate 3,000 (Thermo Scientific). Low-resolution electrospray ionization mass spectra (LRMS-ESI) were acquired with an Advion expression^®^ compact mass spectrometer (CMS) coupled with an automated TLC plate reader Plate Express^®^ (Advion).

### 2.7 High-performance liquid chromatography (HPLC)

A Thermo Fisher Scientific UltiMateTM 3000 UHPLC system with a Nucleodur 100-5 C18 (250 × 4.6 mm, Macherey Nagel) with a flow rate of 1 ml/min and a temperature of 25°C or a 100-5 C18 (100 × 3 mm, Macherey Nagel) with a flow rate of 0.5 ml/min and a temperature of 25°C with an appropriate gradient was used. For preparative purposes, a Varian ProStar system with a Nucleodur 110-5 C18 HTec (150 × 32 mm, Macherey Nagel) column with 20 ml/min was used. Detection was implemented by UV absorption measurement at a wavelength of *λ* = 220 nm and *λ* = 250 nm. Bidest H_2_O (A) and MeCN (B) were used as eluents with an addition of 0.1% TFA for eluent A. Purity: the purity of all final compounds was 95% or higher. Purity was determined via HPLC with Nucleodur 100-5 C18 (250 × 4.6 mm, Macherey Nagel) at 250 nm. After column equilibration for 5 min, a linear gradient from 5% A to 95% B in 7 min followed by an isocratic regime of 95% B for 10 min was used.

### 2.8 Synthesis

General procedures and data sheets for the final compounds are provided in the following. The data sheets for all intermediates can be found in the Supplementary Materials.

#### 2.8.1 General procedure A

The free diamine (2.0 eq.) was dissolved in ethanol (30 ml per g diamine) and cooled to 0°C. Di-*tert*-butyl dicarbonate (1.1 eq.) was added in small portions to the continuously stirred solution. Afterward, the solution was allowed to warm to room temperature and stirred overnight. After extraction with CH_2_Cl_2_ (3 × 75 ml), the combined organic phases were dried over Na_2_SO_4_ and filtered, and the solvent was removed under reduced pressure. The crude product was purified by column chromatography using CH_2_Cl_2_/MeOH (9/1, *v/v*) with 2% triethylamine.

#### 2.8.2 General procedure B

The free diamine (2.0 eq*.*) was dissolved in CH_2_Cl_2_ (15 ml per g diamine) and cooled to 0°C. Di-*tert*-butyl dicarbonate (1.0 eq*.*) was added in small portions to the continuously stirred solution. Afterward, the solution was allowed to warm to room temperature and stirred overnight. After extraction with CH_2_Cl_2_ (3 × 75 ml), the organic phase was dried over Na_2_SO_4_ and filtered, and the solvent was removed under reduced pressure. The crude product was purified by column chromatography using CH_2_Cl_2_/MeOH (9/1, *v/v*) with 2% triethylamine.

#### 2.8.3 General procedure C

The mono-Boc-protected diamine linker (1.0 eq*.*) was dissolved in DMSO (20 ml per g mono-Boc-protected diamine). *N*,*N*-Diisopropylethylamine (2.0 eq*.*) and 2-(2,6-dioxo-3-piperidinyl)-4-fluoro-1*H*-isoindole-1,3(2*H*)-dione (1.0 eq*.*) were added, and the mixture was stirred at 90°C overnight. After cooling, the solution was poured onto half-saturated brine and extracted with ethyl acetate (3 × 50 ml). The combined organic phases were dried over Na_2_SO_4_, filtered, and concentrated under reduced pressure. The crude product was purified by column chromatography using a mixture of ethyl acetate and cyclohexane.

#### 2.8.4 General procedure D

The Boc-protected thalidomide-linker building block was dissolved in a mixture of CH_2_Cl_2_ and TFA [3/1 (v/v)] and stirred at room temperature until TLC analysis showed complete conversion. After evaporation of the solvents, the TFA-amine salt was dissolved in 5 ml of dichloromethane. After the addition of *N*,*N*-diisopropylethylamine (8.0 eq*.*) and geldanamycin (1.0 eq*.*), the solution was stirred at room temperature overnight. After extraction with ethyl acetate (3 × 25 ml), the solution was dried over Na_2_SO_4_, filtered, and concentrated under reduced pressure. The crude product was purified by column chromatography using a mixture of CH_2_Cl_2_ and acetone.

#### 2.8.5 General procedure E

The Boc-protected thalidomide-linker building block was dissolved in a mixture of CH_2_Cl_2_ and TFA [3/1 (v/v)] and stirred at room temperature until TLC analysis showed complete conversion. After evaporation of the solvents, the TFA-amine salt was dissolved in 5 ml of dichloromethane. After the addition of *N*,*N*-diisopropylethylamine (8.0 eq*.*) and geldanamycin (1.0 eq*.*), the solution was stirred at room temperature overnight. After extraction with ethyl acetate (3 × 25 ml), the solution was dried over Na_2_SO_4_, filtered, and concentrated under reduced pressure. The crude product was purified by column chromatography using a mixture of cyclohexane and ethyl acetate.

#### 2.8.6 (4E,6Z,8S,9S,10E,12S,13R,14S,16R)-19-({2-[2-(2-{[2-(2,6-dioxopiperidin-3-yl)-1,3-dioxoisoindolin-4-yl]amino}ethoxy)ethoxy]ethyl}amino)-13-hydroxy-8,14-dimethoxy-4,10,12,16-tetramethyl-3,20,22-trioxo-2-azabicyclo(16.3.1)docosa-1,4,6,10,18-pentaen-9-yl carbamate (3a)

**3a** was synthesized according to General Procedure D using **2a** as starting material (51 mg, 0.10 mmol, 2.0 eq.). The desired compound **3a** was obtained as a red-brown solid (25 mg, 0.03 mmol). Yield: 50%; mp: 122°C–129°C; R_f_: 0.43 (CH_2_Cl_2_/acetone (3/1) (*v*/*v)*); HRMS-ESI (*m/z*): [M + H]^+^ calcd for C_47_H_61_N_6_O_14_^+^: 933.4240, found: 933.4240; ^1^H NMR (600 MHz, methanol-*d*_4_: *d* 7.55–7.48 (m, 1H), 7.15–7.06 (m, 1H), 7.06–7.01 (m, 1H), 7.01–6.97 (m, 1H), 6.95–6.88 (m, 1H), 6.65–6.57 (m, 1H), 5.90–5.82 (m, 1H), 5.66–5.59 (m, 1H), 5.23–5.14 (m, 1H), 5.03–4.93 (m, 1H), 4.55–4.48 (m, 1H), 3.82–3.64 (m, 10H), 3.61–3.56 (m, 1H), 3.50–3.41 (m, 3H), 3.35–3.32 (m, 3H), 3.28 (s, 3H), 2.89–2.79 (m, 1H), 2.77–2.64 (m, 4H), 2.37–2.27 (m, 1H), 2.14–2.06 (m, 1H), 2.00–1.96 (m, 3H), 1.80–1.70 (m, 4H), 1.70–1.53 (m, 2H), 1.00–0.93 (m, 6H); ^13^C NMR (151 MHz, methanol-*d*_4_) *d* 211.4, 185.6, 185.6, 181.1, 181.0, 174.6, 174.6, 171.4, 171.4, 170.6, 170.6, 169.2, 159.1, 159.1, 148.2, 148.2, 146.7, 146.7, 142.6, 139.3, 137.9, 137.2, 135.4, 135.2, 134.5, 134.5, 133.8, 133.7, 132.9, 132.7, 129.6, 127.3, 127.2, 125.5, 118.3, 112.1, 112.1, 111.3, 110.1, 110.0, 109.3, 109.2, 83.0, 82.1, 82.0, 74.2, 74.2, 71.7, 71.6, 71.6, 70.7, 70.7, 70.6, 70.2, 70.2, 57.5, 57.5, 56.9, 56.1, 50.2, 50.2, 49.6, 49.4, 49.3, 49.1, 49.0, 48.9, 48.7, 48.6, 46.2, 46.1, 43.3, 43.3, 35.8, 35.5, 34.4, 34.3, 33.9, 33.7, 33.1, 32.3, 32.3, 32.1, 31.9, 30.9, 30.8, 30.5, 29.5, 23.9, 23.9, 23.7, 22.7, 20.9, 13.6, 13.5, 12.4.

#### 2.8.7 (4E,6Z,8S,9S,10E,12S,13R,14S,16R)-19-[(2-{2-[2-(2-{[2-(2,6-dioxopiperidin-3-yl)-1,3-dioxoisoindolin-4-yl]amino}ethoxy)ethoxy]ethoxy}ethyl)amino]-13-hydroxy-8,14-dimethoxy-4,10,12,16-tetramethyl-3,20,22-trioxo-2-azabicyclo[16.3.1]docosa-1(21),4,6,10,18-pentaen-9-yl carbamate (3b)

**3b** was synthesized according to General Procedure D using **2b** as starting material (56 mg, 0.10 mmol, 2.0 eq.). The desired compound **3b** was obtained as a red-brown solid (28 mg, 0.03 mmol). Yield: 55%; mp: 117°C–119°C; R_f_: 0.65 (CH_2_Cl_2_/acetone (1/1) (*v*/*v)*); HRMS-ESI (m/z): [M + Na]^+^ calcd for C_49_H_64_N_6_O_15_Na^+^: 999.4322, found: 999.4322; ^1^H NMR (600 MHz, methanol-*d*_4_) *d* 7.54–7.48 (m, 1H), 7.14–7.07 (m, 1H), 7.07–6.96 (m, 3H), 6.62 (d, *J* = 11.4 Hz, 1H), 5.90–5.83 (m, 1H), 5.64–5.59 (m, 1H), 5.22 (s, 1H), 5.05–4.99 (m, 1H), 4.55–4.50 (m, 1H), 3.74–3.64 (m, 14H), 3.62–3.56 (m, 1H), 3.50–3.43 (m, 3H), 3.35–3.32 (m, 3H), 3.30 (s, 3H), 2.89–2.82 (m, 1H), 2.78–2.65 (m, 4H), 2.34–2.26 (m, 1H), 2.15–2.10 (m, 1H), 2.01–1.97 (m, 3H), 1.74–1.71 (m, 4H), 1.68–1.53 (m, 2H), 0.98–0.93 (m, 6H); ^13^C NMR (151 MHz, methanol-*d*_4_) *d* 211.7, 185.7, 181.0, 174.8, 171.5, 170.7, 170.6, 169.3, 159.1, 148.1, 146.8, 142.6, 137.9, 137.3, 135.3, 134.4, 133.8, 129.6, 127.2, 118.3, 118.3, 112.1, 111.2, 110.2, 109.2, 83.0, 81.9, 74.3, 71.7, 71.7, 71.7, 71.7, 71.6, 71.4, 71.4, 70.6, 70.6, 70.6, 70.2, 57.5, 56.8, 56.0, 50.2, 49.6, 49.4, 49.3, 49.1, 49.0, 48.9, 48.7, 48.6, 46.3, 43.3, 35.8, 34.4, 33.6, 33.0, 32.2, 32.1, 30.7, 30.4, 29.6, 29.5, 23.9, 23.8, 23.7, 22.7, 20.9, 13.6, 12.5.

#### 2.8.8 (4E,6Z,8S,9S,10E,12S,13R,14S,16R)-19-({3-[4-(3-{[2-(2,6-dioxopiperidin-3-yl)-1,3-dioxoisoindolin-4-yl]amino}propoxy)butoxy]propyl}amino)-13-hydroxy-8,14-dimethoxy-4,10,12,16-tetramethyl-3,20,22-trioxo-2-azabicyclo[16.3.1]docosa-1(21),4,6,10,18-pentaen-9-yl carbamate (3c)

**3c** was synthesized according to General Procedure D using **2c** as starting material (56 mg, 0.10 mmol, 2.0 eq.). The desired compound **3c** was obtained as a red-brown solid (40 mg, 0.04 mmol). Yield: 78%; mp: 145°C–150°C; R_f_: 0.42 (CH_2_Cl_2_/acetone (3/1) (*v*/*v)*); HRMS-ESI (m/z): [M + H]^+^ calcd for C_51_H_69_N_6_O_14_^+^: 989.4833, found: 989.4866; ^1^H NMR (600 MHz, methanol-*d*_4_) *d* 7.53–7.46 (m, 1H), 7.11–7.05 (m, 1H), 7.04–6.96 (m, 3H), 6.65–6.57 (m, 1H), 5.89–5.83 (m, 1H), 5.67–5.59 (m, 1H), 5.23–5.19 (m, 1H), 5.04–4.97 (m, 1H), 4.54–4.49 (m, 1H), 3.71–3.53 (m, 8H), 3.50–3.44 (m, 5H), 3.43–3.38 (m, 2H), 3.33 (s, 3H), 3.30–3.27 (m, 3H), 2.90–2.80 (m, 1H), 2.79–2.63 (m, 4H), 2.39–2.31 (m, 1H), 2.15–2.07 (m, 1H), 1.98 (s, 3H), 1.94–1.86 (m, 4H), 1.75–1.63 (m, 8H), 1.62–1.55 (m, 1H), 0.99–0.94 (m, 6H); ^13^C NMR (151 MHz, methanol-*d*_4_) *d* 211.8, 185.7, 185.7, 180.8, 180.8, 174.9, 174.8, 174.8, 171.5, 171.4, 170.7, 170.6, 170.6, 169.4, 169.3, 159.1, 159.1, 148.3, 148.2, 146.8, 142.8, 137.9, 137.2, 137.2, 135.3, 134.4, 133.8, 129.6, 127.2, 118.0, 117.9, 111.8, 110.9, 110.9, 109.4, 109.1, 83.0, 82.0, 74.2, 72.2, 72.2, 72.0, 71.9, 70.6, 70.3, 70.3, 69.6, 69.6, 57.5, 56.9, 56.9, 56.0, 50.2, 50.1, 49.6, 49.4, 49.3, 49.1, 49.0, 48.9, 48.7, 48.6, 45.6, 45.6, 41.4, 41.3, 41.2, 35.9, 34.4, 33.8, 33.0, 32.2, 32.1, 31.4, 30.9, 30.7, 30.6, 30.5, 30.5, 30.4, 30.4, 29.6, 29.5, 27.6, 27.6, 27.4, 23.8, 23.7, 22.7, 20.9, 14.3, 13.6, 12.5.

#### 2.8.9 (4E,6Z,8S,9S,10E,12S,13R,14S,16R)-19-[(3-{2-[2-(3-{[2-(2,6-dioxopiperidin-3-yl)-1,3-dioxoisoindolin-4-yl]amino}propoxy)ethoxy]ethoxy}propyl)amino]-13-hydroxy-8,14-dimethoxy-4,10,12,16-tetramethyl-3,20,22-trioxo-2-azabicyclo[16.3.1]docosa-1(21),4,6,10,18-pentaen-9-yl carbamate (3d)

**3d** was synthesized according to General Procedure D using **2d** as starting material (58 mg, 0.10 mmol, 2.0 eq.). The desired compound **3d** was obtained as a red-brown solid (41 mg, 0.04 mmol). Yield: 78%; mp: 123°C–132°C; R_f_: 0.77 (CH_2_Cl_2_/acetone (1/1) (*v*/*v)*); HRMS-ESI (m/z): [M + H]^+^ calcd for C_51_H_69_N_6_O_15_^+^: 1,005.4815, found: 1,005.4815; ^1^H NMR (600 MHz, methanol-*d*_4_) *d* 7.54–7.47 (m, 1H), 7.11–7.04 (m, 1H), 7.04–7.00 (m, 1H), 7.00–6.95 (m, 2H), 6.65–6.58 (m, 1H), 5.89–5.83 (m, 1H), 5.67–5.59 (m, 1H), 5.25–5.20 (m, 1H), 5.04–4.97 (m, 1H), 4.56–4.49 (m, 1H), 3.74–3.67 (m, 4H), 3.67–3.54 (m, 10H), 3.48–3.43 (m, 1H), 3.43–3.37 (m, 2H), 3.34 (s, 3H), 3.29–3.28 (m, 3H), 2.89–2.81 (m, 1H), 2.79–2.63 (m, 4H), 2.38–2.29 (m, 1H), 2.16–2.09 (m, 2H), 2.01–1.97 (m, 3H), 1.94–1.86 (m, 4H), 1.73 (s, 4H), 1.69–1.54 (m, 2H), 0.99–0.93 (m, 6H); ^13^C NMR (151 MHz, methanol-*d*_4_) *d* 211.8, 210.5, 185.7, 185.7, 180.8, 174.9, 174.8, 171.5, 171.5, 170.7, 170.6, 170.6, 169.3, 169.3, 159.1, 159.1, 159.1, 159.1, 148.2, 148.2, 146.8, 146.7, 142.8, 137.9, 137.3, 135.3, 134.4, 133.8, 132.8, 129.6, 127.2, 127.2, 118.0, 111.8, 111.8, 110.9, 110.8, 109.5, 109.1, 83.0, 82.0, 74.2, 71.6, 71.6, 71.6, 71.5, 71.5, 71.4, 70.6, 70.5, 70.5, 70.0, 69.9, 57.5, 57.5, 56.9, 56.1, 50.2, 50.1, 49.4, 49.3, 49.1, 49.0, 48.9, 48.7, 48.6, 45.3, 45.2, 45.2, 41.3, 41.2, 41.1, 35.9, 34.4, 33.8, 33.0, 32.2, 32.1, 30.9, 30.7, 30.7, 30.6, 30.5, 30.5, 30.4, 30.4, 29.5, 23.9, 23.7, 22.7, 20.9, 14.3, 13.6, 12.5.

#### 2.8.10 (4E,6Z,8S,9S,10E,12S,13R,14S,16R)-19-[(6-{[2-(2,6-dioxopiperidin-3-yl)-1,3-dioxoisoindolin-4-yl]amino}hexyl)amino]-13-hydroxy-8,14-dimethoxy-4,10,12,16-tetramethyl-3,20,22-trioxo-2-azabicyclo[16.3.1]docosa-1(21),4,6,10,18-pentaen-9-yl carbamate (3e)

**3e** was synthesized according to General Procedure E using **2e** as starting material (47 mg, 0.10 mmol, 2.0 eq*.).* The desired compound **3e** was obtained as a red-brown solid (24 mg, 0.03 mmol). Yield: 52%; mp: 158°C–162°C; R_f_: 0.64 (ethyl acetate); HRMS-ESI (m/z): [M + Na]^+^ calcd for C_47_H_60_N_6_O_12_Na^+^: 923.4167, found: 923.4161; ^1^H NMR (600 MHz, methanol-*d*_4_) *d* 7.56–7.50 (m, 1H), 7.14–7.09 (m, 1H), 7.05–6.99 (m, 3H), 6.62 (t, *J* = 11.7 Hz, 1H), 5.90–5.84 (m, 1H), 5.62–5.57 (m, 1H), 5.24–5.20 (m, 1H), 5.06–5.01 (m, 1H), 4.56–4.51 (m, 1H), 3.63–3.58 (m, 1H), 3.55–3.51 (m, 2H), 3.49–3.44 (m, 1H), 3.33–3.32 (m, 3H), 3.30 (s, 3H), 2.89–2.82 (m, 1H), 2.78–2.64 (m, 4H), 2.35–2.25 (m, 1H), 2.15–2.08 (m, 1H), 1.72–1.61 (m, 6H), 1.60–1.53 (m, 2H), 1.53–1.43 (m, 5H), 1.36–1.26 (m, 6H), 1.01–0.94 (m, 6H); ^13^C NMR (151 MHz, methanol-*d*_4_) *d* 185.8, 185.8, 180.9, 180.9, 174.9, 174.8, 171.6, 171.6, 170.8, 170.8, 169.4, 169.4, 159.1, 159.1, 148.4, 148.3, 146.6, 142.8, 137.9, 137.3, 135.4, 134.4, 133.8, 133.8, 130.9, 130.4, 129.6, 127.8, 127.2, 118.1, 111.9, 111.9, 110.9, 109.6, 109.1, 83.0, 81.9, 77.4, 74.3, 72.2, 67.4, 62.0, 57.5, 56.8, 50.2, 50.2, 49.4, 49.3, 49.1, 49.0, 48.9, 48.7, 48.6, 46.3, 46.2, 46.2, 43.3, 43.3, 43.2, 43.1, 38.9, 35.8, 35.3, 34.5, 33.7, 33.0, 32.8, 32.2, 32.2, 31.8, 30.8, 30.8, 30.7, 30.7, 30.7, 30.6, 30.4, 30.3, 30.2, 30.1, 30.0, 28.1, 27.5, 27.4, 27.3, 26.9, 26.1, 23.8, 23.8, 23.7, 22.9, 22.7, 21.0, 20.9, 20.3, 19.9, 14.3, 13.6, 12.5.

#### 2.8.11 (4E,6Z,8S,9S,10E,12S,13R,14S,16R)-19-[(8-{[2-(2,6-dioxopiperidin-3-yl)-1,3-dioxoisoindolin-4-yl]amino}octyl)amino]-13-hydroxy-8,14-dimethoxy-4,10,12,16-tetramethyl-3,20,22-trioxo-2-azabicyclo[16.3.1]docosa-1(21),4,6,10,18-pentaen-9-yl carbamate (3f)

**3f** was synthesized according to General Procedure E using **2f** as starting material (50 mg, 0.10 mmol, 2.0 eq.). The desired compound **3f** was obtained as a red-brown solid (26 mg, 0.03 mmol). Yield: 52%; mp: 155°C–162°C; R_f_: 0.28 (cyclohexane/ethyl acetate (1/3) (*v*/*v)*); HRMS-ESI (m/z): [M + Na]^+^ calcd for C_49_H_64_N_6_O_12_Na^+^: 951.4480, found: 951.4474; ^1^H NMR (600 MHz, methanol-*d*_4_) *d* 7.57–7.51 (m, 1H), 7.14–7.09 (m, 1H), 7.07–7.00 (m, 3H), 6.62 (t, *J* = 11.4 Hz, 1H), 5.90–5.84 (m, 1H), 5.62–5.57 (m, 1H), 5.22 (s, 1H), 5.07–5.01 (m, 1H), 4.56–4.52 (m, 1H), 3.64–3.57 (m, 1H), 3.56–3.50 (m, 2H), 3.50–3.44 (m, 1H), 3.34 (s, 3H), 3.30 (s, 3H), 2.88–2.80 (m, 1H), 2.79–2.65 (m, 4H), 2.35–2.25 (m, 1H), 2.15–2.08 (m, 1H), 1.99 (s, 3H), 1.83–1.77 (m, 1H), 1.73 (s, 3H), 1.70–1.61 (m, 5H), 1.61–1.52 (m, 2H), 1.46–1.36 (m, 9H), 0.99–0.96 (m, 6H); ^13^C NMR (151 MHz, methanol-*d*_4_) *d* 185.8, 180.9, 174.8, 171.6, 170.8, 169.4, 159.1, 159.1, 148.4, 148.3, 146.6, 142.9, 137.9, 137.3, 135.3, 134.4, 133.8, 133.8, 132.5, 130.4, 129.7, 127.8, 127.2, 118.0, 115.8, 111.8, 110.9, 109.5, 109.1, 83.0, 81.9, 77.4, 74.3, 72.2, 67.4, 57.6, 56.8, 52.5, 50.2, 49.6, 49.4, 49.3, 49.1, 49.0, 48.9, 48.7, 48.6, 46.3, 46.3, 43.5, 43.4, 35.8, 34.5, 33.6, 33.1, 32.8, 32.2, 31.7, 30.8, 30.8, 30.7, 30.6, 30.4, 30.3, 30.2, 30.1, 30.1, 30.1, 30.1, 30.0, 29.1, 27.7, 27.7, 27.5, 27.5, 23.8, 23.7, 23.7, 23.0, 22.9, 22.7, 20.9, 20.3, 19.9, 19.3, 14.3, 13.7, 12.4.

#### 2.8.12 (4E,6Z,8S,9S,10E,12S,13R,14S,16R)-19-[(10-{[2-(2,6-dioxopiperidin-3-yl)-1,3-dioxoisoindolin-4-yl]amino}decyl)amino]-13-hydroxy-8,14-dimethoxy-4,10,12,16-tetramethyl-3,20,22-trioxo-2-azabicyclo[16.3.1]docosa-1(21),4,6,10,18-pentaen-9-yl carbamate (3g)

**3g** was synthesized according to General Procedure E using **2g** as starting material (53 mg, 0.10 mmol, 2.0 eq.). The desired compound **3g** was obtained as a red-brown solid (22 mg, 0.02 mmol). Yield: 44%; mp: 165°C–169°C; R_f_: 0.22 (cyclohexane/ethyl acetate (1/3) (*v*/*v)*); HRMS-ESI (m/z): [M + Na]^+^ calcd for C_51_H_68_N_6_O_12_Na^+^: 979.4793, found: 979.4787; ^1^H NMR (600 MHz, methanol-*d*_4_) *d* 7.57–7.51 (m, 1H), 7.15–7.09 (m, 1H), 7.07–7.00 (m, 3H), 6.65–6.58 (m, 1H), 5.90–5.84 (m, 1H), 5.62–5.56 (m, 1H), 5.22 (s, 1H), 5.06–5.01 (m, 1H), 4.56–4.52 (m, 1H), 3.64–3.57 (m, 1H), 3.55–3.44 (m, 3H), 3.34 (s, 3H), 3.30 (s, 3H), 2.90–2.83 (m, 1H), 2.79–2.66 (m, 4H), 2.35–2.25 (m, 1H), 2.15–2.07 (m, 1H), 1.99 (s, 3H), 1.86–1.77 (m, 1H), 1.73 (d, *J* = 1.3 Hz, 3H), 1.70–1.61 (m, 5H), 1.60–1.52 (m, 2H), 1.47–1.34 (m, 13H), 1.03–0.95 (m, 6H); ^13^C NMR (151 MHz, methanol-*d*_4_) *d* 185.8, 180.9, 174.8, 171.6, 170.8, 169.4, 159.1, 159.1, 148.4, 148.3, 146.6, 142.9, 137.9, 137.3, 135.3, 134.4, 133.9, 132.4, 129.7, 127.2, 118.0, 111.8, 110.9, 109.5, 109.1, 83.0, 81.9, 77.4, 74.4, 72.2, 67.4, 62.0, 57.6, 56.8, 50.2, 49.6, 49.4, 49.3, 49.1, 49.0, 48.9, 48.7, 48.6, 46.5, 46.3, 43.5, 43.4, 38.9, 35.8, 34.6, 33.6, 33.1, 32.8, 32.2, 32.2, 31.4, 30.8, 30.8, 30.7, 30.4, 30.3, 30.3, 30.2, 30.2, 30.1, 27.8, 27.6, 26.9, 23.8, 23.7, 22.9, 22.7, 20.9, 20.3, 19.9, 14.3, 13.7, 12.4.

#### 2.8.13 (4E,6Z,8S,9S,10E,12S,13R,14S,16R)-13-hydroxy-8,14-dimethoxy-4,10,12,16-tetramethyl-19-({2-[2-(2-{[2-(1-methyl-2,6-dioxopiperidin-3-yl)-1,3-dioxoisoindolin-4-yl]amino}ethoxy)ethoxy]ethyl}amino)-3,20,22-trioxo-2-azabicyclo[16.3.1]docosa-1(21),4,6,10,18-pentaen-9-yl carbamate (nc-3a)

**Nc-3a** was synthesized according to General Procedure D using **6** as starting material (52 mg, 0.10 mmol, 2.0 *eq*.). The desired compound **nc-3a** was obtained as a red brown solid (10 mg, 0.01 mmol). Yield: 20%; mp: 123–130°C; R_f_: 0.28 (cyclohexane/ethyl acetate (9/1) (*v*/*v)*); HRMS-ESI (m/z): [M + Na]^+^ calcd for C_48_H_62_N_6_O_14_Na^+^: 969.4222, found: 969.4216; ^1^H NMR (600 MHz, methanol-*d*_4_) *d* 7.55–7.49 (m, 1H), 7.13–7.08 (m, 1H), 7.08–7.03 (m, 1H), 7.03–6.98 (m, 1H), 6.98–6.93 (m, 1H), 6.62 (t, *J* = 11.4 Hz, 1H), 5.90–5.84 (m, 1H), 5.63–5.58 (m, 1H), 5.18 (s, 1H), 5.07–5.01 (m, 1H), 4.55–4.47 (m, 1H), 3.80–3.75 (m, 2H), 3.74–3.66 (m, 8H), 3.62–3.52 (m, 1H), 3.51–3.46 (m, 2H), 3.46–3.41 (m, 1H), 3.35 (s, 1H), 3.34–3.32 (m, 3H), 3.30–3.28 (m, 2H), 3.12 (s, 3H), 2.90–2.83 (m, 2H), 2.74–2.64 (m, 3H), 2.35–2.28 (m, 1H), 2.14–2.06 (m, 1H), 2.00 (s, 3H), 1.82–1.75 (m, 1H), 1.73 (s, 3H), 1.66–1.61 (m, 1H), 1.59–1.52 (m, 1H), 1.01–0.94 (m, 6H); ^13^C NMR (151 MHz, methanol-*d*_4_) *d* 211.4, 185.7, 185.6, 181.0, 181.0, 173.7, 171.3, 170.7, 170.7, 170.6, 169.3, 169.3, 159.1, 148.2, 146.8, 142.6, 137.9, 137.2, 135.4, 134.5, 133.8, 133.8, 132.6, 130.9, 129.6, 127.2, 120.6, 118.3, 112.1, 111.3, 110.1, 109.2, 82.9, 82.0, 74.3, 73.4, 71.7, 71.7, 71.7, 71.4, 70.7, 70.6, 70.3, 70.3, 69.8, 69.8, 62.2, 57.5, 57.5, 56.8, 56.8, 56.8, 56.1, 50.9, 50.9, 49.8, 49.6, 49.4, 49.3, 49.1, 49.0, 48.9, 48.7, 48.6, 46.2, 46.2, 43.3, 43.3, 40.8, 40.7, 36.5, 35.7, 34.5, 33.6, 33.1, 32.6, 32.5, 32.1, 31.4, 30.8, 30.8, 30.6, 30.6, 30.5, 30.3, 29.5, 28.1, 27.4, 26.9, 25.6, 23.7, 23.1, 23.1, 22.7, 16.8, 14.3, 13.6, 13.6, 12.4.

## 3 Results

**FIGURE 1:**
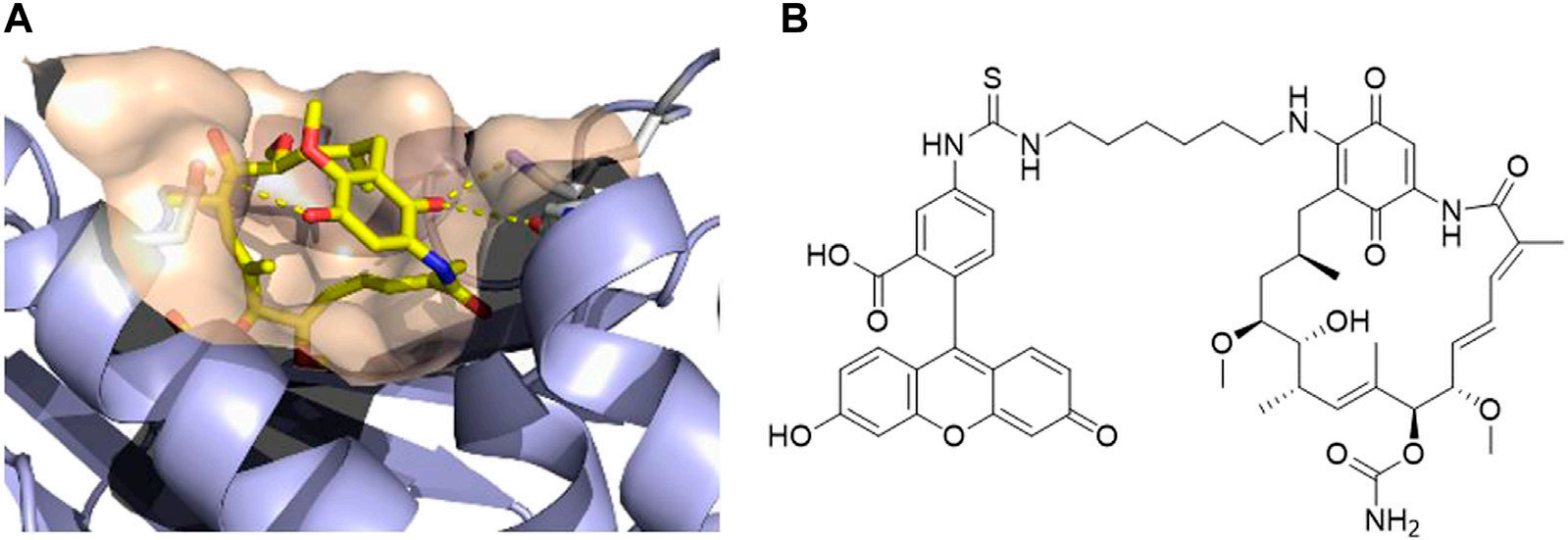
**(A)** Co-crystal structure of human HSP90α in complex with geldanamycin (PDB ID: 1YET) (Stebbins et al., 1997). **(B)** Structure of the fluorescent probe FITC-geldanamycin (Llauger-Bufi et al., 2003).

After analyzing the crystal structure of GM bound to human HSP90α, we observed that the methoxy group at the 17-position is exposed to the solvent (Figure 1A). Therefore, this position can serve as a suitable exit vector for the assembly of PROTACs. This is further corroborated by the commercially available fluorescent probe FITC-GM, which is commonly used in fluorescence polarization assays to investigate the binding of HSP90 inhibitors to the *N*-terminal ATP binding pocket (Llauger-Bufi et al., 2003). As shown in Figure 1B, the FITC label was introduced via an alkyl linker into the 17-position of GM. The same position was utilized in the conjugation of GM with small molecules, such as estradiol, testosterone, and ferulic acid (Kuduk et al., 1999; Kuduk et al., 2000; Li et al., 2019). Hence, we designed a series of potential PROTACs by attaching a cereblon (CRBN)-recruiting, pomalidomide-derived ligand via various alkyl- or PEG-based linkers of different chain lengths into the 17-position of GM.

**SCHEME 1:**
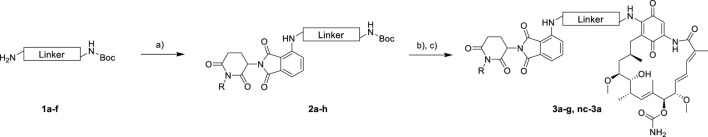
Synthesis of geldanamycin-based HSP90 PROTACs. Reagents and conditions: **(A)** 4-fluorothalidomide, DIPEA, DMSO, 90°C, overnight, 39%–60%. **(B)** TFA, CH_2_Cl_2_, r.t., 3 h. **(C)** Geldanamycin, DIPEA, CH_2_Cl_2_, r.t., overnight, 44%–78% over two steps.

The synthesis of the potential HSP90 degraders is outlined in Scheme 1. First, the commercially available diamine linkers were dissolved in ethanol or dichloromethane. The addition of an excess of di-*tert*-butyl dicarbonate to the mixture at 0°C and stirring overnight gave mono-Boc protected diamines **1a–f** (see Supplementary Materials for synthetic details). In the next step, 2-(2,6-dioxo-piperidin-3-yl)-4-fluoroisoindoline-1,3-dione and diisopropylethylamine (DIPEA) were dissolved in DMSO. After the addition of the mono-Boc protected linkers, the reaction was heated to 90°C overnight, yielding the pomalidomide-linker derivatives **2a–h**. Finally, the target compounds were obtained by acidolytic Boc-deprotection followed by the substitution reaction of commercially available GM with the free amine of the pomalidomide-linker-NH_2_ building blocks. In addition, a non-degrading control compound was prepared by *N*-methylation of the glutarimide ring (see Supplementary Materials for synthetic details). After purification by column chromatography, the final PROTACs shown in Table 1 were obtained in >95% purity.

**TABLE 1:** Overview of the final compounds with their respective linkers.

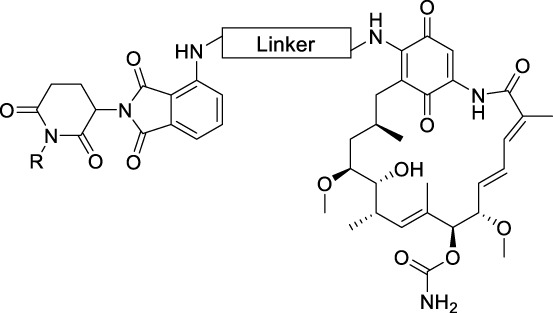		

Compound	Linker	R
**3a**	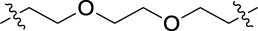	H
**3b**	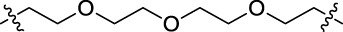	H
**3c**	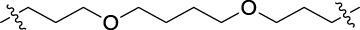	H
**3d**	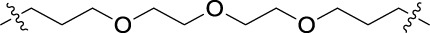	H
**3e**	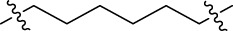	H
**3f**	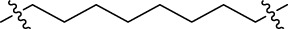	H
**3g**	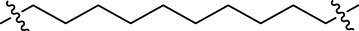	H
**nc-3a**	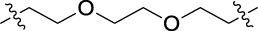	CH_3_

**FIGURE 2:**
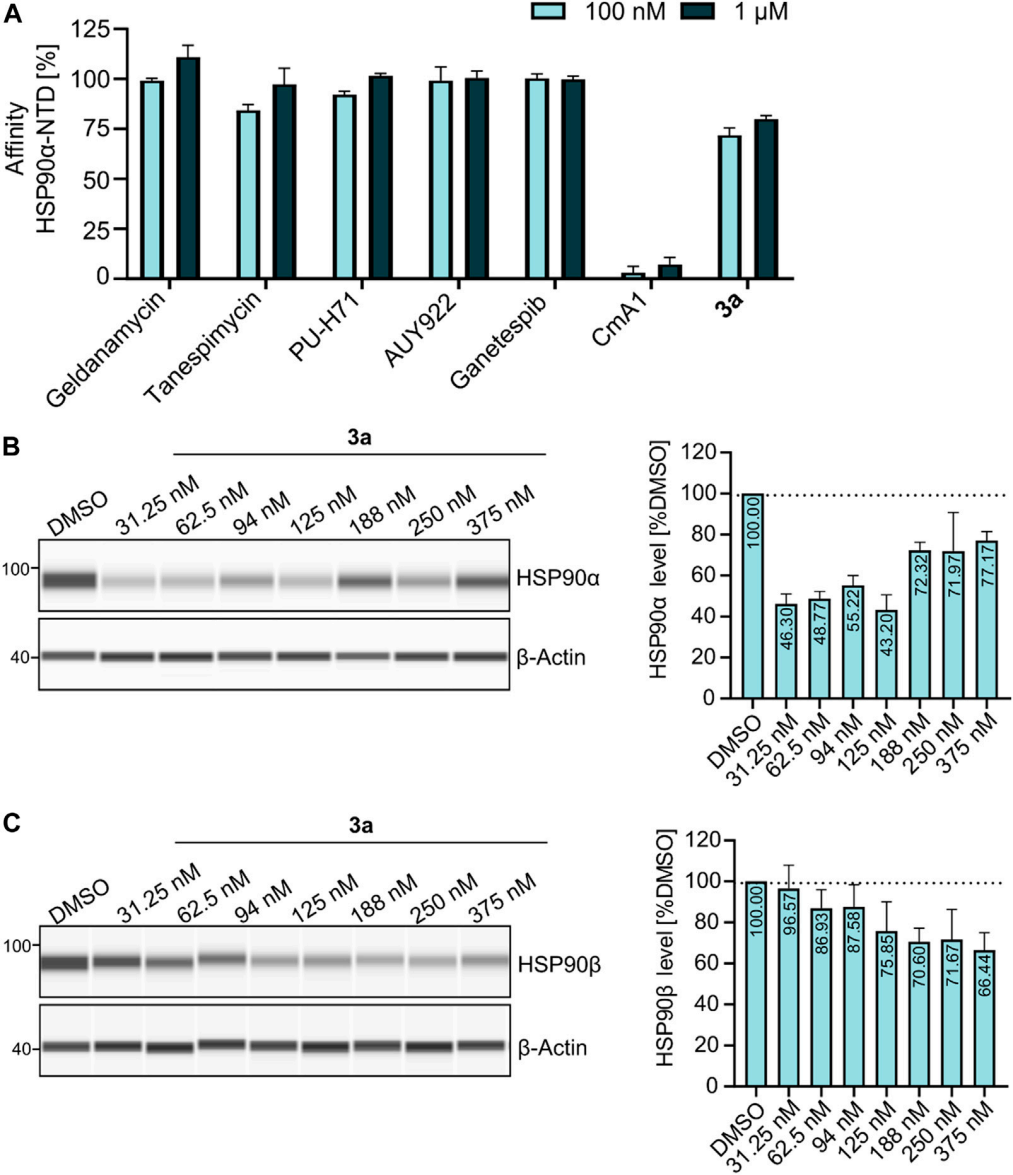
**(A)** Fluorescence polarization assay showing that **3a** is binding to HSP90α-NTD as compared to other HSP90-NTD targeting inhibitors (geldanamycin, tanespimycin, PU-H71, AUY922, and ganetespib), whereas HSP90 C-terminal domain targeting inhibitor coumermycin A1 (CmA1) served as a negative control for the assay. Data were normalized using bound FITC-labeled geldanamycin (100 nM) as the negative control (0%), while free spinning or unbound FITC-labeled geldanamycin as the positive control (100%). **(B–C)** Exemplary JESS run with increasing concentrations of **3a**, showing the degradation of **(B)** HSP90α and **(C)** HSP90β.

**FIGURE 3:**
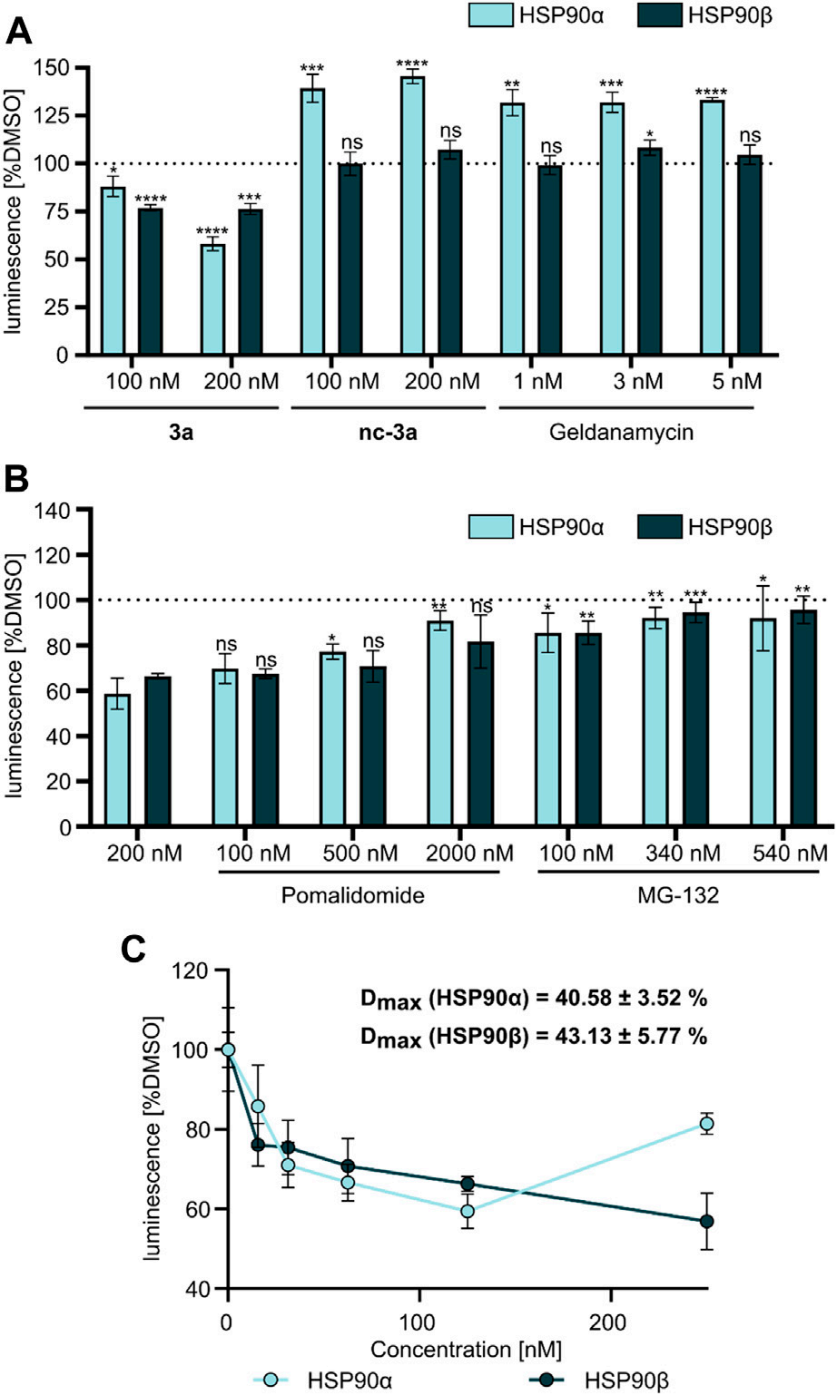
**(A)** NanoGlo lytic assay using HSP90α/β HiBiT-tagged K562 cells treated with **3a**, **nc–3a**, and geldanamycin for 24 h. **(B)** NanoGlo lytic assay using HiBiT-tagged K562 cells treated with **3a** alone or in combination with pomalidomide or MG-132 for 24 h. **(C)** NanoGlo lytic assay using K562 HiBiT cells treated with **3a** at indicated concentrations (15–250 nM) measured after 24 h.

At first, a pre-selection was made to determine which PROTAC is capable of degrading HSP90α, *ß*, or both. For this purpose, K562 leukemia cells were treated with respective PROTACs at a fixed concentration (1 µM) and incubated for 24 h. Quantitative simple western immunoassays showed that **3a** with a PEG2-based linker induced the strongest degradation of HSP90α and HSP90β (Supplementary Figure S1A, Supplementary Materials). Consequently, this PROTAC was selected for further detailed biological experiments. The binding affinity was determined in a fluorescence polarization assay based on the displacement of the fluorescent probe FITC-GM to validate the binding of PROTAC to HSP90. **3a** was found to bind the ATP binding pocket of the HSP90α-*N*-terminal domain (NTD), with only slightly lower affinity than GM (Figure 2A). The degradation efficiency was investigated by automated capillary Western blot in a wide concentration range (Figures 2B,C and Supplementary Figures S1B,C; Supplementary Materials) to identify the optimal concentration range. HSP90α was degraded, especially at low levels of **3a**, in a range between 30 nM and 125 nM. Further quantification revealed a degradation maximum (D_MAX_) of 57% against HSP90α (Figure 2B, Supplementary Figure S1B, Supplementary Materials). At higher concentrations from 188 nM, a renewed increase in HSP90α was seen. Conversely, HSP90β is more degraded with increasing concentration of **3a**, and a plateau is reached above 188 nM. Further quantification revealed a D_max_ for HSP90β of 34% (Figure 2C and Supplementary Figure S1C, Supplementary Materials). Next, we generated HSP90α/β HiBiT-tagged models using CRISPR/Cas9 gene knock-in in K562 cells (Table 2) (Sinatra et al., 2022). This system allows the quantification of proteins down to endogenous levels. The degradation efficiency of HSP90α/β after incubation of **3a** was then determined using a sensitive bioluminescent-based assay utilizing the HiBit–LgBiT complementation technology. The concentration range for the HiBit assay was picked based on the Western blot data and the incubation time from the kinetic studies, which showed that 24 h treatment of **3a** causes more degradation of HSP90β than 6 h treatment (Supplementary Figure S1D, Supplementary Materials). The non-degrading PROTAC analog **nc**–**3a** was used as control, which bears a methyl group at the glutarimide moiety of the pomalidomide. This substitution prevents the pomalidomide derivative from binding to its target cereblon, and therefore, **nc-3a** can only bind to HSP90. In the case of HSP90β, the use of **nc–3a** led to no degradation, as expected, whereas, interestingly, a significant increase in the expression of the stress-inducible isoform HSP90α was noticed. The same effect was also observed with the inhibitor GM, which also caused a significant increase in the HSP90α expression (Figure 3A). Next, K562 cells were pretreated with the CRBN ligand pomalidomide followed by **3a** treatment to demonstrate that binding to CRBN is involved in the reduction of HSP90 protein levels. Increasing the concentration of pomalidomide resulted in a gradual rescue of HSP90α degradation, while a weak trend in the HSP90β rescue was noticed (Figure 3B). Furthermore, K562 cells were pre-incubated with the proteasome inhibitor MG-132 and subsequently treated with **3a**. This also resulted in the inhibition of HSP90α/β degradation (Figure 3B), thereby confirming that the ubiquitin–proteasome system is involved in the observed degradation of HSP90α/β. Taken together, these results indicate that the degradation of HSP90 is mediated *via* the ubiquitin–proteasome pathway. Using the HSP90-HiBiT system, a D_max_ of 41% for HSP90α and 43% for HSP90β was determined for **3a** (Figure 3C). However, in line with the Western blot results, the HSP90α level started to increase between 100 nM and 200 nM, compared to HSP90β. The discrepancy in the degradation of HSP90α and HSP90β can be attributed to the fact that HSP90α is an isoform that is induced under stressful conditions, whereas HSP90β is a constitutively expressed isoform. At higher concentrations, besides acting as a degrader, **3a** can inhibit both HSP90 isoforms, which can induce cellular stress, resulting in the induction of the stress-inducible isoform HSP90α, while HSP90β levels remain unaffected.

**TABLE 2:** Trans-activating CRISPR RNA (crRNA) and repair template (single-stranded oligodeoxynucleotide or ssODN) sequences used to generate stable HSP90α/β-HiBit tagged knock-in cells. The left and the right homology arms for the respective target genes in the ssODN sequence are represented in upper case, separated by the HiBit-tag, represented in lower case.

Construct	Sequence
crRNA *HSP90AA1* (HSP90α)	5’-AGU​AGA​CUA​AUC​UCU​GGC​UGG​UUU​UAG​AGC​UAU​GCU-3’
crRNA *HSP90AB1* (HSP90β)	5’-UCG​CAU​GGA​AGA​AGU​CGA​UUG​UUU​UAG​AGC​UAU​GCU-3’
tracrRNA	Universal 67mer
ssODN HSP90AA1	5’- TGC​CAC​CCC​TTG​AAG​GAG​ATG​ACG​ACA​CAT​CAC​GCA​TGG​AAG​AAG​TAG​ACg​tga​gcg​gct​ggc​ggc​tgt​tca​aga​aga​tta​gcT​AAT​CTC​TGG​CTG​AGG​GAT​GAC​TTA​CCT​GTT​CAG​TAC​TCT​ACA​ATT​CCT​C-3’
ssODN HSP90AB1	5’- TCC​CCC​CTC​TCG​AGG​GCG​ATG​AGG​ATG​CGT​CTC​GCA​TGG​AAG​AAG​TCG​ATg​tga​gcg​gct​ggc​ggc​tgt​tca​aga​aga​tta​gcT​AAG​TTA​GAA​GTT​CAT​AGT​TGA​AAA​ACT​TGT​GCC​CTT​GTA​TAG​TGT​CCC​C-3’

In summary, we have designed and synthesized a first-in-class series of GM-based HSP90 degraders. The most promising PROTAC **3a** effectively degraded HSP90α and HSP90β levels *via* the ubiquitin–proteasome pathway. Using (endogenous) HSP90-labeled HiBiT–LgBiT complementation assay for the first time, we showed that **3a** was capable of downregulating HSP90, while normal HSP90 inhibition by GM or the non-degrading control compound **nc–3a** led to a significant upregulation of the stress-inducible isoform HSP90α. Thus, targeted degradation of HSP90 via GM-based PROTACs might provide an alternative approach to target HSP90-driven diseases.

## Data Availability

The raw data supporting the conclusion of this article will be made available by the authors, without undue reservation.
