# Supplementary material for: Development of the first geldanamycin-based HSP90 degraders
Source: Front Chem. 2023 Jun 28;11:1219883. doi: 10.3389/fchem.2023.1219883 (PMC10336212; doi:10.3389/fchem.2023.1219883)
Supplement: Supplementary file 1 [file DataSheet1.PDF]

Supplementary Material

Development of the first Geldanamycin-Based HSP90 Degraders

Silas L. Wurnig,‡[1] Melina Vogt,‡[2] Julian Hogenkamp,[2] Niklas Dienstbier,[2] Arndt Borkhardt,[2] Sanil Bhatia*[2] and Finn K. Hansen*[1]

[1]Department of Pharmaceutical and Cell Biological Chemistry, Pharmaceutical Institute, University of Bonn, An der Immenburg 4, 53121 Bonn, Germany.

[2]Department of Pediatric Oncology, Hematology and Clinical Immunology, Medical Faculty, Heinrich Heine University Düsseldorf, Moorenstr. 5, 40225 Düsseldorf, Germany.

‡ These authors contributed equally

*** Correspondence:**
Corresponding Authors: Finn Hansen: finn.hansen@uni-bonn.de and Sanil Bhatia: sanil.bhatia@med.uni-duesseldorf.de

Table of contents

1 Supplementary Data S2
1.1 Synthesis of compounds 1a-1f, 2a-2h, 3a-3g, 4, 5, and nc-3a S2
1.2 HPLC chromatograms of compounds 3a-3g and nc-3a S13
1.3 ^{1}H-, ^{13}C- & ^{19}F-NMR spectra S17
2 Supplementary Figures S41

1 Supplementary Data

1.1 Synthesis of compounds 1a-1f, 2a-2h, 3a-3g, 4, 5, and nc-3a

1.1.1 *tert*-Butyl (2-(2-(2-aminoethoxy)ethoxy)ethyl)carbamate (1a)

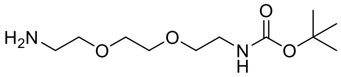

1a was synthesized according to General Procedure A using 2,2'-(ethylenedioxy)diethylamine (1.00 g, 985 µL, 6.68 mmol, 1.0 *eq.*) as starting material. The desired compound 1a was obtained as yellow highly viscous oil (709 mg, 2.70 mmol). Yield: 40%; R_f: 0.42 (CH_2Cl_2/MeOH (9/1) + 2% Et_3N (*v/v*)); LRMS-ESI (*m/z*): $[M+H]^+$ calcd for $C_{11}H_{25}N_2O_4^+$: 249.2, found: 249.2; ^1H NMR (600 MHz, $CDCl_3$): δ 5.20 (s, 1H), 3.56 (s, 4H), 3.52 – 3.48 (m, 4H), 3.28 – 3.22 (m, 2H), 2.83 (t, *J* = 5.2 Hz, 2H), 2.09 (s, 2H), 1.38 (s, 9H); ^{13}C NMR (151 MHz, $CDCl_3$) δ 156.0, 79.3, 79.2, 73.3, 70.2, 70.2, 41.7, 40.4, 28.4.

1.1.2 *tert*-Butyl (2-(2-(2-(2-aminoethoxy)ethoxy)ethoxy)ethyl)carbamate (1b)

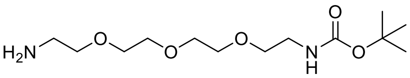

1b was synthesized according to General Procedure A using 1,11-diamino-3,6,9-trioxaundecane (1.00 g, 1.00 mL, 5.15 mmol, 1.0 *eq.*) as starting material. The desired compound 1b was obtained as a yellow highly viscous oil (587 mg, 2.02 mmol). Yield: 39%; R_f: 0.51 (DCM/MeOH (9/1) + 2% Et_3N (*v/v*)); LRMS-ESI (*m/z*): $[M+H]^+$ calcd for $C_{13}H_{29}N_2O_5^+$: 293.2, found: 293.2; ^1H NMR (600 MHz, $CDCl_3$) δ 5.28 (s, 1H), 3.65 – 3.56 (m, 8H), 3.54 – 3.48 (m, 4H), 3.28 (s, 2H), 2.87 (t, *J* = 5.1 Hz, 2H), 2.50 (s, 2H), 1.41 (s, 9H); ^{13}C NMR (151 MHz, $CDCl_3$) δ 156.2, 79.2, 70.6, 70.6, 70.4, 70.3, 46.1, 41.6, 40.4, 28.5.

1.1.3 *tert*-Butyl (3-(4-(3-aminopropoxy)butoxy)propyl)carbamate (1c)

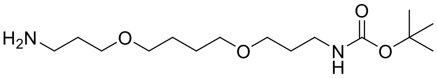

1c was synthesized according to General Procedure A using 4,9-dioxo-1,12-dodecanediamine (2.00 g, 2.08 mL, 9.69 mmol, 1.0 *eq.*) as starting material. The desired compound 1c was obtained as a yellow highly viscous oil (1.37 g, 4.49 mmol). Yield: 46%; R_f: 0.47 (CH_2Cl_2/MeOH (9/1) + 2% Et_3N (*v/v*)); LRMS-ESI (*m/z*): $[M+H]^+$ calcd for $C_{15}H_{33}N_2O_4^+$: 305.2, found: 305.4; ^1H NMR (600 MHz, $CDCl_3$) δ 4.96 (s, 1H), 3.50 – 3.36 (m, 8H), 3.22 – 3.17 (m, 2H), 2.81 (t, *J* = 6.7 Hz, 2H), 2.21 (s, 2H), 1.76 – 1.60 (m, 4H), 1.65 – 1.57 (m, 4H), 1.41 (s, 9H); ^{13}C NMR (151 MHz, $CDCl_3$) δ 156.0, 78.9, 70.8, 69.2, 69.0, 39.7, 38.8, 32.9, 29.7, 28.4, 26.5, 26.4.

1.1.4 *tert*-Butyl (3-(2-(2-(3-aminopropoxy)ethoxy)ethoxy)propyl)carbamate (1d)

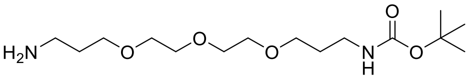

1d was synthesized according to General Procedure A using 4,7,10-trioxa-1,13-tridecanediamine (2.00 g, 1.98 mL, 8.90 mmol, 1.0 *eq.*) as starting material. The desired compound 1d was obtained as a yellow highly viscous oil (1.30 g, 4.06 mmol). Yield: 46%; R_f: 0.44 (CH_2Cl_2/MeOH (9/1) + 2% Et_3N (*v/v*)); LRMS-ESI (*m/z*): $[M+H]^+$ calcd for $C_{15}H_{33}N_2O_5^+$: 321.2, found: 321.4; ^1H NMR (600 MHz,

$CDCl_3$) δ 5.14 (s, 1H), 3.82 (s, 2H), 3.63 – 3.54 (m, 10H), 3.51 (t, *J* = 5.9 Hz, 2H), 3.22 – 3.15 (m, 2H), 2.88 (t, *J* = 6.5 Hz, 2H), 1.82 – 1.76 (m, 2H), 1.76 – 1.69 (m, 2H), 1.39 (s, 9H); ^{13}C NMR (151 MHz, $CDCl_3$) δ 156.2, 78.9, 70.5, 70.4, 70.1, 70.1, 69.6, 69.4, 39.6, 38.4, 31.2, 29.7, 28.5.

1.1.5 *tert*-Butyl (6-aminohexyl)carbamate (1e)

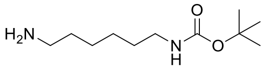

1e was synthesized according to General Procedure B using 1,6-diaminohexane (1.00 g, 8.52 mmol, 2.0 *eq.*) as starting material. The desired product 1e was obtained as an off-white wax (553 mg, 2.56 mmol). Yield: 60%; R_f: 0.41 (CH_2Cl_2/MeOH (9/1) + 2% Et_3N (*v/v*)); LRMS-ESI (*m/z*): $[M+H]^+$ calcd for $C_{11}H_{25}N_2O_2^+$: 217.2, found: 217.1; ^1H NMR (600 MHz, $CDCl_3$) δ 4.57 (s, 1H), 3.13 – 3.05 (m, 2H), 2.67 (t, *J* = 7.1 Hz, 2H), 1.96 (s, 2H), 1.48 – 1.42 (m, 13H), 1.36 – 1.26 (m, 4H); ^{13}C NMR (151 MHz, $CDCl_3$) δ 156.1, 79.1, 42.0, 40.6, 33.4, 30.1, 28.5, 26.7, 26.6.

1.1.6 *tert*-Butyl (8-aminooctyl)carbamate (1f)

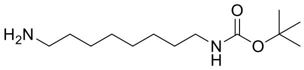

1f was synthesized according to General Procedure B using 1,8-diaminooctane (1.00 g, 6.86 mmol, 2.0 *eq.*) as starting material. The desired compound 1f was obtained as an off-white wax. (391 mg, 1.60 mmol). Yield: 47%; R_f: 0.61 (CH_2Cl_2/MeOH (9/1) + 2% Et_3N (*v/v*)); LRMS-ESI (*m/z*): $[M+H]^+$ calcd for $C_{13}H_{29}N_2O_2^+$: 245.2, found: 245.0; ^1H NMR (600 MHz, $CDCl_3$) δ 4.57 (s, 1H), 3.13 – 3.05 (m, 2H), 2.67 (t, *J* = 7.1 Hz, 2H), 1.96 (s, 2H), 1.50 – 1.38 (m, 16H), 1.36 – 1.26 (m, 5H); ^{13}C NMR (151 MHz, $CDCl_3$) δ 155.9, 78.9, 41.4, 40.5, 31.7, 29.9, 29.1, 29.1, 28.4, 26.6

1.1.7 *tert*-Butyl (2-(2-(2-((2-(2,6-dioxopiperidin-3-yl)-1,3-dioxoisoindolin-4-yl)amino)ethoxy)ethoxy)ethyl)carbamate (2a)

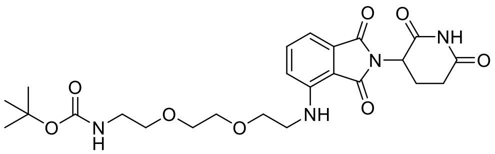

Compound 2a was synthesized according to General Procedure C using 1a (449 mg, 1.72 mmol, 1.0 *eq.*) as starting material. The desired compound 2a was obtained as a yellow oil (357 mg, 0.71 mmol). Yield: 41%; R_f: 0.14 (cyclohexane/ethyl acetate (1/1) (*v/v*)); LRMS-ESI (*m/z*): $[M+H]^+$ calcd for $C_{24}H_{33}N_4O_8^+$: 505.2, found: 505.2; ^1H NMR (600 MHz, $CDCl_3$): δ 8.51 (s, 1H), 7.48 (dd, *J* = 8.5, 7.1 Hz, 1H), 7.10 (d, *J* = 7.1 Hz, 1H), 6.90 (d, *J* = 8.5 Hz, 1H), 5.07 (s, 1H), 4.92 (s, 1H), 3.75 – 3.68 (m, 2H), 3.68 – 3.60 (m, 4H), 3.58 – 3.52 (m, 2H), 3.50 – 3.43 (m, 2H), 3.33 – 3.28 (m, 2H), 2.90 – 2.68 (m, 3H), 2.15 – 2.09 (m, 1H), 1.42 (s, 9H); ^{13}C NMR (151 MHz, $CDCl_3$): δ 171.2, 169.4, 168.5, 167.6, 156.1, 146.8, 136.1, 132.6, 116.7, 111.7, 110.4, 79.4, 70.8, 70.4, 70.2, 69.4, 48.9, 42.7, 42.3, 31.4, 28.4, 22.9.

1.1.8 *tert*-Butyl (2-(2-(2-(2-((2-(2,6-dioxopiperidin-3-yl)-1,3-dioxoisoindolin-4-yl)amino)ethoxy)ethoxy)ethoxy)ethyl)carbamate (2b)

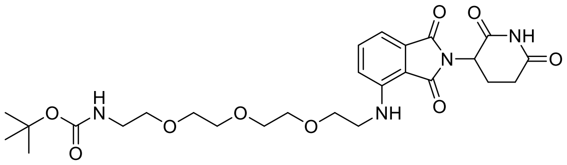

Compound 2b was synthesized according to General Procedure C using 1b (580 mg, 1.72 mmol, 1.0 *eq.*) as starting material. The desired compound 2b was obtained as a yellow oil (220 mg, 0.40 mmol). Yield: 29%; R_f: 0.65 (ethyl acetate) LRMS-ESI (*m/z*): $[M+H]^+$ calcd for $C_{26}H_{37}N_4O_9^+$: 549.3, found: 549.5; ^{1}H NMR (600 MHz, $CDCl_3$) δ 8.44 (s, 1H), 7.48 (dd, *J* = 8.5, 7.1 Hz, 1H), 7.09 (d, *J* = 7.1 Hz, 1H), 6.91 (d, *J* = 8.5 Hz, 1H), 5.09 (s, 1H), 4.94 – 4.88 (m, 1H), 3.72 (t, *J* = 5.4 Hz, 2H), 3.67 (s, 4H), 3.66 – 3.58 (m, 5H), 3.52 (t, *J* = 5.2 Hz, 2H), 3.47 (t, *J* = 5.4 Hz, 2H), 3.32 – 3.25 (m, 2H), 2.88 – 2.70 (m, 3H), 2.14 – 2.08 (m, 1H), 1.43 (s, 9H); ^{13}C NMR (151 MHz, $CDCl_3$) δ 171.2, 169.3, 168.5, 167.6, 156.1, 146.8, 136.1, 132.5, 116.8, 111.7, 110.3, 79.3, 70.8, 70.6, 70.6, 70.3, 70.2, 69.5, 60.4, 48.9, 42.4, 31.4, 28.4, 22.8.

1.1.9 *tert*-Butyl (3-(4-(3-((2-(2,6-dioxopiperidin-3-yl)-1,3-dioxoisoindolin-4-yl)amino)propoxy)butoxy)propyl)carbamate (2c)

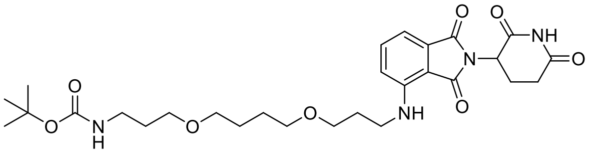

Compound 2c was synthesized according to General Procedure C using 1c (551 mg, 1.72 mmol, 1.0 *eq.*) as starting material. The desired compound 2c was obtained as a yellow oil (321 mg, 0.57 mmol). Yield: 33%; R_f: 0.56 (cyclohexane/ethyl acetate (3/1) (*v/v*)); LRMS-ESI (*m/z*): $[M+H]^+$ calcd for $C_{28}H_{41}N_4O_8^+$: 561.0, found: 561.0; ^{1}H NMR (600 MHz, $CDCl_3$) δ 8.35 (s, 1H), 7.48 (dd, *J* = 8.6, 7.1 Hz, 1H), 7.07 (d, *J* = 7.1 Hz, 1H), 6.91 (d, *J* = 8.5 Hz, 1H), 6.44 (s, 1H), 4.93 – 4.87 (m, 2H), 3.53 (t, *J* = 5.7 Hz, 2H), 3.49 – 3.35 (m, 8H), 3.23 – 3.17 (m, 2H), 2.91 – 2.85 (m, 1H), 2.83 – 2.68 (m, 2H), 2.15 – 2.08 (m, 1H), 1.94 – 1.87 (m, 2H), 1.77 – 1.70 (m, 2H), 1.70 – 1.58 (m, 4H), 1.43 (s, 9H). ^{13}C NMR (151 MHz, $CDCl_3$) δ 171.3, 169.5, 168.6, 167.8, 156.2, 147.1, 136.2, 132.6, 116.8, 111.5, 110.0, 79.2, 71.1, 70.9, 69.2, 68.4, 49.0, 40.4, 39.0, 31.5, 29.9, 29.5, 28.6, 26.6, 26.5, 22.9.

1.1.10 *tert*-Butyl (3-(2-(2-(3-((2-(2,6-dioxopiperidin-3-yl)-1,3-dioxoisoindolin-4-yl)amino)propoxy)ethoxy)ethoxy)propyl)carbamate (2d)

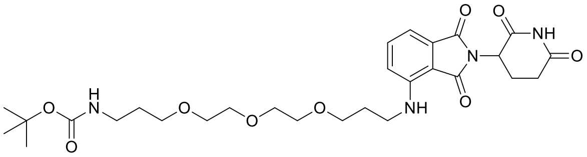

Compound 2d was synthesized according to General Procedure C using 1d (580 mg, 1.72 mmol, 1.0 *eq.*) as starting material. The desired compound 2d was obtained as a yellow oil (342 mg, 0.59 mmol). Yield: 35%; R_f: 0.63 (cyclohexane/ethyl acetate (3/1) (*v/v*)); LRMS-ESI (*m/z*): $[M+H]^+$ calcd for $C_{28}H_{41}N_4O_9^+$: 577.3, found: 577.2; ^{1}H NMR (600 MHz, $CDCl_3$) δ 8.56 (s, 1H), 7.47 (dd, *J* = 8.5, 7.1 Hz, 1H), 7.06 (d, *J* = 7.0 Hz, 1H), 6.91 (d, *J* = 8.5 Hz, 1H), 4.98 (s, 1H), 4.93 – 4.87 (m, 1H), 3.69 – 3.55 (m, 10H), 3.51 (t, *J* = 6.0 Hz, 2H), 3.39 (t, *J* = 6.6 Hz, 2H), 3.22 – 3.16 (m, 2H), 2.88 –

2.82 (m, 1H), 2.82 – 2.67 (m, 2H), 2.14 – 2.07 (m, 1H), 1.95 – 1.87 (m, 2H), 1.76 – 1.69 (m, 2H), 1.41 (s, 9H); ^{13}C NMR (151 MHz, $CDCl_3$) δ 171.3, 169.4, 168.6, 167.7, 156.1, 147.0, 136.1, 132.5, 116.7, 111.4, 109.9, 79.0, 70.6, 70.5, 70.2, 69.5, 68.8, 60.4, 48.9, 40.2, 31.4, 29.7, 29.3, 28.5, 22.8.

1.1.11 *tert*-Butyl (6-((2-(2,6-dioxopiperidin-3-yl)-1,3-dioxoisoindolin-4-yl)amino)hexyl)carbamate (2e)

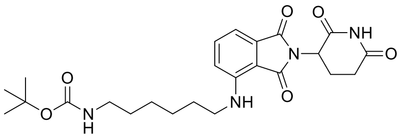

Compound 2e was synthesized according to General Procedure C using 1e (329 mg, 1.45 mmol, 1.0 *eq.*) as starting material. The desired compound 2e as a yellow oil (284 mg, 0.60 mmol). Yield: 42%; R_f: 0.42 (cyclohexane/ethyl acetate (1/1) (*v/v*)); LRMS-ESI (*m/z*): $[M+H]^+$ calcd for $C_{24}H_{33}N_4O_6^+$: 473.2, found: 473.2; ^{1}H NMR (500 MHz, DMSO-d_6) δ 11.06 (s, 1H), 7.57 (dd, J = 8.6, 7.1 Hz, 1H), 7.08 (d, J = 8.6 Hz, 1H), 7.01 (d, J = 7.1 Hz, 1H), 6.75 – 6.69 (m, 1H), 6.54 – 6.48 (m, 1H), 5.05 (dd, J = 12.7, 5.5 Hz, 1H), 3.28 – 3.24 (m, 1H), 2.94 – 2.87 (m, 3H), 2.62 – 2.54 (m, 1H), 2.07 – 1.98 (m, 1H), 1.61 – 1.52 (m, 2H), 1.43 – 1.23 (m, 17H); ^{13}C NMR (126 MHz, DMSO-d_6) δ 172.9, 170.2, 169.1, 167.4, 155.7, 146.6, 136.4, 132.3, 117.3, 110.5, 109.2, 77.4, 59.9, 48.7, 41.9, 31.1, 29.5, 28.8, 28.4, 26.1, 22.3, 14.2.

1.1.12 *tert*-Butyl (8-((2-(2,6-dioxopiperidin-3-yl)-1,3-dioxoisoindolin-4-yl)amino)octyl)carbamate (2f)

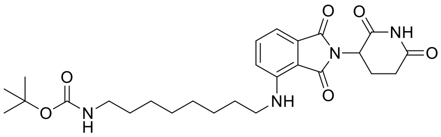

Compound 2f was synthesized according to General Procedure C using 1f (292 mg, 1.14 mmol, 1.0 *eq.*) as starting material. The desired compound 2f as a yellow oil (266 mg, 0.53 mmol). Yield: 47%; R_f: 0.56 (cyclohexane/ethyl acetate (1/1) (*v/v*)); LRMS-ESI (*m/z*): $[M+H]^+$ calcd for $C_{26}H_{37}N_4O_6^+$: 501.3, found: 501.3; ^{1}H NMR (500 MHz, DMSO-d_6) δ 11.06 (s, 1H), 7.57 (dd, J = 8.6, 7.1 Hz, 1H), 7.08 (d, J = 8.6 Hz, 1H), 7.01 (d, J = 7.0 Hz, 1H), 6.73 – 6.67 (m, 1H), 6.53 – 6.47 (m, 1H), 5.04 (dd, J = 12.7, 5.4 Hz, 1H), 3.28 – 3.25 (m, 1H), 2.94 – 2.82 (m, 3H), 2.63 – 2.53 (m, 2H), 2.08 – 1.97 (m, 1H), 1.60 – 1.52 (m, 2H), 1.41 – 1.16 (m, 20H); ^{13}C NMR (126 MHz, DMSO-d_6) δ 172.7, 170.0, 168.9, 167.2, 155.5, 146.4, 136.2, 132.2, 117.1, 110.3, 109.0, 77.2, 48.5, 41.8, 30.9, 29.4, 28.7, 28.6, 28.6, 28.2, 26.2, 26.2, 22.1, 13.9.

1.1.13 *tert*-Butyl (10-((2-(2,6-dioxopiperidin-3-yl)-1,3-dioxoisoindolin-4-yl)amino)decyl)carbamate (2g)

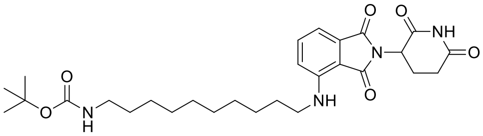

Compound 2g was synthesized according to General Procedure C using *tert*-butyl-(10-aminodecyl) carbamate (493 mg, 1.72 mmol, 1.0 *eq.*) as starting material. The desired compound 2g was obtained as a yellow oil (282 mg, 0.53 mmol). Yield: 31%; R_f: 0.60 (cyclohexane/ethyl acetate (1/1) (*v/v*)); LRMS-ESI (*m/z*): $[M+H]^+$ calcd for $C_{28}H_{10}N_4O_6^+$: 529.3, found: 529.2; ^{1}H NMR (500 MHz, DMSO-d_6) δ 11.05 (s, 1H), 7.56 (dd, J = 8.6, 7.1 Hz, 1H), 7.07 (d, J = 8.6 Hz, 1H), 7.00 (d, J = 7.1 Hz, 1H), 6.72 – 6.66 (m, 1H), 6.52 – 6.46 (m, 1H), 5.03 (dd, J = 12.7, 5.5 Hz, 1H), 3.30 – 3.22 (m, 3H), 2.94 –

2.82 (m, 3H), 2.62 – 2.51 (m, 2H), 2.07 – 1.96 (m, 1H), 1.61 – 1.51 (m, 2H), 1.38 – 1.12 (s, 23H); ^{13}C NMR (126 MHz, DMSO-d_6) δ 172.7, 170.0, 168.9, 167.2, 155.5, 146.4, 136.2, 132.2, 117.1, 110.3, 109.0, 77.2, 59.7, 48.5, 41.8, 30.9, 29.4, 28.9, 28.9, 28.7, 28.6, 28.2, 26.3, 26.2, 22.1, 14.0.

1.1.14 (4E,6Z,8S,9S,10E,12S,13R,14S,16R)-19-((2-(2-(2-((2-(2,6-dioxopiperidin-3-yl)-1,3-dioxoisoindolin-4-yl)amino)ethoxy)ethoxy)ethyl)amino)-13-hydroxy-8,14-dimethoxy-4,10,12,16-tetramethyl-3,20,22-trioxo-2-azabicyclo[16.3.1]docosa-1(21),4,6,10,18-pentaen-9-yl carbamate (3a)

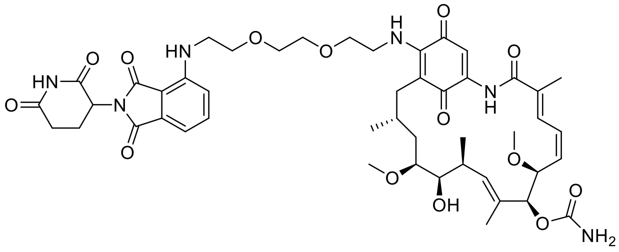

3a was synthesized according to General Procedure D using 2a as starting material (51 mg, 0.10 mmol, 2.0 *eq.*). The desired compound 3a was obtained as a red brown solid (25 mg, 0.03 mmol). Yield: 50%; mp: 122 – 129 °C; R_f: 0.43 (CH_2Cl_2/Acetone (3/1) (*v/v*)); HRMS-ESI (*m/z*): $[M+H]^+$ calcd for $C_{47}H_{61}N_6O_{14}^+$: 933.4240, found: 933.4240; ^1H NMR (600 MHz, Methanol-d_4: δ 7.55 – 7.48 (m, 1H), 7.15 – 7.06 (m, 1H), 7.06 – 7.01 (m, 1H), 7.01 – 6.97 (m, 1H), 6.95 – 6.88 (m, 1H), 6.65 – 6.57 (m, 1H), 5.90 – 5.82 (m, 1H), 5.66 – 5.59 (m, 1H), 5.23 – 5.14 (m, 1H), 5.03 – 4.93 (m, 1H), 4.55 – 4.48 (m, 1H), 3.82 – 3.64 (m, 10H), 3.61 – 3.56 (m, 1H), 3.50 – 3.41 (m, 3H), 3.35 – 3.32 (m, 3H), 3.28 (s, 3H), 2.89 – 2.79 (m, 1H), 2.77 – 2.64 (m, 4H), 2.37 – 2.27 (m, 1H), 2.14 – 2.06 (m, 1H), 2.00 – 1.96 (m, 3H), 1.80 – 1.70 (m, 4H), 1.70 – 1.53 (m, 2H), 1.00 – 0.93 (m, 6H); ^{13}C NMR (151 MHz, Methanol-d_4) δ 211.4, 185.6, 185.6, 181.1, 181.0, 174.6, 174.6, 171.4, 171.4, 170.6, 170.6, 169.2, 159.1, 159.1, 148.2, 148.2, 146.7, 146.7, 142.6, 139.3, 137.9, 137.2, 135.4, 135.2, 134.5, 134.5, 133.8, 133.7, 132.9, 132.7, 129.6, 127.3, 127.2, 125.5, 118.3, 112.1, 112.1, 111.3, 110.1, 110.0, 109.3, 109.2, 83.0, 82.1, 82.0, 74.2, 74.2, 71.7, 71.6, 71.6, 70.7, 70.7, 70.6, 70.2, 70.2, 57.5, 57.5, 56.9, 56.1, 50.2, 50.2, 49.6, 49.4, 49.3, 49.1, 49.0, 48.9, 48.7, 48.6, 46.2, 46.1, 43.3, 43.3, 35.8, 35.5, 34.4, 34.3, 33.9, 33.7, 33.1, 32.3, 32.3, 32.1, 31.9, 30.9, 30.8, 30.5, 29.5, 23.9, 23.9, 23.7, 22.7, 20.9, 13.6, 13.5, 12.4.

1.1.15 (4E,6Z,8S,9S,10E,12S,13R,14S,16R)-19-((2-(2-(2-(2-((2-(2,6-dioxopiperidin-3-yl)-1,3-dioxoisoindolin-4-yl)amino)ethoxy)ethoxy)ethoxy)ethyl)amino)-13-hydroxy-8,14-dimethoxy-4,10,12,16-tetramethyl-3,20,22-trioxo-2-azabicyclo[16.3.1]docosa-1(21),4,6,10,18-pentaen-9-yl carbamate (3b)

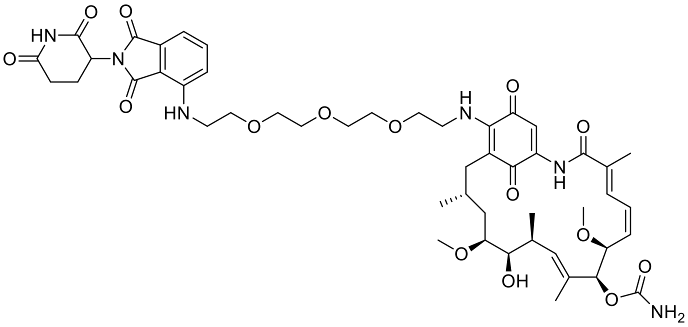

3b was synthesized according to General Procedure D using 2b as starting material (56 mg, 0.10 mmol, 2.0 *eq.*). The desired compound 3a was obtained as a red brown solid (28 mg, 0.03 mmol). Yield: 55%; mp: 117 – 119 °C; R_f: 0.65 (CH_2Cl_2/Acetone (1/1) (*v/v*)); HRMS-ESI (*m/z*): $[M+Na]^+$ calcd for

$C_{49}H_{64}N_6O_{15}Na^+$: 999.4322, found: 999.4322; ^{1}H NMR (600 MHz, Methanol-*d*$_4$) δ 7.54 – 7.48 (m, 1H), 7.14 – 7.07 (m, 1H), 7.07 – 6.96 (m, 3H), 6.62 (d, *J* = 11.4 Hz, 1H), 5.90 – 5.83 (m, 1H), 5.64 – 5.59 (m, 1H), 5.22 (s, 1H), 5.05 – 4.99 (m, 1H), 4.55 – 4.50 (m, 1H), 3.74 – 3.64 (m, 14H), 3.62 – 3.56 (m, 1H), 3.50 – 3.43 (m, 3H), 3.35 – 3.32 (m, 3H), 3.30 (s, 3H), 2.89 – 2.82 (m, 1H), 2.78 – 2.65 (m, 4H), 2.34 – 2.26 (m, 1H), 2.15 – 2.10 (m, 1H), 2.01 – 1.97 (m, 3H), 1.74 – 1.71 (m, 4H), 1.68 – 1.53 (m, 2H), 0.98 – 0.93 (m, 6H); ^{13}C NMR (151 MHz, Methanol-*d*$_4$) δ 211.7, 185.7, 181.0, 174.8, 171.5, 170.7, 170.6, 169.3, 159.1, 148.1, 146.8, 142.6, 137.9, 137.3, 135.3, 134.4, 133.8, 129.6, 127.2, 118.3, 118.3, 112.1, 111.2, 110.2, 109.2, 83.0, 81.9, 74.3, 71.7, 71.7, 71.7, 71.7, 71.6, 71.4, 71.4, 70.6, 70.6, 70.6, 70.2, 57.5, 56.8, 56.0, 50.2, 49.6, 49.4, 49.3, 49.1, 49.0, 48.9, 48.7, 48.6, 46.3, 43.3, 35.8, 34.4, 33.6, 33.0, 32.2, 32.1, 30.7, 30.4, 29.6, 29.5, 23.9, 23.8, 23.7, 22.7, 20.9, 13.6, 12.5.

1.1.16 (4E,6Z,8S,9S,10E,12S,13R,14S,16R)-19-((3-(4-(3-((2-(2,6-dioxopiperidin-3-yl)-1,3-dioxoisoindolin-4-yl)amino)propoxy)butoxy)propyl)amino)-13-hydroxy-8,14-dimethoxy-4,10,12,16-tetramethyl-3,20,22-trioxo-2-azabicyclo[16.3.1]docosa-1(21),4,6,10,18-pentaen-9-yl carbamate (3c)

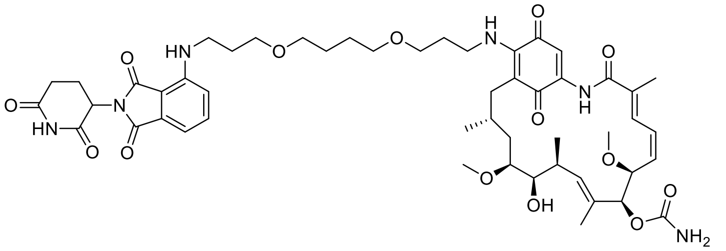

3c was synthesized according to General Procedure D using 2c as starting material (56 mg, 0.10 mmol, 2.0 *eq.*). The desired compound 3c was obtained as a red brown solid (40 mg, 0.04 mmol). Yield: 78%; mp: 145 – 150 °C; R_f: 0.42 (CH_2Cl_2/Acetone (3/1) (*v*/*v*)); HRMS-ESI (*m/z*): $[M+H]^+$ calcd for $C_{51}H_{69}N_6O_{14}^+$: 989.4833, found: 989.4866; ^{1}H NMR (600 MHz, Methanol-*d*$_4$) δ 7.53 – 7.46 (m, 1H), 7.11 – 7.05 (m, 1H), 7.04 – 6.96 (m, 3H), 6.65 – 6.57 (m, 1H), 5.89 – 5.83 (m, 1H), 5.67 – 5.59 (m, 1H), 5.23 – 5.19 (m, 1H), 5.04 – 4.97 (m, 1H), 4.54 – 4.49 (m, 1H), 3.71 – 3.53 (m, 8H), 3.50 – 3.44 (m, 5H), 3.43 – 3.38 (m, 2H), 3.33 (s, 3H), 3.30 – 3.27 (m, 3H), 2.90 – 2.80 (m, 1H), 2.79 – 2.63 (m, 4H), 2.39 – 2.31 (m, 1H), 2.15 – 2.07 (m, 1H), 1.98 (s, 3H), 1.94 – 1.86 (m, 4H), 1.75 – 1.63 (m, 8H), 1.62 – 1.55 (m, 1H), 0.99 – 0.94 (m, 6H); ^{13}C NMR (151 MHz, Methanol-*d*$_4$) δ 211.8, 185.7, 185.7, 180.8, 180.8, 174.9, 174.8, 174.8, 171.5, 171.4, 170.7, 170.6, 170.6, 169.4, 169.3, 159.1, 159.1, 148.3, 148.2, 146.8, 142.8, 137.9, 137.2, 137.2, 135.3, 134.4, 133.8, 129.6, 127.2, 118.0, 117.9, 111.8, 110.9, 110.9, 109.4, 109.1, 83.0, 82.0, 74.2, 72.2, 72.2, 72.0, 71.9, 70.6, 70.3, 70.3, 69.6, 69.6, 57.5, 56.9, 56.9, 56.0, 50.2, 50.1, 49.6, 49.4, 49.3, 49.1, 49.0, 48.9, 48.7, 48.6, 45.6, 45.6, 41.4, 41.3, 41.2, 35.9, 34.4, 33.8, 33.0, 32.2, 32.1, 31.4, 30.9, 30.7, 30.6, 30.5, 30.5, 30.4, 30.4, 29.6, 29.5, 27.6, 27.6, 27.4, 23.8, 23.7, 22.7, 20.9, 14.3, 13.6, 12.5.

1.1.17 (4E,6Z,8S,9S,10E,12S,13R,14S,16R)-19-((3-(2-(2-(3-((2-(2,6-dioxopiperidin-3-yl)-1,3-dioxoisoindolin-4-yl)amino)propoxy)ethoxy)ethoxy)propyl)amino)-13-hydroxy-8,14-dimethoxy-4,10,12,16-tetramethyl-3,20,22-trioxo-2-azabicyclo[16.3.1]docosa-1(21),4,6,10,18-pentaen-9-yl carbamate (3d)

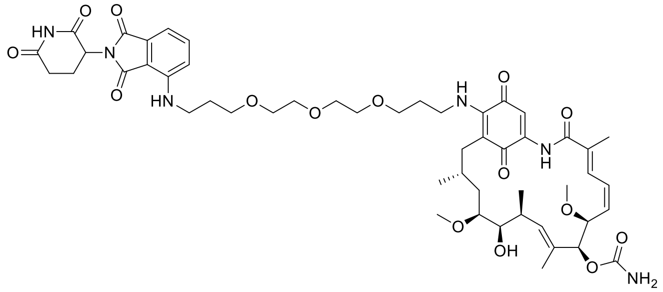

3d was synthesized according to General Procedure D using 2d as starting material (58 mg, 0.10 mmol, 2.0 *eq.*). The desired compound 3d was obtained as a red brown solid (41 mg, 0.04 mmol). Yield: 78%; mp: 123 – 132 °C; R_f: 0.77 (CH_2Cl_2/Acetone (1/1) (*v/v*)); HRMS-ESI (*m/z*): $[M+H]^+$ calcd for $C_{51}H_{69}N_6O_{15}^+$: 1005.4815, found: 1005.4815; ^{1}H NMR (600 MHz, Methanol-d_4) δ 7.54 – 7.47 (m, 1H), 7.11 – 7.04 (m, 1H), 7.04 – 7.00 (m, 1H), 7.00 – 6.95 (m, 2H), 6.65 – 6.58 (m, 1H), 5.89 – 5.83 (m, 1H), 5.67 – 5.59 (m, 1H), 5.25 – 5.20 (m, 1H), 5.04 – 4.97 (m, 1H), 4.56 – 4.49 (m, 1H), 3.74 – 3.67 (m, 4H), 3.67 – 3.54 (m, 10H), 3.48 – 3.43 (m, 1H), 3.43 – 3.37 (m, 2H), 3.34 (s, 3H), 3.29 – 3.28 (m, 3H), 2.89 – 2.81 (m, 1H), 2.79 – 2.63 (m, 4H), 2.38 – 2.29 (m, 1H), 2.16 – 2.09 (m, 2H), 2.01 – 1.97 (m, 3H), 1.94 – 1.86 (m, 4H), 1.73 (s, 4H), 1.69 – 1.54 (m, 2H), 0.99 – 0.93 (m, 6H); ^{13}C NMR (151 MHz, Methanol-d_4) δ 211.8, 210.5, 185.7, 185.7, 180.8, 174.9, 174.8, 171.5, 171.5, 170.7, 170.6, 170.6, 169.3, 169.3, 159.1, 159.1, 159.1, 159.1, 148.2, 148.2, 146.8, 146.7, 142.8, 137.9, 137.3, 135.3, 134.4, 133.8, 132.8, 129.6, 127.2, 127.2, 118.0, 111.8, 111.8, 110.9, 110.8, 109.5, 109.1, 83.0, 82.0, 74.2, 71.6, 71.6, 71.6, 71.5, 71.5, 71.4, 70.6, 70.5, 70.5, 70.0, 69.9, 57.5, 57.5, 56.9, 56.1, 50.2, 50.1, 49.4, 49.3, 49.1, 49.0, 48.9, 48.7, 48.6, 45.3, 45.2, 45.2, 41.3, 41.2, 41.1, 35.9, 34.4, 33.8, 33.0, 32.2, 32.1, 30.9, 30.7, 30.7, 30.6, 30.5, 30.5, 30.4, 30.4, 29.5, 23.9, 23.7, 22.7, 20.9, 14.3, 13.6, 12.5.

1.1.18 (4E,6Z,8S,9S,10E,12S,13R,14S,16R)-19-((6-((2-(2,6-dioxopiperidin-3-yl)-1,3-dioxoisoindolin-4-yl)amino)hexyl)amino)-13-hydroxy-8,14-dimethoxy-4,10,12,16-tetramethyl-3,20,22-trioxo-2-azabicyclo[16.3.1]docosa-1(21),4,6,10,18-pentaen-9-yl carbamate (3e)

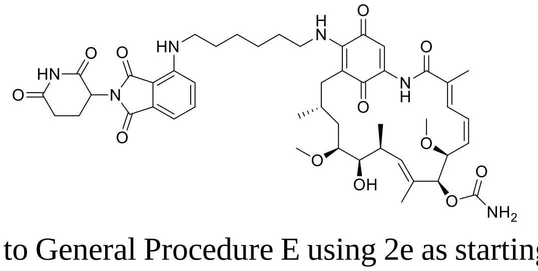

3e was synthesized according to General Procedure E using 2e as starting material (47 mg, 0.10 mmol, 2.0 *eq.*). The desired compound 3e was obtained as a red brown solid (24 mg, 0.03 mmol). Yield: 52%; mp: 158 – 162 °C; R_f: 0.64 (ethyl acetate); HRMS-ESI (*m/z*): $[M+Na]^+$ calcd for $C_{47}H_{60}N_6O_{12}Na^+$: 923.4167, found: 923.4161; ^{1}H NMR (600 MHz, Methanol-d_4) δ 7.56 – 7.50 (m, 1H), 7.14 – 7.09 (m, 1H), 7.05 – 6.99 (m, 3H), 6.62 (t, J = 11.7 Hz, 1H), 5.90 – 5.84 (m, 1H), 5.62 – 5.57 (m, 1H), 5.24 – 5.20 (m, 1H), 5.06 – 5.01 (m, 1H), 4.56 – 4.51 (m, 1H), 3.63 – 3.58 (m, 1H), 3.55 – 3.51 (m, 2H), 3.49 – 3.44 (m, 1H), 3.33 – 3.32 (m, 3H), 3.30 (s, 3H), 2.89 – 2.82 (m, 1H), 2.78 – 2.64 (m, 4H), 2.35 – 2.25 (m, 1H), 2.15 – 2.08 (m, 1H), 1.72 – 1.61 (m, 6H), 1.60 – 1.53 (m, 2H), 1.53 – 1.43 (m, 5H), 1.36

– 1.26 (m, 6H), 1.01 – 0.94 (m, 6H); ^{13}C NMR (151 MHz, Methanol-d_4) δ 185.8, 185.8, 180.9, 180.9, 174.9, 174.8, 171.6, 171.6, 170.8, 170.8, 169.4, 169.4, 159.1, 159.1, 148.4, 148.3, 146.6, 142.8, 137.9, 137.3, 135.4, 134.4, 133.8, 133.8, 130.9, 130.4, 129.6, 127.8, 127.2, 118.1, 111.9, 111.9, 110.9, 109.6, 109.1, 83.0, 81.9, 77.4, 74.3, 72.2, 67.4, 62.0, 57.5, 56.8, 50.2, 50.2, 49.4, 49.3, 49.1, 49.0, 48.9, 48.7, 48.6, 46.3, 46.2, 46.2, 43.3, 43.3, 43.2, 43.1, 38.9, 35.8, 35.3, 34.5, 33.7, 33.0, 32.8, 32.2, 32.2, 31.8, 30.8, 30.8, 30.7, 30.7, 30.7, 30.6, 30.4, 30.3, 30.2, 30.1, 30.0, 28.1, 27.5, 27.4, 27.3, 26.9, 26.1, 23.8, 23.8, 23.7, 22.9, 22.7, 21.0, 20.9, 20.3, 19.9, 14.3, 13.6, 12.5.

1.1.19 (4E,6Z,8S,9S,10E,12S,13R,14S,16R)-19-((8-((2-(2,6-dioxopiperidin-3-yl)-1,3-dioxoisoindolin-4-yl)amino)octyl)amino)-13-hydroxy-8,14-dimethoxy-4,10,12,16-tetramethyl-3,20,22-trioxo-2-azabicyclo[16.3.1]docosa-1(21),4,6,10,18-pentaen-9-yl carbamate (3f)

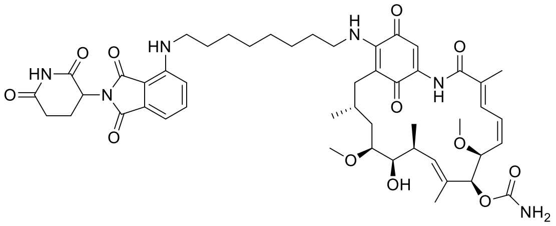

3f was synthesized according to General Procedure E using 2f as starting material (50 mg, 0.10 mmol, 2.0 *eq.*). The desired compound 3f was obtained as a red brown solid (26 mg, 0.03 mmol). Yield: 52%; mp: 155 – 162 °C; R_f: 0.28 (cyclohexane/ethyl acetate (1/3) (*v*/*v*)); HRMS-ESI (*m/z*): [M+Na]$^+$ calcd for $C_{49}H_{64}N_6O_{12}Na^+$: 951.4480, found: 951.4474; ^{1}H NMR (600 MHz, Methanol-d_4) δ 7.57 – 7.51 (m, 1H), 7.14 – 7.09 (m, 1H), 7.07 – 7.00 (m, 3H), 6.62 (t, *J* = 11.4 Hz, 1H), 5.90 – 5.84 (m, 1H), 5.62 – 5.57 (m, 1H), 5.22 (s, 1H), 5.07 – 5.01 (m, 1H), 4.56 – 4.52 (m, 1H), 3.64 – 3.57 (m, 1H), 3.56 – 3.50 (m, 2H), 3.50 – 3.44 (m, 1H), 3.34 (s, 3H), 3.30 (s, 3H), 2.88 – 2.80 (m, 1H), 2.79 – 2.65 (m, 4H), 2.35 – 2.25 (m, 1H), 2.15 – 2.08 (m, 1H), 1.99 (s, 3H), 1.83 – 1.77 (m, 1H), 1.73 (s, 3H), 1.70 – 1.61 (m, 5H), 1.61 – 1.52 (m, 2H), 1.46 – 1.36 (m, 9H), 0.99 – 0.96 (m, 6H); ^{13}C NMR (151 MHz, Methanol-d_4) δ 185.8, 180.9, 174.8, 171.6, 170.8, 169.4, 159.1, 159.1, 148.4, 148.3, 146.6, 142.9, 137.9, 137.3, 135.3, 134.4, 133.8, 133.8, 132.5, 130.4, 129.7, 127.8, 127.2, 118.0, 115.8, 111.8, 110.9, 109.5, 109.1, 83.0, 81.9, 77.4, 74.3, 72.2, 67.4, 57.6, 56.8, 52.5, 50.2, 49.6, 49.4, 49.3, 49.1, 49.0, 48.9, 48.7, 48.6, 46.3, 46.3, 43.5, 43.4, 35.8, 34.5, 33.6, 33.1, 32.8, 32.2, 31.7, 30.8, 30.8, 30.7, 30.6, 30.4, 30.3, 30.2, 30.1, 30.1, 30.1, 30.1, 30.0, 29.1, 27.7, 27.7, 27.5, 27.5, 23.8, 23.7, 23.7, 23.0, 22.9, 22.7, 20.9, 20.3, 19.9, 19.3, 14.3, 13.7, 12.4.

1.1.20 (4E,6Z,8S,9S,10E,12S,13R,14S,16R)-19-((10-((2-(2,6-dioxopiperidin-3-yl)-1,3-dioxoisoindolin-4-yl)amino)decyl)amino)-13-hydroxy-8,14-dimethoxy-4,10,12,16-tetramethyl-3,20,22-trioxo-2-azabicyclo[16.3.1]docosa-1(21),4,6,10,18-pentaen-9-yl carbamate (3g)

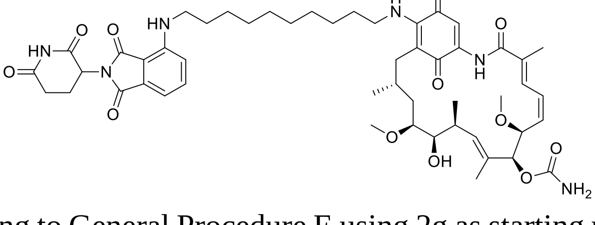

3g was synthesized according to General Procedure E using 2g as starting material (53 mg, 0.10 mmol, 2.0 *eq.*). The desired compound 3g was obtained as a red brown solid (22 mg, 0.02 mmol). Yield: 44%; mp: 165 – 169 °C; R_f: 0.22 (cyclohexane/ethylacetate (1/3) (*v*/*v*)); HRMS-ESI (*m/z*): [M+Na]$^+$ calcd

for $C_{51}H_{68}N_6O_{12}Na^+$: 979.4793, found: 979.4787; ^{1}H NMR (600 MHz, Methanol-d_4) δ 7.57 – 7.51 (m, 1H), 7.15 – 7.09 (m, 1H), 7.07 – 7.00 (m, 3H), 6.65 – 6.58 (m, 1H), 5.90 – 5.84 (m, 1H), 5.62 – 5.56 (m, 1H), 5.22 (s, 1H), 5.06 – 5.01 (m, 1H), 4.56 – 4.52 (m, 1H), 3.64 – 3.57 (m, 1H), 3.55 – 3.44 (m, 3H), 3.34 (s, 3H), 3.30 (s, 3H), 2.90 – 2.83 (m, 1H), 2.79 – 2.66 (m, 4H), 2.35 – 2.25 (m, 1H), 2.15 – 2.07 (m, 1H), 1.99 (s, 3H), 1.86 – 1.77 (m, 1H), 1.73 (d, J = 1.3 Hz, 3H), 1.70 – 1.61 (m, 5H), 1.60 – 1.52 (m, 2H), 1.47 – 1.34 (m, 13H), 1.03 – 0.95 (m, 6H); ^{13}C NMR (151 MHz, Methanol-d_4) δ 185.8, 180.9, 174.8, 171.6, 170.8, 169.4, 159.1, 159.1, 148.4, 148.3, 146.6, 142.9, 137.9, 137.3, 135.3, 134.4, 133.9, 132.4, 129.7, 127.2, 118.0, 111.8, 110.9, 109.5, 109.1, 83.0, 81.9, 77.4, 74.4, 72.2, 67.4, 62.0, 57.6, 56.8, 50.2, 49.6, 49.4, 49.3, 49.1, 49.0, 48.9, 48.7, 48.6, 46.5, 46.3, 43.5, 43.4, 38.9, 35.8, 34.6, 33.6, 33.1, 32.8, 32.2, 32.2, 31.4, 30.8, 30.8, 30.7, 30.4, 30.3, 30.3, 30.2, 30.2, 30.1, 27.8, 27.6, 26.9, 23.8, 23.7, 22.9, 22.7, 20.9, 20.3, 19.9, 14.3, 13.7, 12.4.

1.1.21 *tert*-Butyl (1-methyl-2,6-dioxopiperidin-3-yl)carbamate (4)

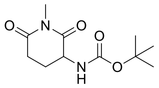

tert-Butyl-(2,6-dioxopiperidin-3-yl) carbamate (2.00 g, 8.76 mmol, 1.0 *eq.*) was dissolved in DMF (10 mL). Potassium carbonate (2.47 g, 17.52 mmol, 2.0 *eq.*) and methyl iodide (1.27 g, 8.76 mmol, 1.0 *eq.*) were added. The reaction mixture was heated to 50 °C for 4 h. After dilution with ethyl acetate (50 mL), the red mixture was washed with 1 N NaOH (2 x 25 mL), H_2O (25 mL) and brine (25 mL). The colorless solution was dried over Na_2SO_4, filtered, and evaporated. The residue was purified by column chromatography using a mixture of petroleum ether and ethyl acetate (2/1, *v/v*) to obtain compound 4 as a white gum (940 mg, 3.88 mmol). Yield: 44%; R_f: 0.65 (petroleum ether/ethyl acetate (2/1) (*v/v*)); LRMS-ESI (*m/z*): $[M+H]^+$ calcd for $C_{11}H_{19}N_2O_4^+$: 243.1, found: 243.2; ^{1}H NMR (600 MHz, DMSO-d_6) δ 7.17 (d, J = 8.8 Hz, 1H), 4.34 – 4.26 (m, 1H), 2.97 (s, 3H), 2.84 – 2.75 (m, 1H), 2.64 (ddd, J = 17.3, 4.5, 3.1 Hz, 1H), 1.98 – 1.86 (m, 2H), 1.40 (s, 9H); ^{13}C NMR (151 MHz, DMSO-d_6) δ 172.3, 172.0, 155.4, 78.2, 50.9, 31.1, 28.2, 26.4, 23.5.

1.1.22 4-Fluoro-2-(1-methyl-2,6-dioxopiperidin-3-yl)isoindoline-1,3-dione (5)

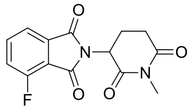

4-Fluoroisobenzofuran-1,3-dione (956 mg, 5.64 mmol, 1.5 *eq.*) was suspended in glacial acetic acid (30 mL). Compound 4 (930 mg, 3.76 mmol, 1.0 *eq.*) and sodium acetate (252 mg, 3.01 mmol, 0.8 *eq.*) were added and the mixture was heated under reflux overnight. After cooling the reaction mixture was poured onto ice-cold water and extracted with ethyl acetate (3 x 50 mL). The organic phase was dried over Na_2SO_4, filtered, and the solvent was evaporated under reduced pressure. The crude product was purified by column chromatography using CH_2Cl_2 as eluent to obtain compound 5 as a purple gum (672 mg, 2.32 mmol). Yield: 62%; R_f: 0.41 (CH_2Cl_2); LRMS-ESI (*m/z*): $[M+H]^+$ calcd for $C_{14}H_{12}FN_2O_4^+$: 291.1, found: 291.1; ^{1}H NMR (600 MHz, DMSO-d_6) δ 7.98 – 7.92 (m, 1H), 7.79 (d, J = 7.3 Hz, 1H), 7.74 (t, J = 8.8 Hz, 1H), 5.22 (dd, J = 13.1, 5.4 Hz, 1H), 3.03 (s, 3H), 3.00 – 2.91 (m, 1H), 2.78 (ddd, J = 17.3, 4.5, 2.5 Hz, 1H), 2.58 – 2.51 (m, 1H), 2.12 – 2.05 (m, 1H); ^{13}C NMR (151 MHz, DMSO-d_6) δ 171.7, 169.4, 166.1, 166.0, 163.9, 157.7, 155.9, 138.1, 138.0, 133.4, 123.1, 122.9, 120.0, 120.0, 117.0, 117.0, 49.7, 31.0, 26.6, 21.0; ^{19}F NMR (565 MHz, DMSO-d_6) δ -115.5.

1.1.23 *tert*-Butyl (2-(2-(2-((2-(1-methyl-2,6-dioxopiperidin-3-yl)-1,3-dioxoisoindolin-4-yl)amino)ethoxy)ethoxy)ethyl)carbamate (2h)

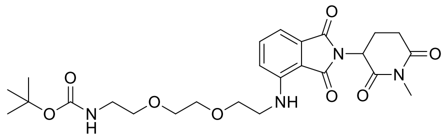

2h was synthesized according to general procedure B using 1a (128 mg, 0.49 mmol, 1.0 *eq.*) and 5 (150 mg, 0.49 mmol, 1.0 *eq.*) as starting materials. The desired compound 2h was obtained as a yellow oil (90 mg, 0.17 mmol). Yield: 35%; R_f: 0.51 (cyclohexane/ethyl acetate (1/3) (*v*/*v*)); LRMS-ESI (*m/z*): $[M+H]^+$ calcd for $C_{25}H_{35}N_4O_8^+$: 519.2, found: 519.2; ^1H NMR (500 MHz, DMSO-d_6) δ 7.59 (dd, *J* = 8.6, 7.1 Hz, 1H), 7.14 (d, *J* = 8.6 Hz, 1H), 7.04 (d, *J* = 7.0 Hz, 1H), 6.69 (t, *J* = 5.8 Hz, 1H), 6.60 (t, *J* = 5.8 Hz, 1H), 5.13 (dd, *J* = 13.0, 5.4 Hz, 1H), 3.62 (t, *J* = 5.5 Hz, 2H), 3.58 – 3.42 (m, 6H), 3.38 (t, *J* = 6.1 Hz, 2H), 3.06 (q, *J* = 6.0 Hz, 2H), 3.02 (s, 3H), 3.01 – 2.89 (m, 1H), 2.76 (ddd, *J* = 17.2, 4.5, 2.6 Hz, 1H), 2.56 (td, *J* = 13.3, 4.5 Hz, 1H), 2.09 – 2.00 (m, 1H), 1.37 (s, 9H); ^{13}C NMR (126 MHz, DMSO-d_6) δ 171.7, 169.7, 168.9, 167.2, 155.5, 146.4, 136.2, 132.0, 117.4, 110.6, 109.2, 77.5, 69.6, 69.5, 69.2, 68.8, 49.1, 42.1, 41.7, 31.1, 28.1, 26.5, 21.3.

1.1.24 (4E,6Z,8S,9S,10E,12S,13R,14S,16R)-13-hydroxy-8,14-dimethoxy-4,10,12,16-tetramethyl-19-((2-(2-(2-((2-(1-methyl-2,6-dioxopiperidin-3-yl)-1,3-dioxoisoindolin-4-yl)amino)ethoxy)ethoxy)ethyl)amino)-3,20,22-trioxo-2-azabicyclo[16.3.1]docosa-1(21),4,6,10,18-pentaen-9-yl carbamate (nc-3a)

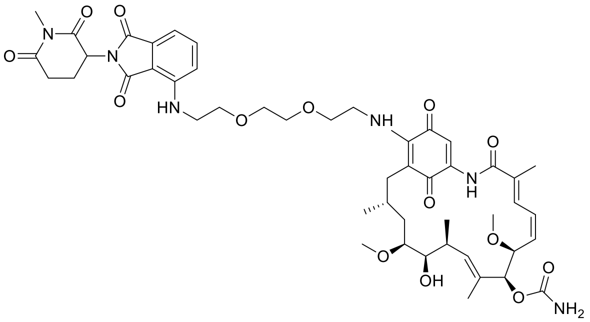

nc-3a was synthesized according to General Procedure D using 6 as starting material (52 mg, 0.10 mmol, 2.0 *eq.*). The desired compound 7 was obtained as a red brown solid (10 mg, 0.01 mmol). Yield: 20%; mp: 123 – 130 °C; R_f: 0.28 (cyclohexane/ethyl acetate (9/1) (*v*/*v*)); HRMS-ESI (*m/z*): $[M+Na]^+$ calcd for $C_{48}H_{62}N_6O_{14}Na^+$: 969.4222, found: 969.4216; ^1H NMR (600 MHz, Methanol-d_4) δ 7.55 – 7.49 (m, 1H), 7.13 – 7.08 (m, 1H), 7.08 – 7.03 (m, 1H), 7.03 – 6.98 (m, 1H), 6.98 – 6.93 (m, 1H), 6.62 (t, *J* = 11.4 Hz, 1H), 5.90 – 5.84 (m, 1H), 5.63 – 5.58 (m, 1H), 5.18 (s, 1H), 5.07 – 5.01 (m, 1H), 4.55 – 4.47 (m, 1H), 3.80 – 3.75 (m, 2H), 3.74 – 3.66 (m, 8H), 3.62 – 3.52 (m, 1H), 3.51 – 3.46 (m, 2H), 3.46 – 3.41 (m, 1H), 3.35 (s, 1H), 3.34 – 3.32 (m, 3H), 3.30 – 3.28 (m, 2H), 3.12 (s, 3H), 2.90 – 2.83 (m, 2H), 2.74 – 2.64 (m, 3H), 2.35 – 2.28 (m, 1H), 2.14 – 2.06 (m, 1H), 2.00 (s, 3H), 1.82 – 1.75 (m, 1H), 1.73 (s, 3H), 1.66 – 1.61 (m, 1H), 1.59 – 1.52 (m, 1H), 1.01 – 0.94 (m, 6H); ^{13}C NMR (151 MHz, Methanol-d_4) δ 211.4, 185.7, 185.6, 181.0, 181.0, 173.7, 171.3, 170.7, 170.7, 170.6, 169.3, 169.3, 159.1, 148.2, 146.8, 142.6, 137.9, 137.2, 135.4, 134.5, 133.8, 133.8, 132.6, 130.9, 129.6, 127.2, 120.6, 118.3, 112.1, 111.3, 110.1, 109.2, 82.9, 82.0, 74.3, 73.4, 71.7, 71.7, 71.7, 71.4, 70.7, 70.6, 70.3, 70.3, 69.8, 69.8, 62.2, 57.5, 57.5, 56.8, 56.8, 56.8, 56.1, 50.9, 50.9, 49.8, 49.6, 49.4, 49.3, 49.1, 49.0, 48.9, 48.7, 48.6, 46.2, 46.2, 43.3, 43.3, 40.8, 40.7, 36.5, 35.7, 34.5, 33.6, 33.1, 32.6, 32.5, 32.1, 31.4,

30.8, 30.8, 30.6, 30.6, 30.5, 30.3, 29.5, 28.1, 27.4, 26.9, 25.6, 23.7, 23.1, 23.1, 22.7, 16.8, 14.3, 13.6, 13.6, 12.4.

1.2 HPLC chromatograms of compounds 3a-3g and nc-3a

HPLC chromatogram of **3a**

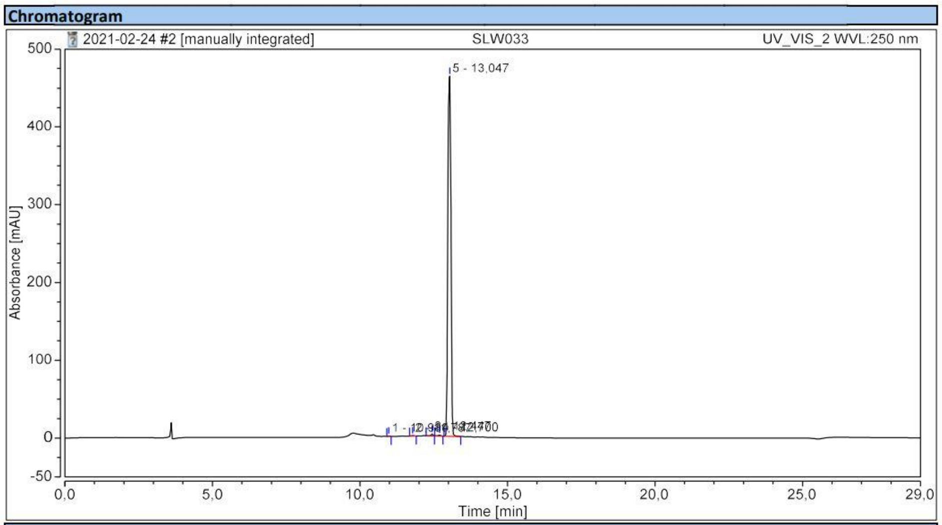

Integration Results

No.	Peak Name	Retention Time min	Area mAU*min	Height mAU	Relative Area %	Relative Height %	Amount n.a.
1		10,980	0,058	0,975	0,10	0,21	n.a.
2		11,787	0,071	0,617	0,13	0,13	n.a.
3		12,447	0,200	2,608	0,35	0,56	n.a.
4		12,700	0,131	1,221	0,23	0,26	n.a.
5		13,047	56,289	462,809	99,19	98,84	n.a.
**Total:**			**56,748**	**468,229**	**100,00**	**100,00**	

HPLC chromatogram of **3b**

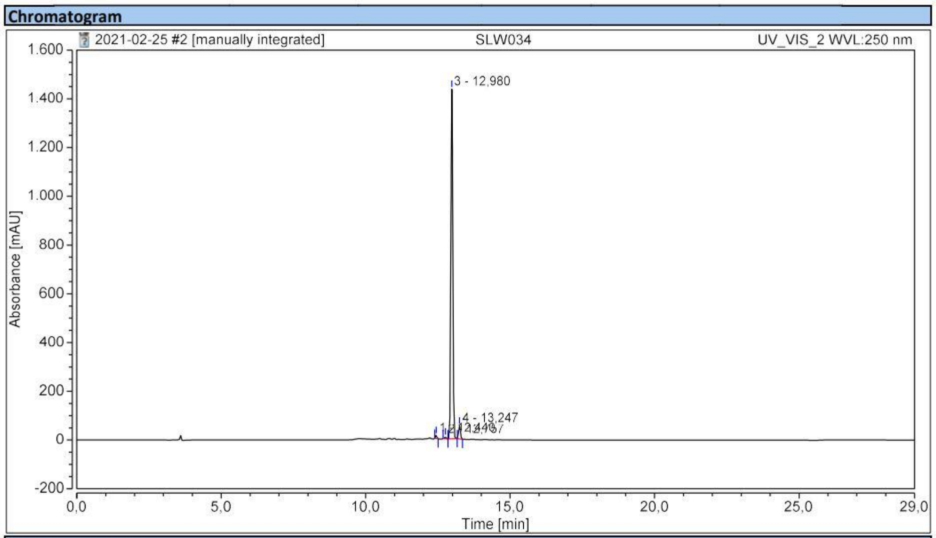

Integration Results

No.	Peak Name	Retention Time min	Area mAU*min	Height mAU	Relative Area %	Relative Height %	Amount n.a.
1		12,440	0,762	12,776	0,67	0,85	n.a.
2		12,757	0,536	7,609	0,47	0,50	n.a.
3		12,980	108,085	1434,308	95,56	95,11	n.a.
4		13,247	3,718	53,321	3,29	3,54	n.a.
**Total:**			**113,101**	**1508,013**	**100,00**	**100,00**	

HPLC chromatogram of **3c** (mixture of two separated diastereomers)

Chromatogram

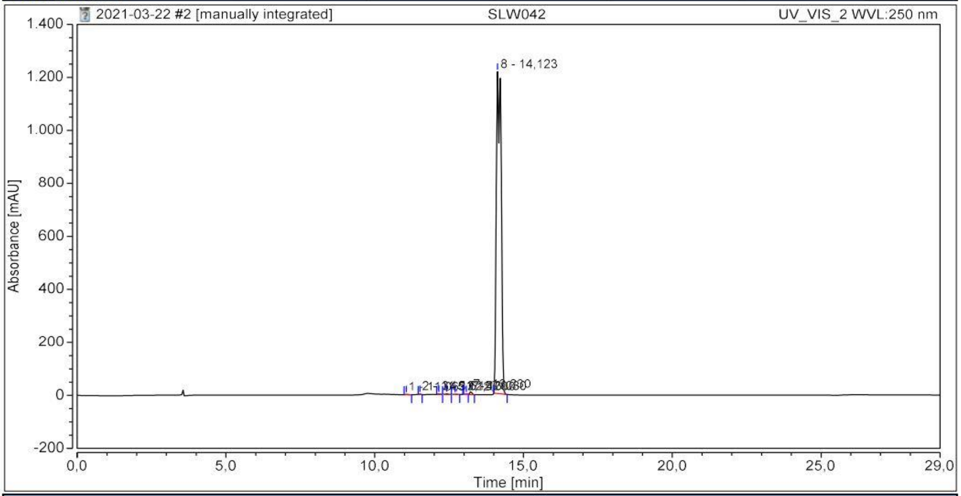

Integration Results

No.	Peak Name	Retention Time<br>min	Area<br>mAU*min	Height<br>mAU	Relative Area<br>%	Relative Height<br>%	Amount<br>n.a.
1		11,063	0,125	1,592	0,05	0,13	n.a.
2		11,517	0,109	1,906	0,05	0,15	n.a.
3		12,157	0,182	2,002	0,08	0,16	n.a.
4		12,420	0,274	2,500	0,12	0,20	n.a.
5		12,700	0,141	0,943	0,06	0,08	n.a.
6		13,080	0,120	1,588	0,05	0,13	n.a.
7		13,230	1,010	11,788	0,44	0,95	n.a.
8		14,123	228,218	1213,925	99,15	98,19	n.a.
**Total:**			**230,179**	**1236,243**	**100,00**	**100,00**	

HPLC chromatogram of **3d**

Chromatogram

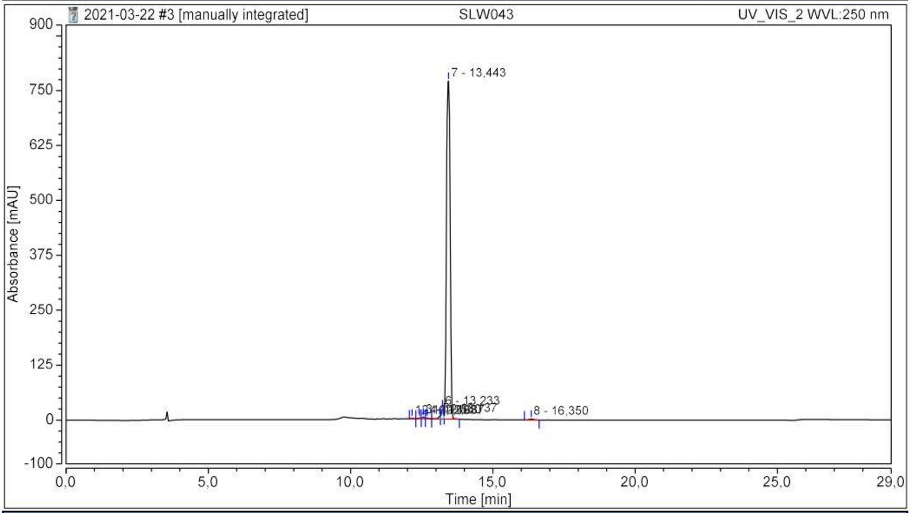

Integration Results

No.	Peak Name	Retention Time<br>min	Area<br>mAU*min	Height<br>mAU	Relative Area<br>%	Relative Height<br>%	Amount<br>n.a.
1		12,160	0,173	1,862	0,15	0,23	n.a.
2		12,423	0,234	2,219	0,20	0,27	n.a.
3		12,560	0,372	3,976	0,32	0,49	n.a.
4		12,687	0,216	1,934	0,19	0,24	n.a.
5		13,137	0,482	6,340	0,42	0,78	n.a.
6		13,233	1,924	23,282	1,66	2,87	n.a.
7		13,443	111,769	769,939	96,70	94,84	n.a.
8		16,350	0,412	2,305	0,36	0,28	n.a.
**Total:**			**115,583**	**811,857**	**100,00**	**100,00**	

HPLC chromatogram of **3e**

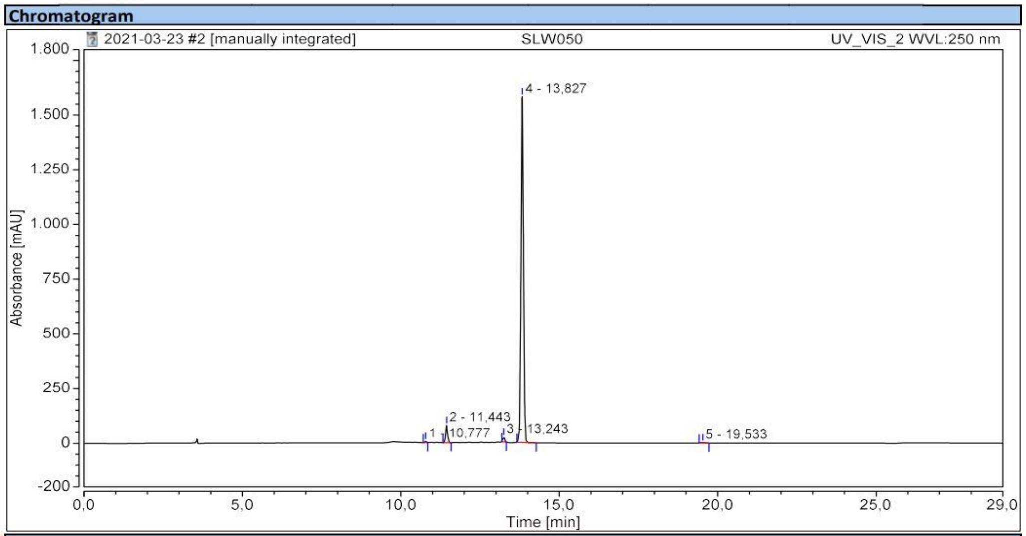

Integration Results

No.	Peak Name	Retention Time min	Area mAU*min	Height mAU	Relative Area %	Relative Height %	Amount n.a.
1		10,777	0,403	6,901	0,26	0,41	n.a.
2		11,443	5,496	78,629	3,52	4,66	n.a.
3		13,243	1,478	21,547	0,95	1,28	n.a.
4		13,827	148,591	1580,151	95,08	93,56	n.a.
5		19,533	0,308	1,730	0,20	0,10	n.a.
**Total:**			**156,276**	**1688,957**	**100,00**	**100,00**	

HPLC chromatogram of **3f**

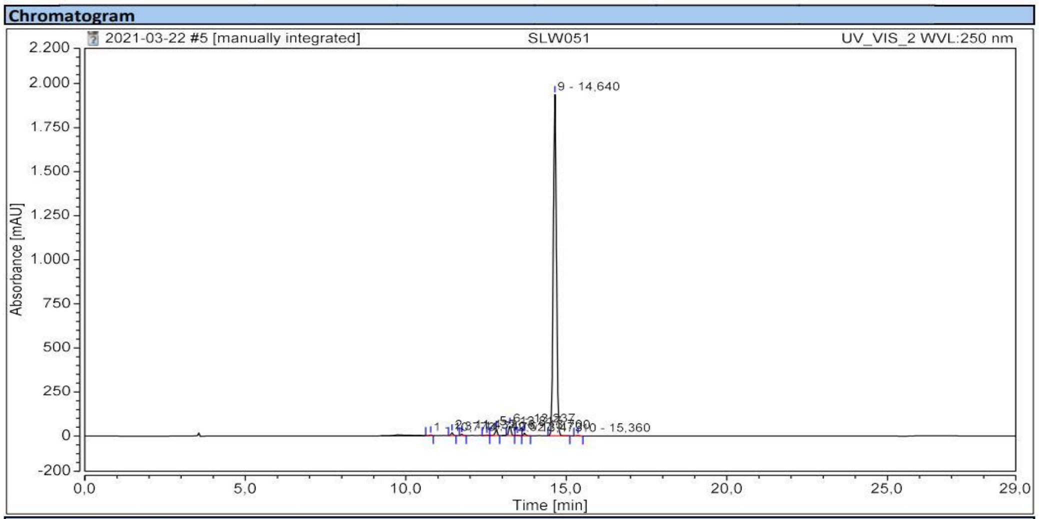

Integration Results

No.	Peak Name	Retention Time min	Area mAU*min	Height mAU	Relative Area %	Relative Height %	Amount n.a.
1		10,773	0,385	4,826	0,17	0,23	n.a.
2		11,437	1,171	16,528	0,51	0,79	n.a.
3		11,740	0,554	8,327	0,24	0,40	n.a.
4		12,527	0,275	3,450	0,12	0,17	n.a.
5		12,817	2,561	36,757	1,12	1,76	n.a.
6		13,237	4,290	54,318	1,88	2,61	n.a.
7		13,473	0,267	2,876	0,12	0,14	n.a.
8		13,700	1,090	16,392	0,48	0,79	n.a.
9		14,640	217,105	1937,337	95,26	93,02	n.a.
10		15,360	0,212	1,942	0,09	0,09	n.a.
**Total:**			**227,911**	**2082,753**	**100,00**	**100,00**	

HPLC chromatogram of **3g**

Chromatogram

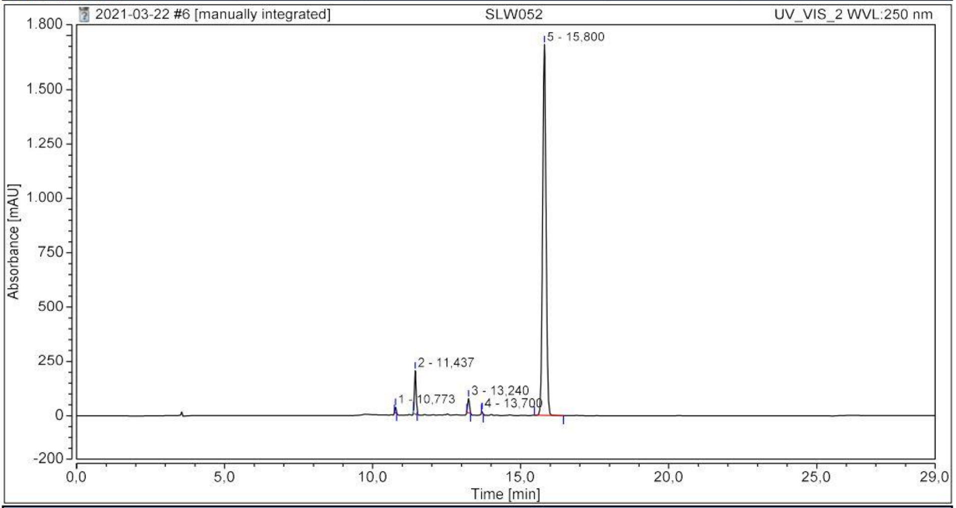

Integration Results

No.	Peak Name	Retention Time min	Area mAU*min	Height mAU	Relative Area %	Relative Height %	Amount n.a.
1		10,773	1,158	25,498	0,46	1,27	n.a.
2		11,437	13,296	198,794	5,29	9,92	n.a.
3		13,240	4,364	65,309	1,74	3,26	n.a.
4		13,700	0,324	8,489	0,13	0,42	n.a.
5		15,800	232,152	1706,257	92,38	85,13	n.a.
**Total:**			**251,294**	**2004,347**	**100,00**	**100,00**	

HPLC chromatogram of **nc-3a**

Chromatogram

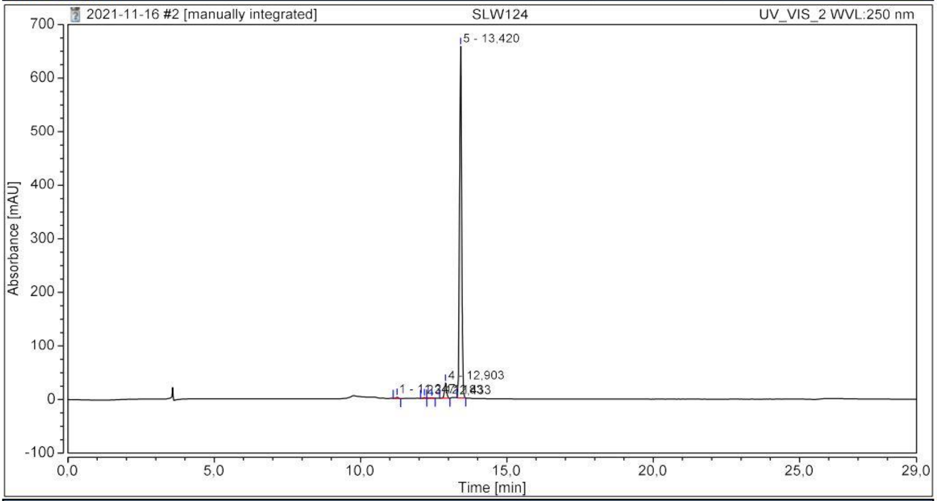

Integration Results

No.	Peak Name	Retention Time min	Area mAU*min	Height mAU	Relative Area %	Relative Height %	Amount n.a.
1		11,247	0,196	2,573	0,35	0,37	n.a.
2		12,183	0,124	1,635	0,22	0,24	n.a.
3		12,433	0,109	0,764	0,19	0,11	n.a.
4		12,903	2,181	28,020	3,87	4,06	n.a.
5		13,420	53,673	656,518	95,36	95,22	n.a.
**Total:**			**56,283**	**689,510**	**100,00**	**100,00**	

1.3 ^{1}H-, ^{13}C- & ^{19}F-NMR spectra

^{1}H-NMR spectrum of **1a**

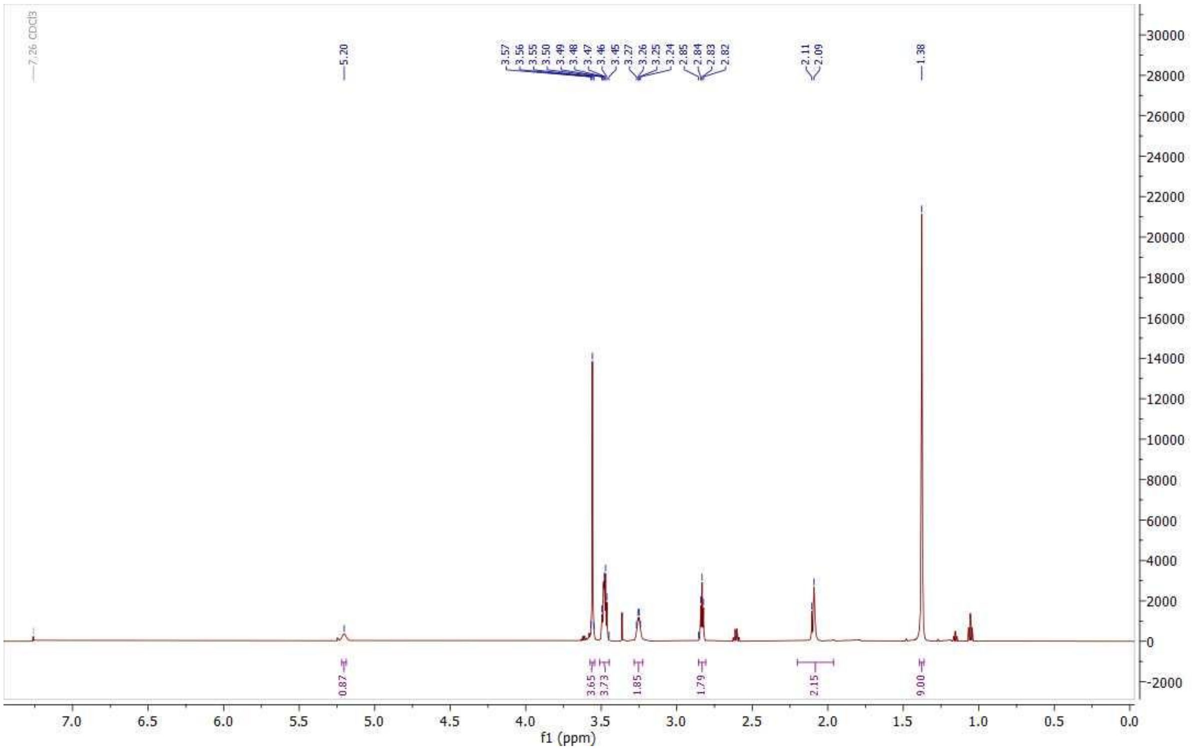

^{13}C-NMR spectrum of **1a**

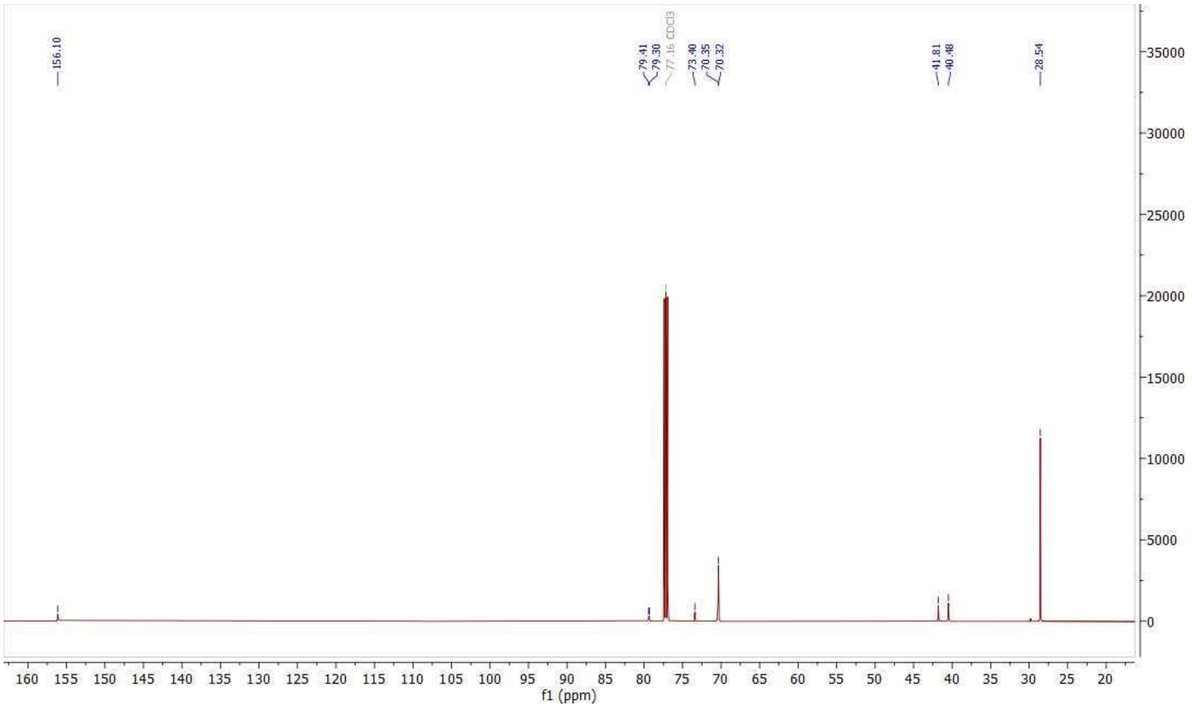

^{1}H-NMR spectrum of **1b**

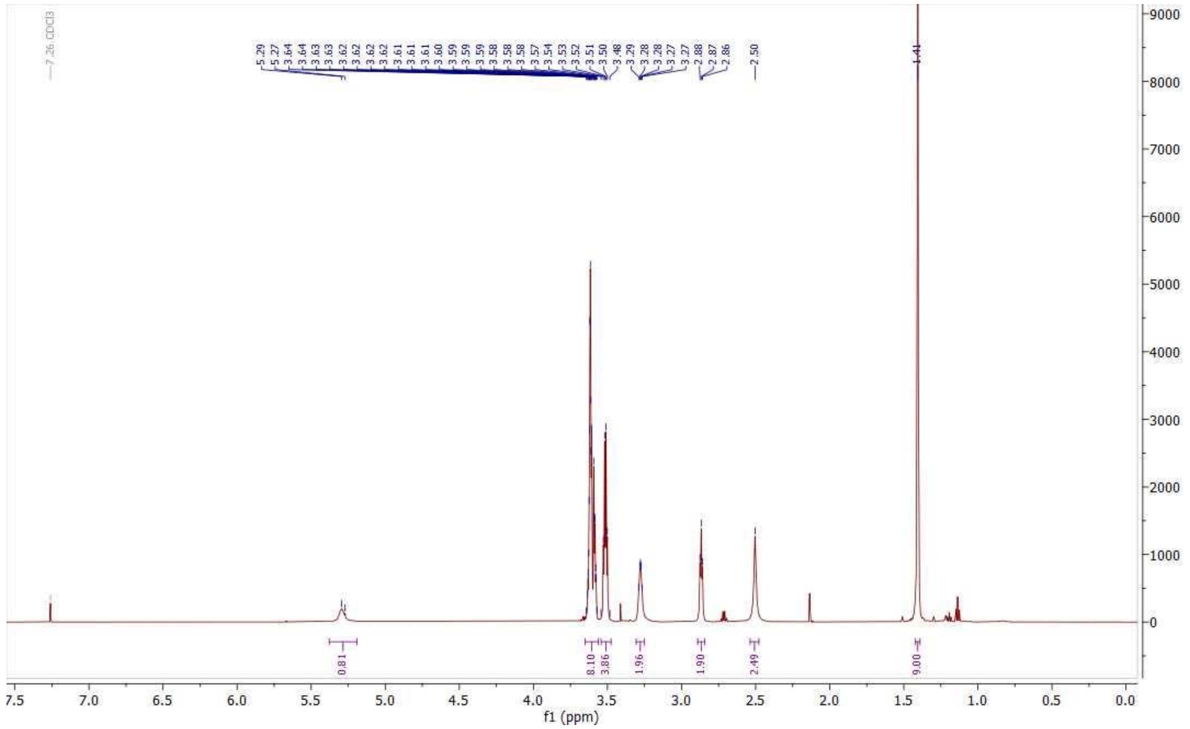

^{13}C-NMR spectrum of **1b**

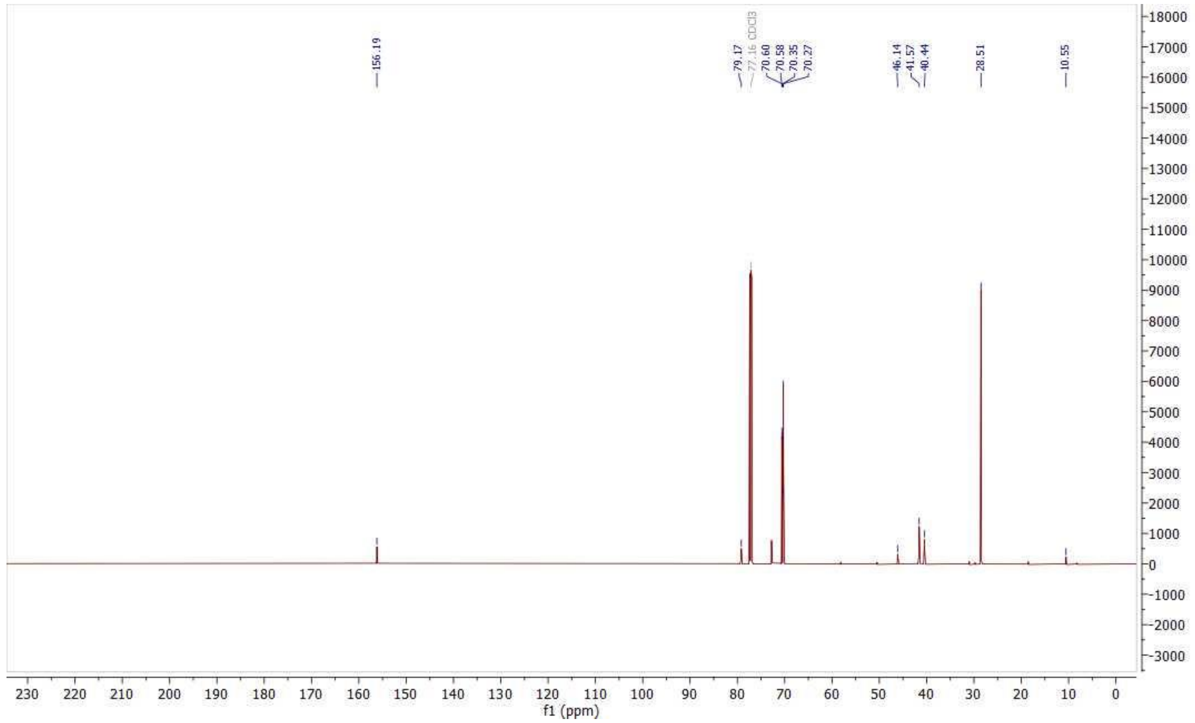

^{1}H-NMR spectrum of **1c**

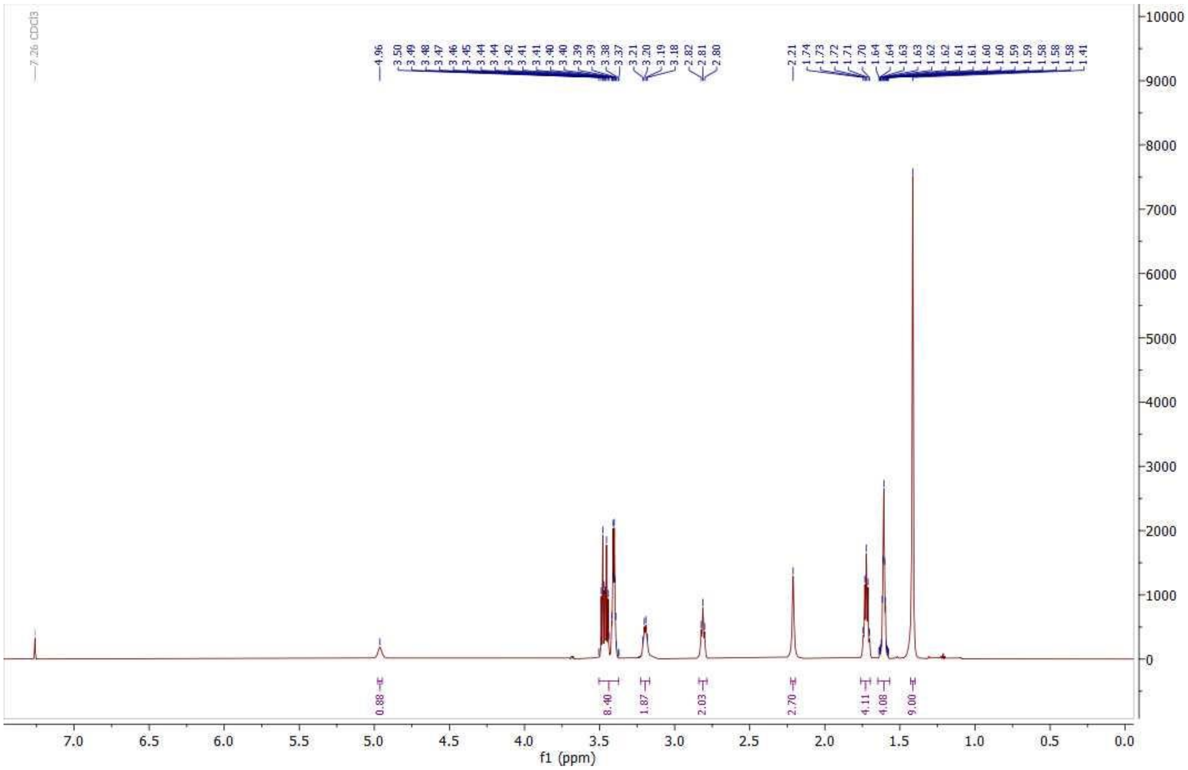

^{13}C-NMR spectrum of **1c**

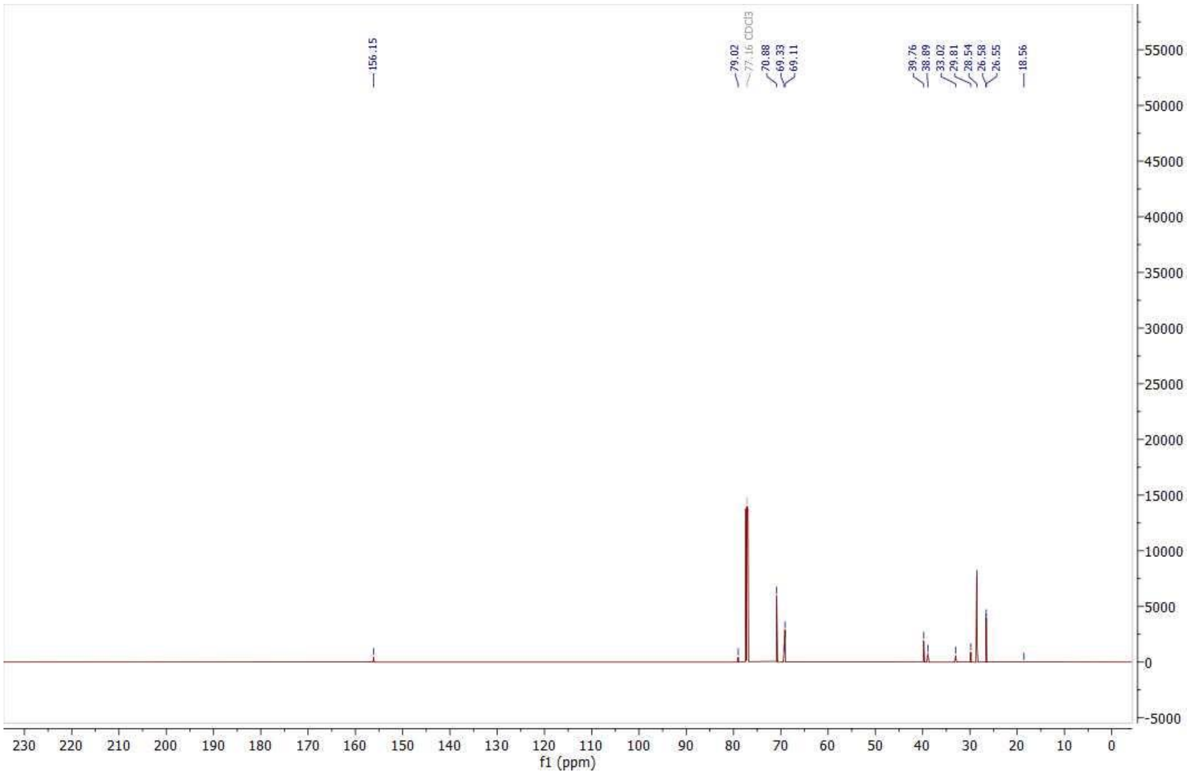

^{1}H-NMR spectrum of **1d**

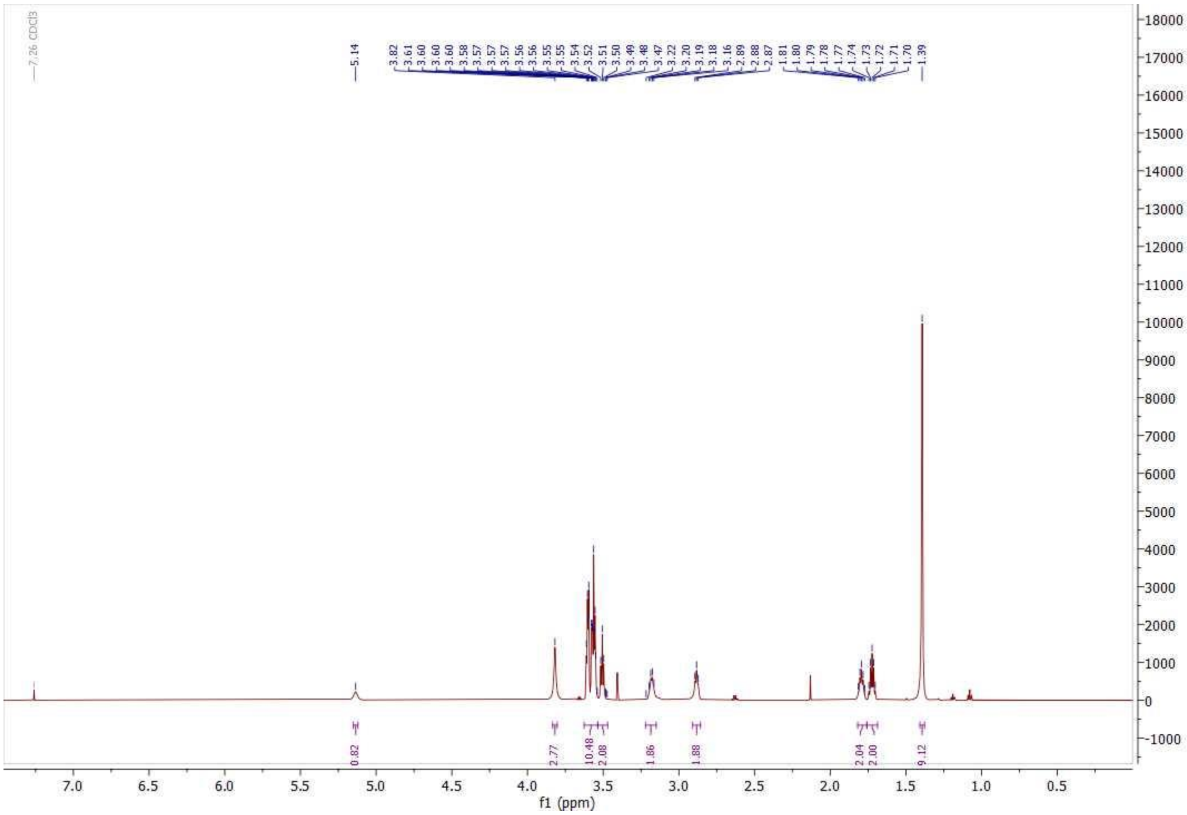

^{13}C-NMR spectrum of **1d**

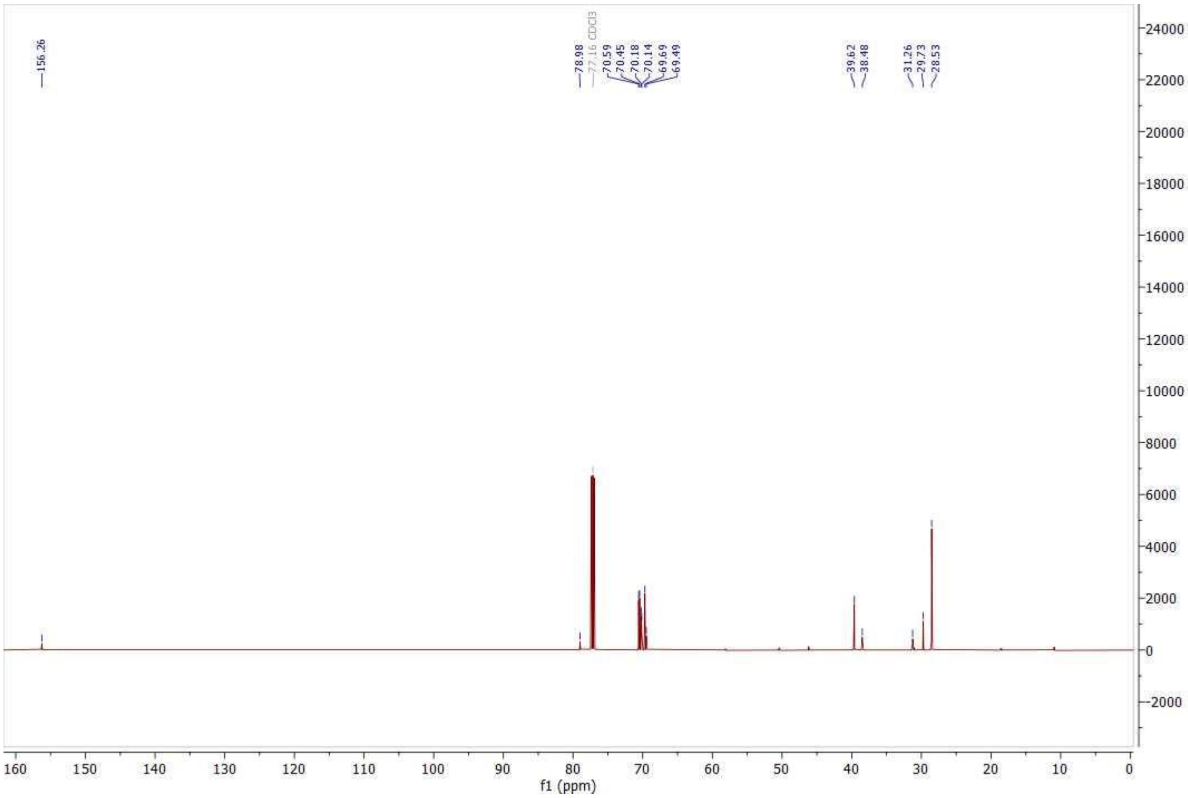

^{1}H-NMR spectrum of **1e**

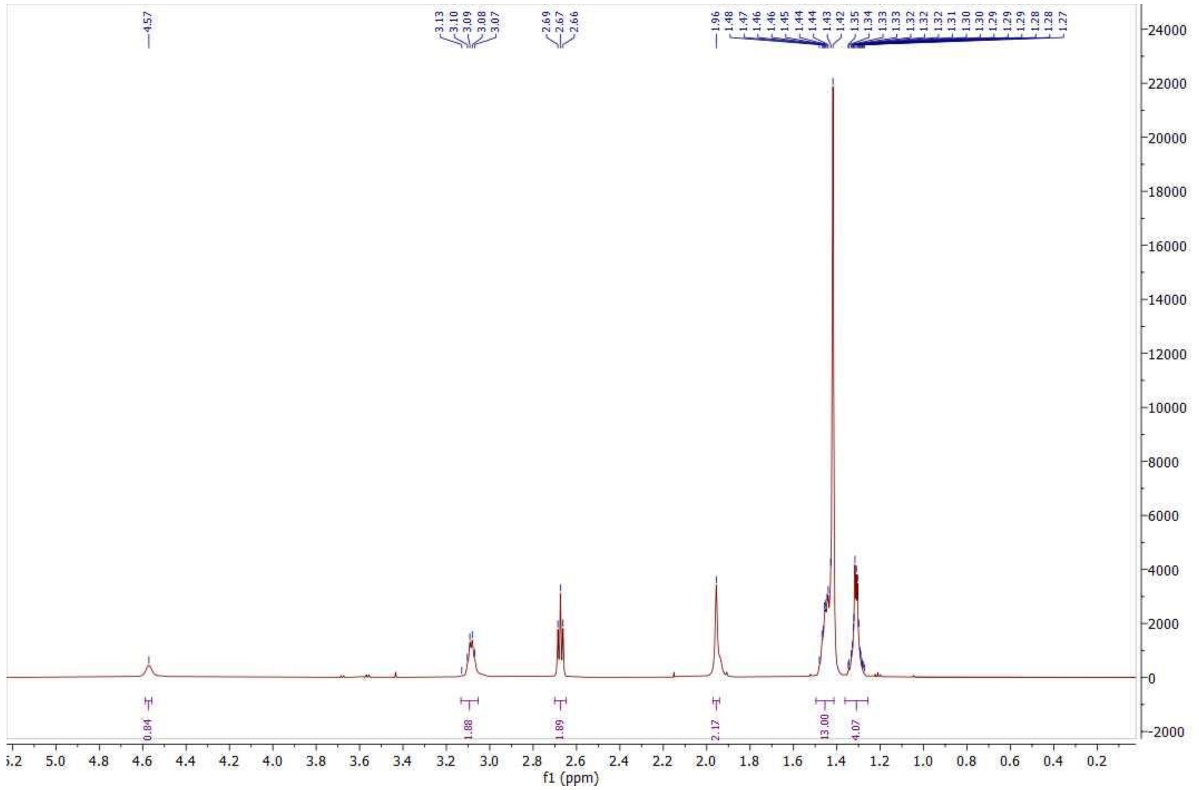

^{13}C-NMR spectrum of **1e**

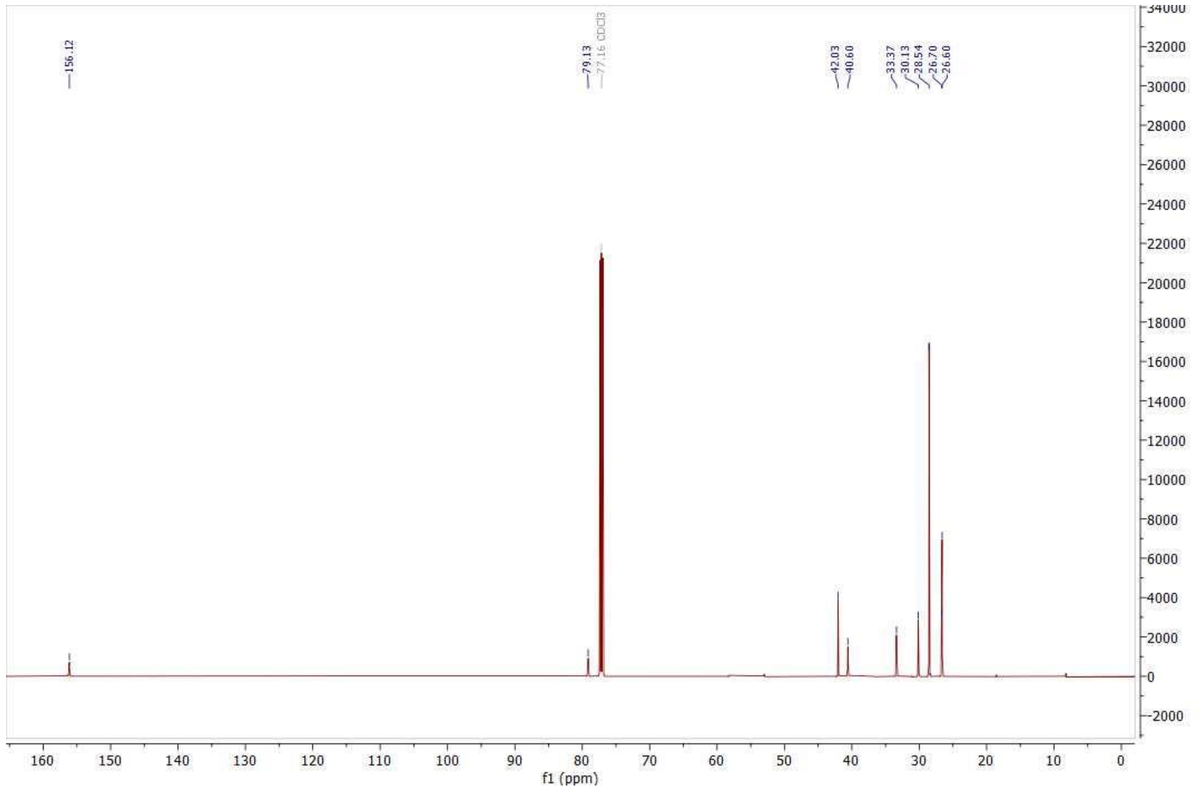

^{1}H-NMR spectrum of **1f**

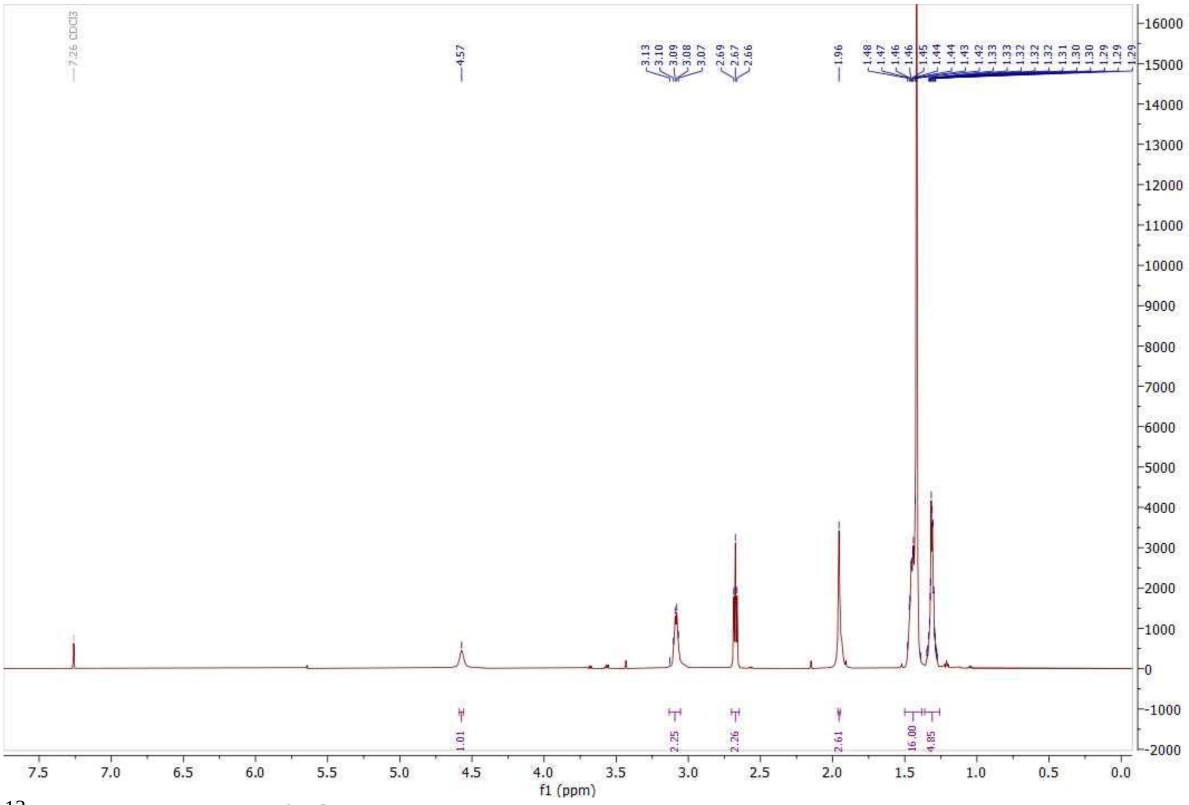

^{13}C-NMR spectrum of **1f**

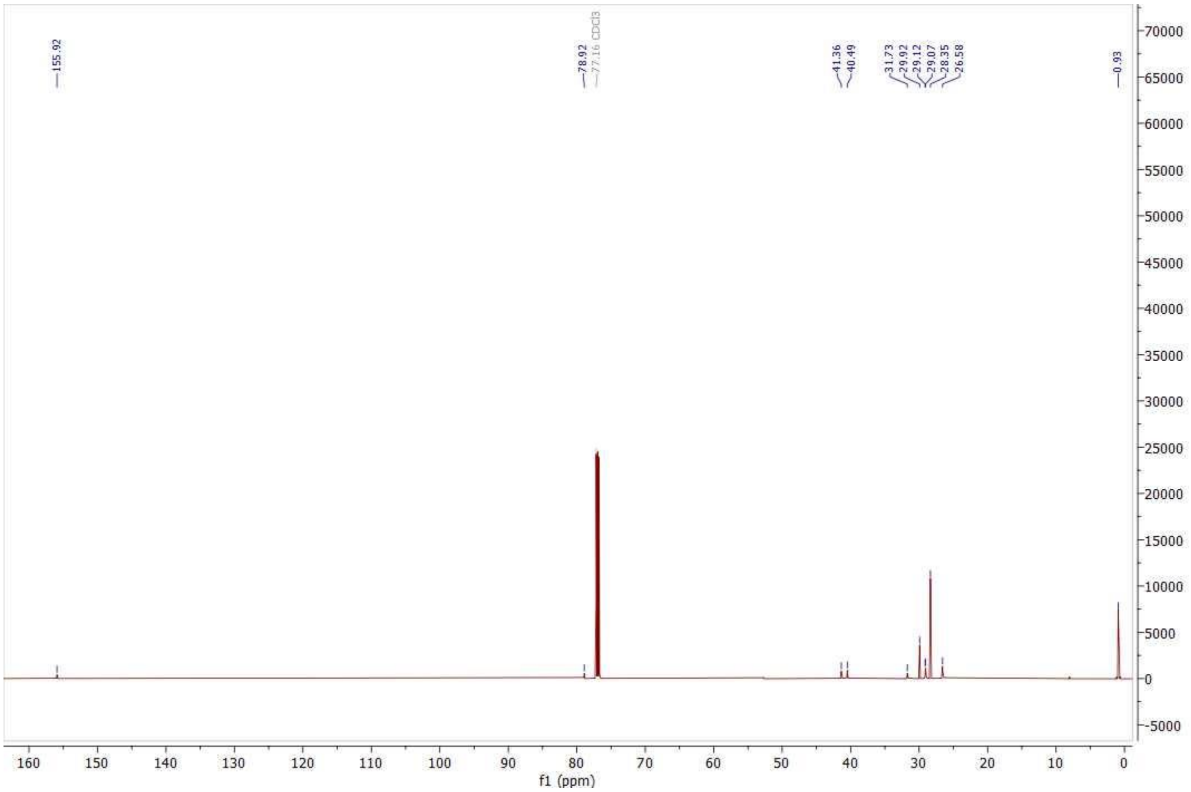

^{1}H-NMR spectrum of **2a**

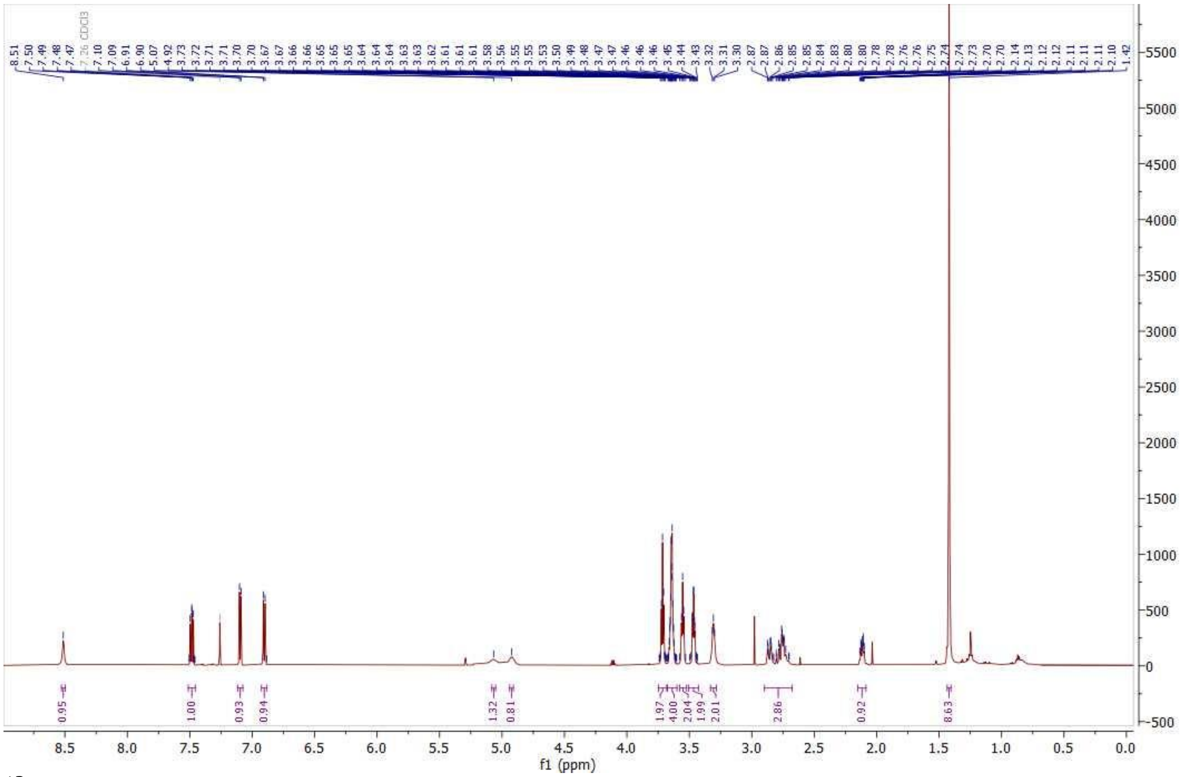

^{13}C-NMR spectrum of **2a**

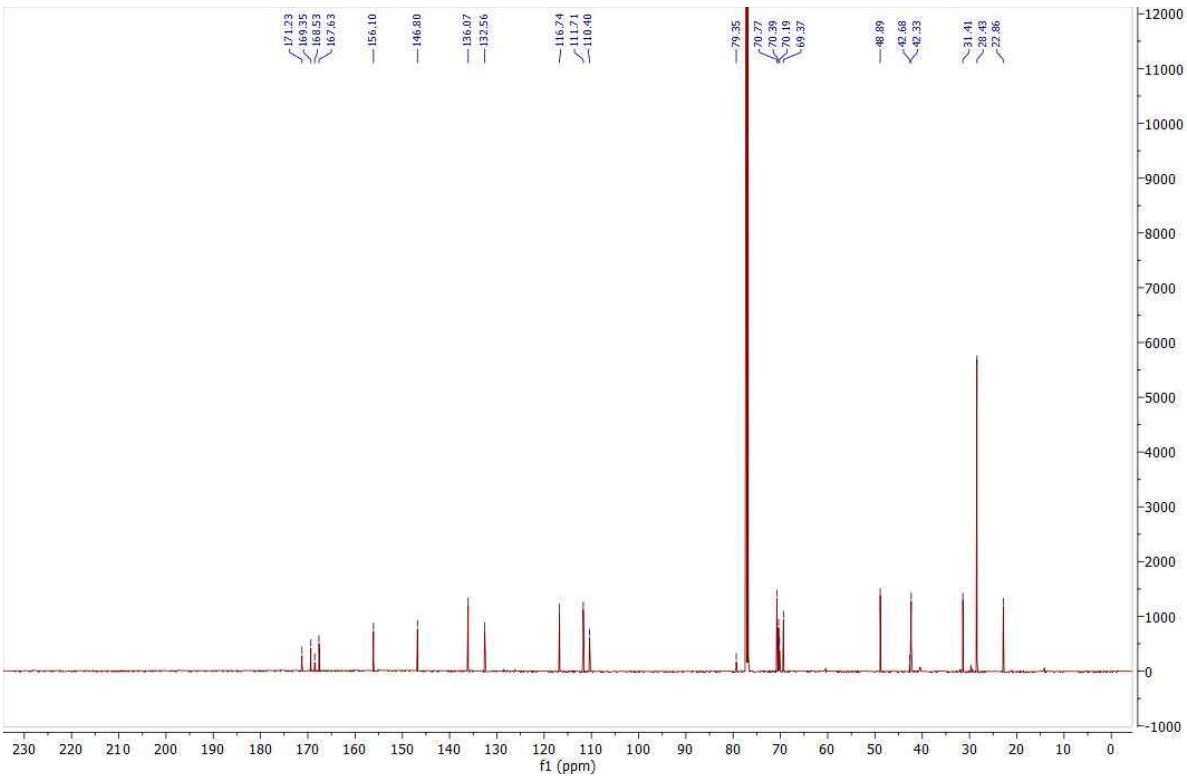

^{1}H-NMR spectrum of **2b**

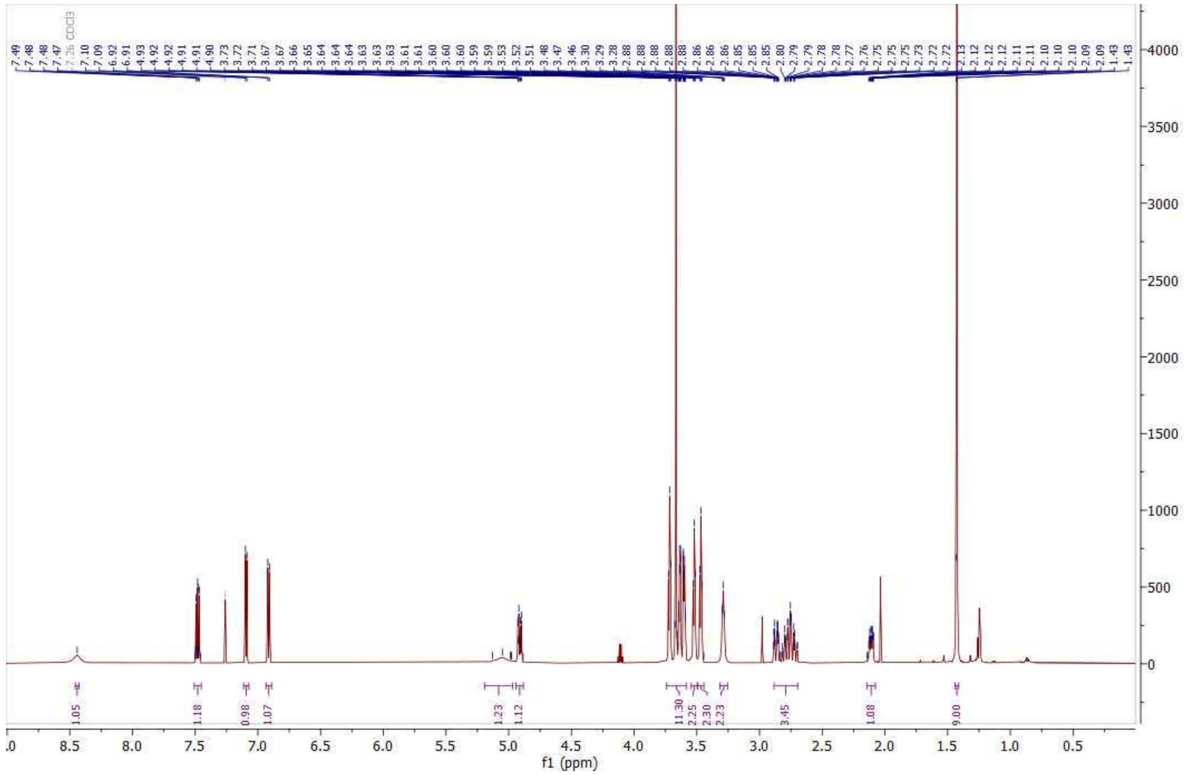

^{13}C-NMR spectrum of **2b**

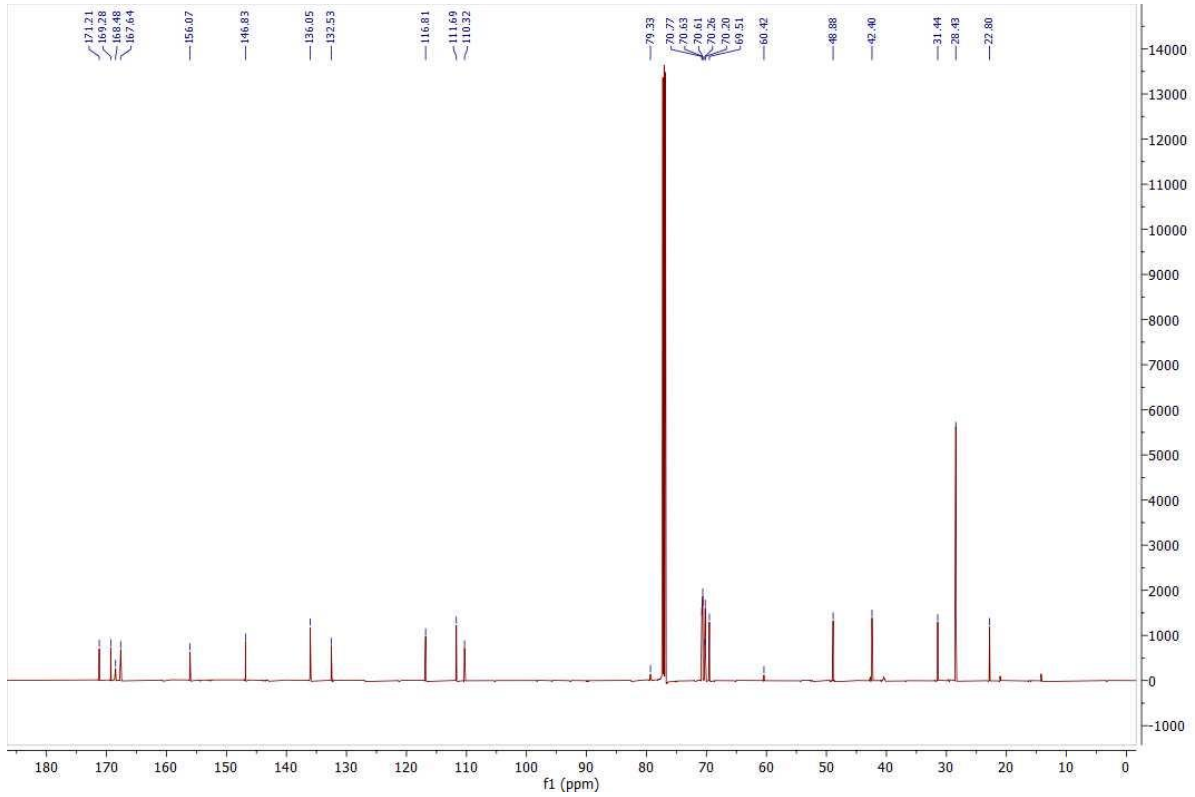

^{1}H-NMR spectrum of **2c**

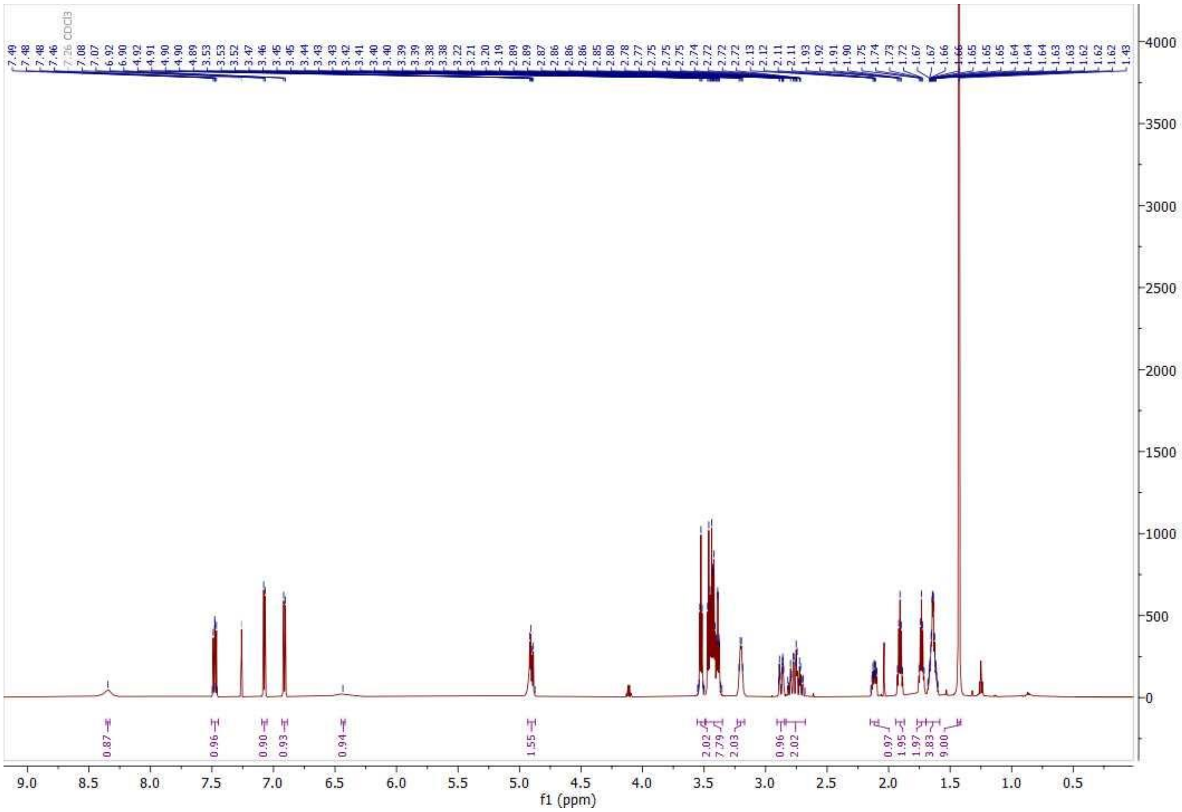

^{13}C-NMR spectrum of **2c**

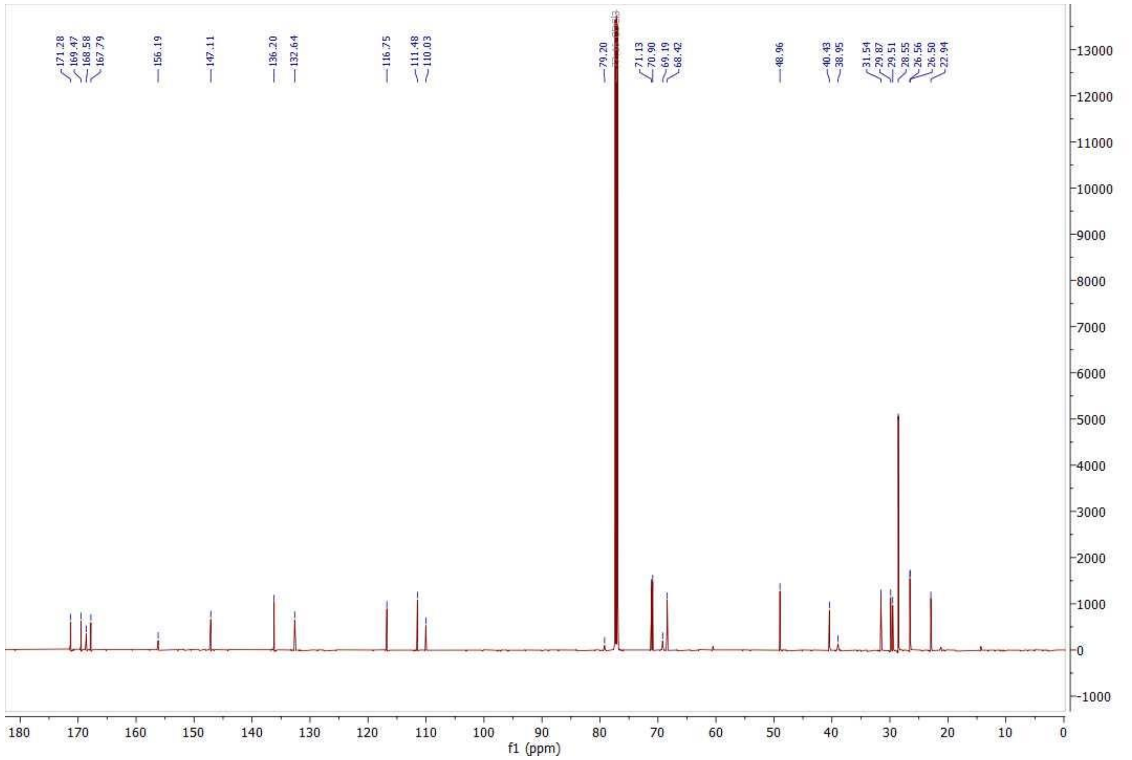

^{1}H-NMR spectrum **2d**

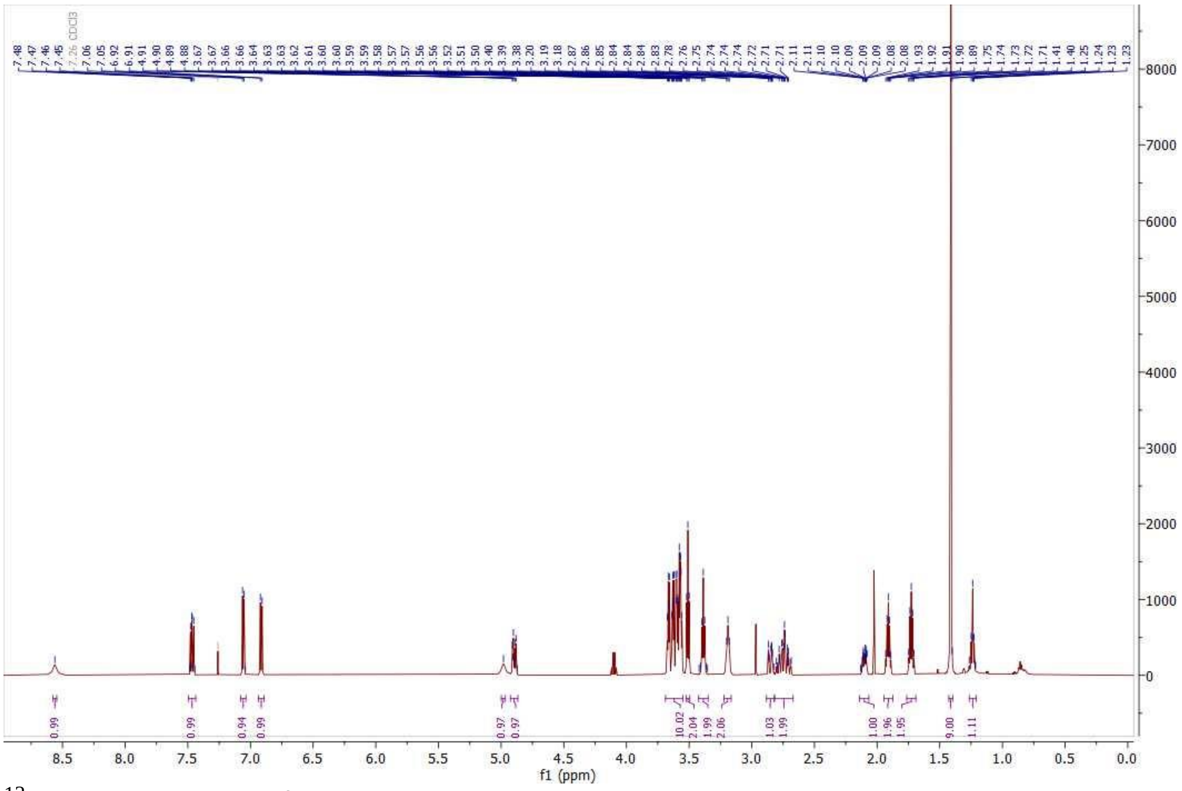

^{13}C-NMR spectrum of **2d**

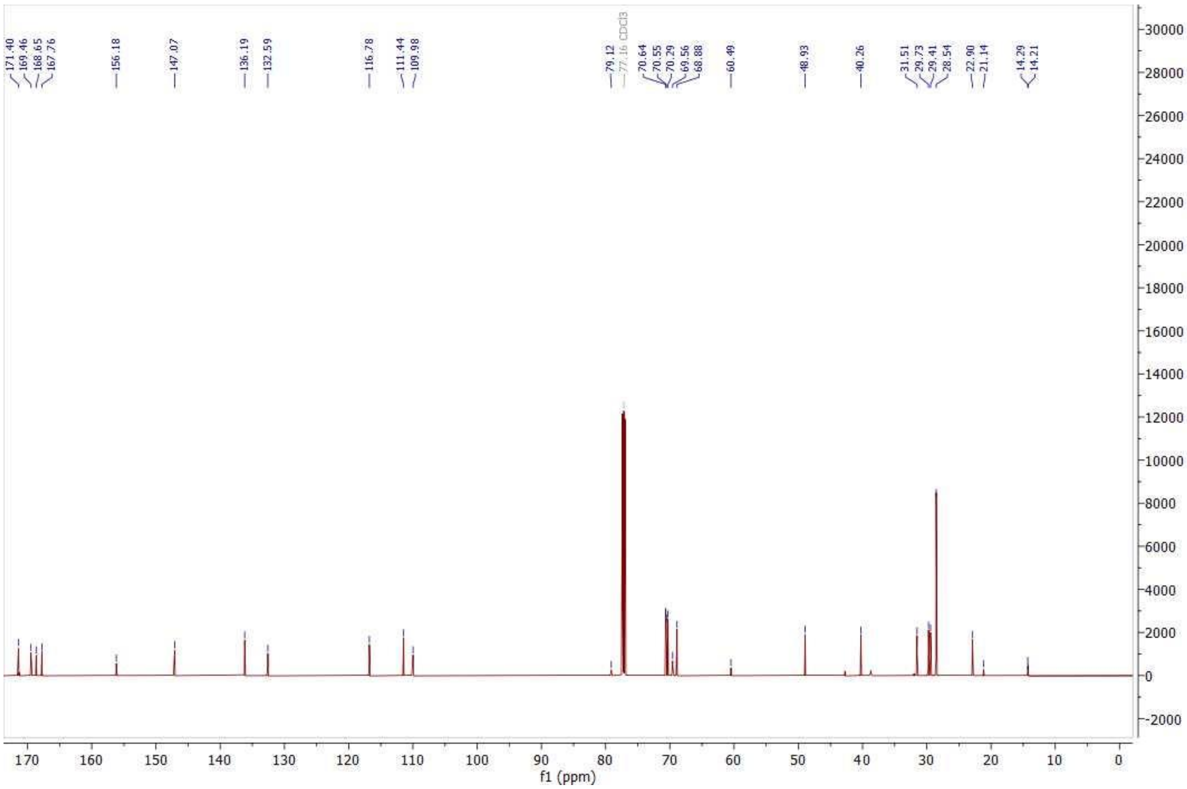

^{1}H-NMR spectrum of **2e**

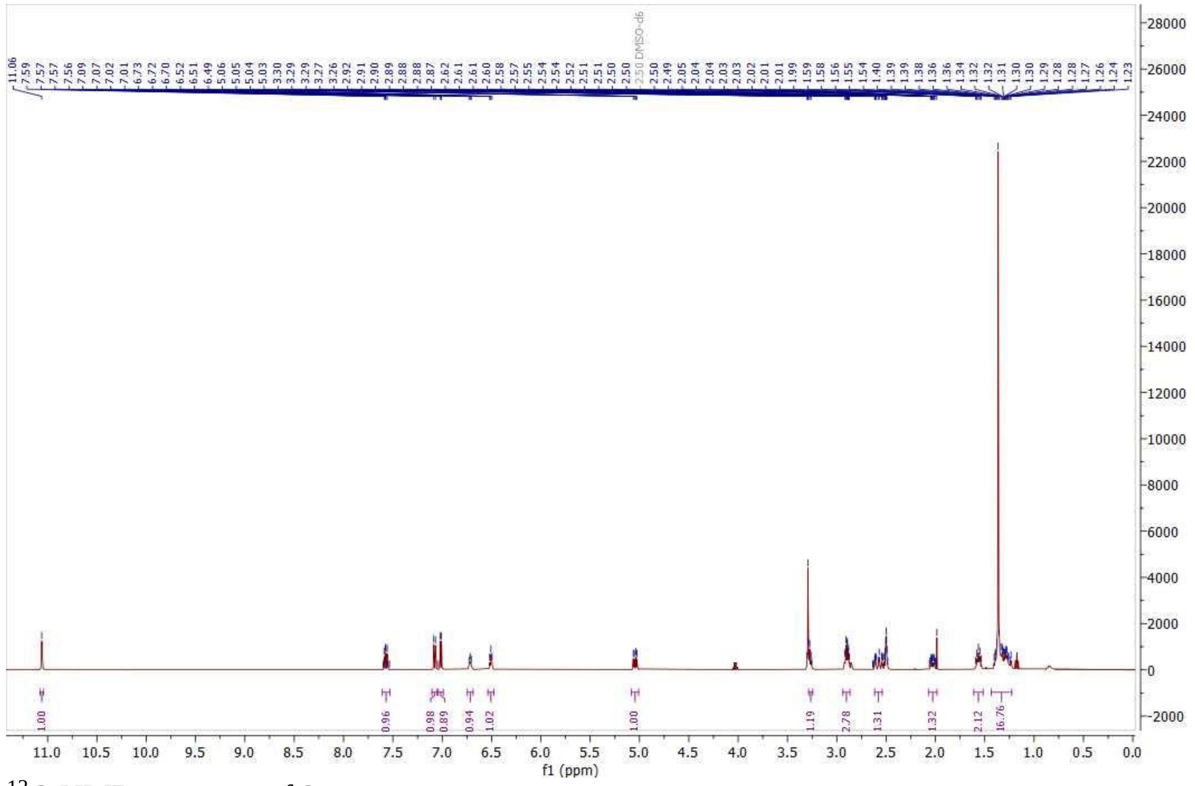

^{13}C-NMR spectrum of **2e**

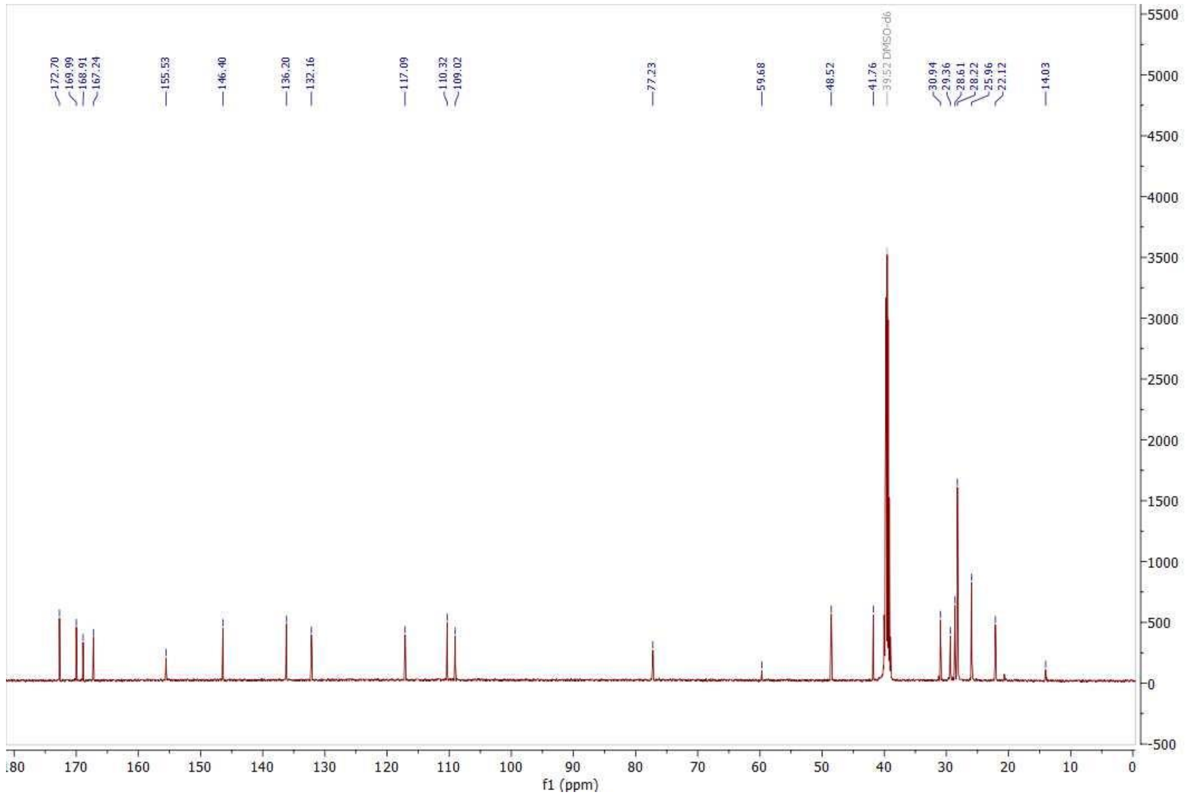

^{1}H-NMR spectrum of **2f**

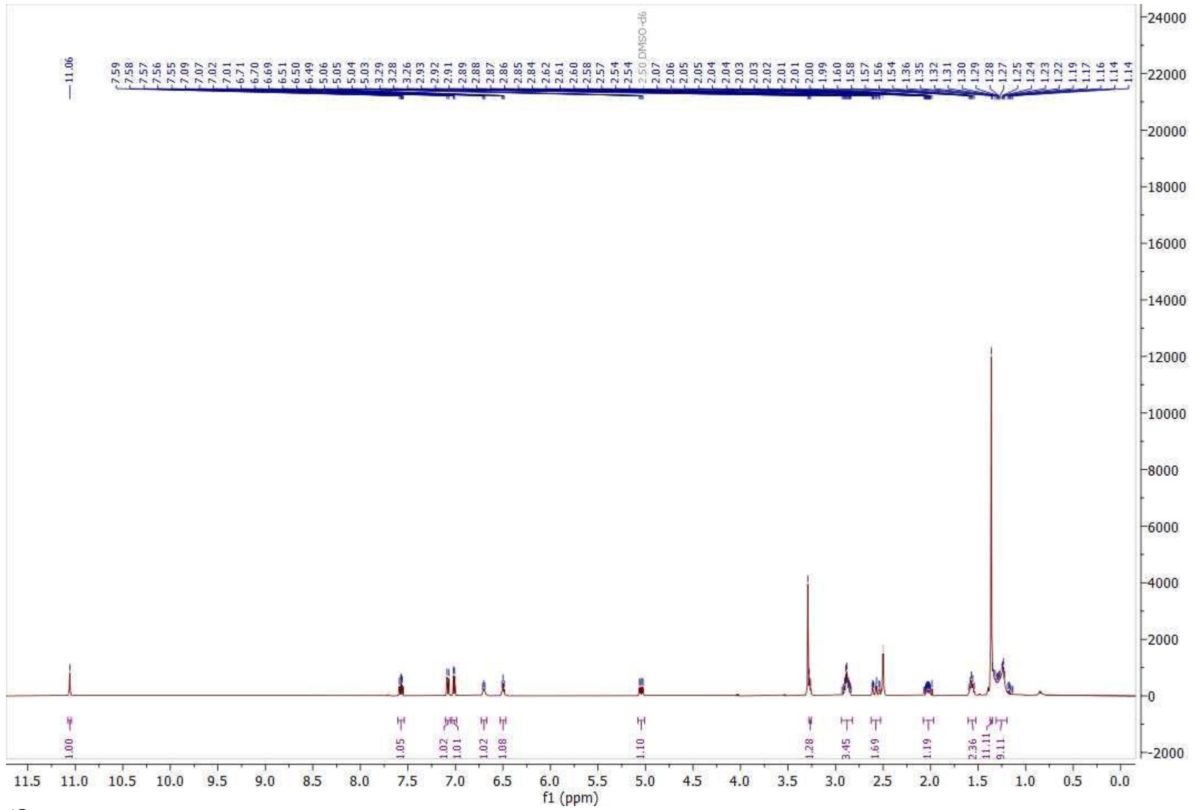

^{13}C-NMR spectrum of **2f**

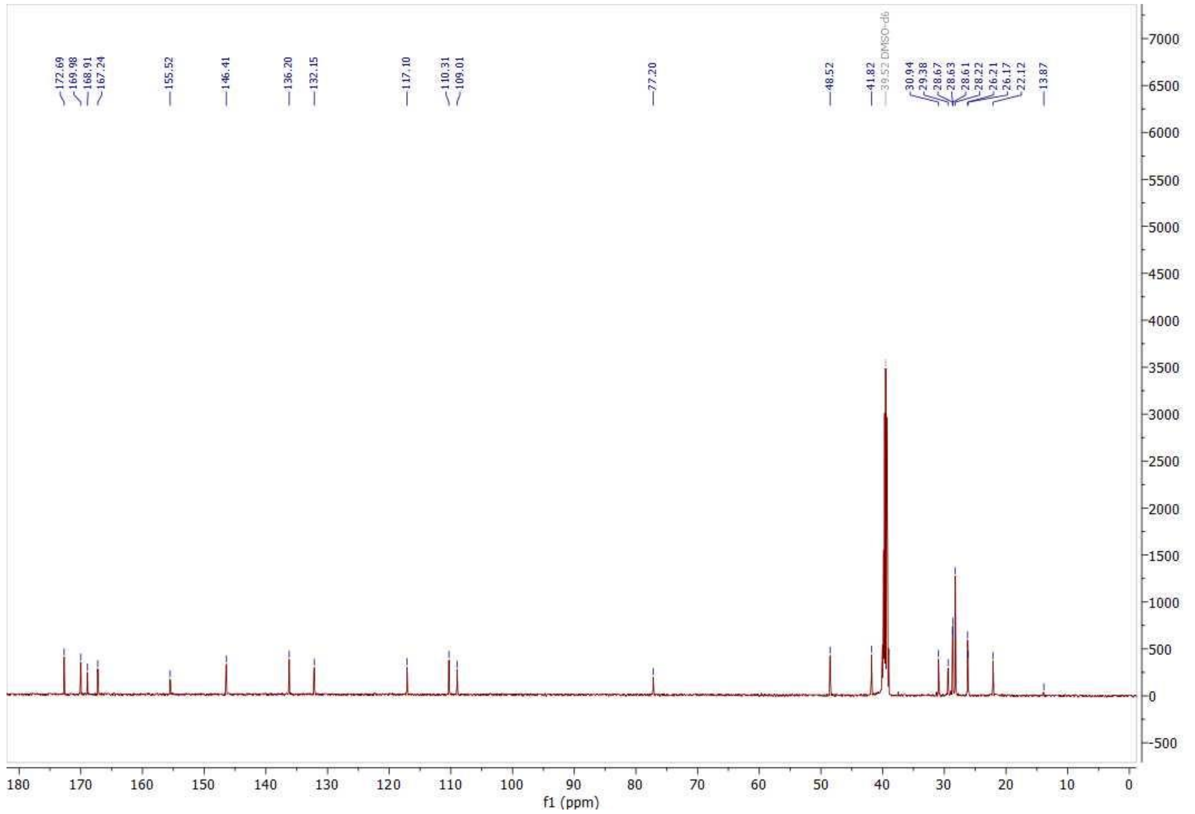

^{1}H-NMR spectrum of **2g**

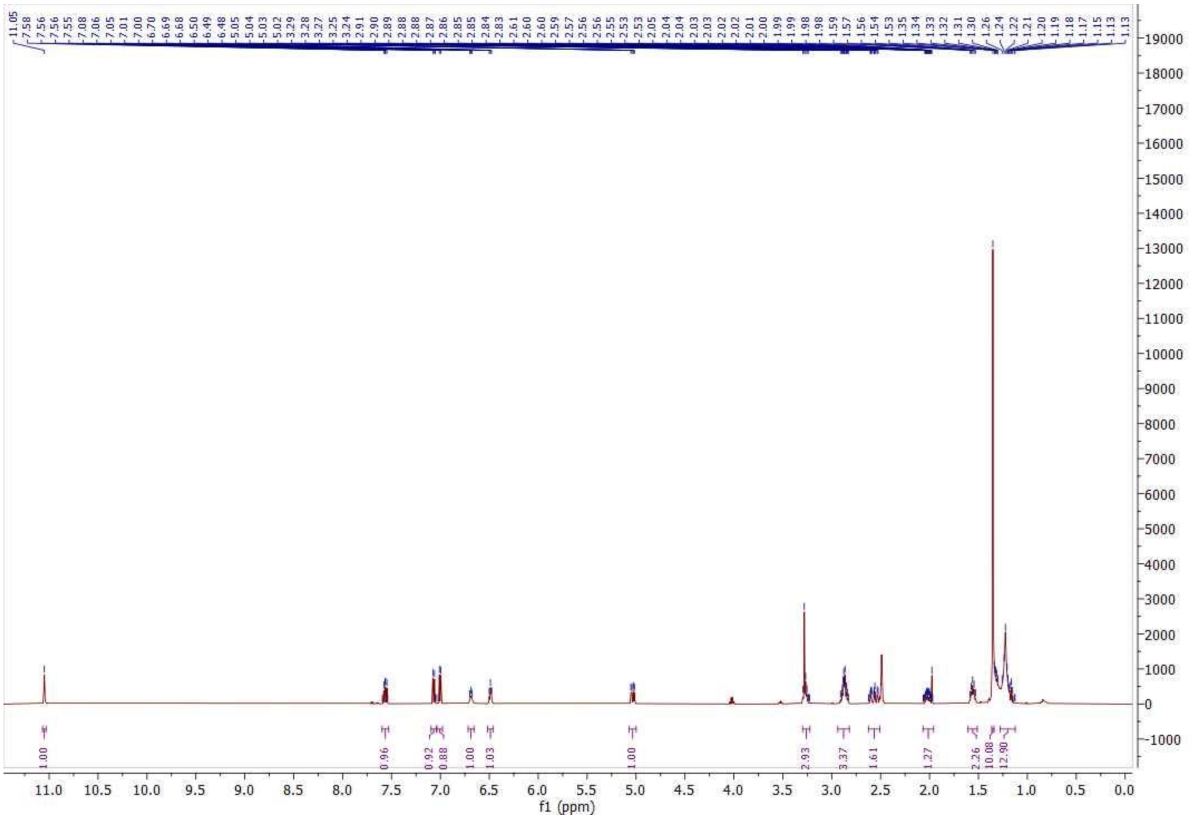

^{13}C-NMR spectrum of **2g**

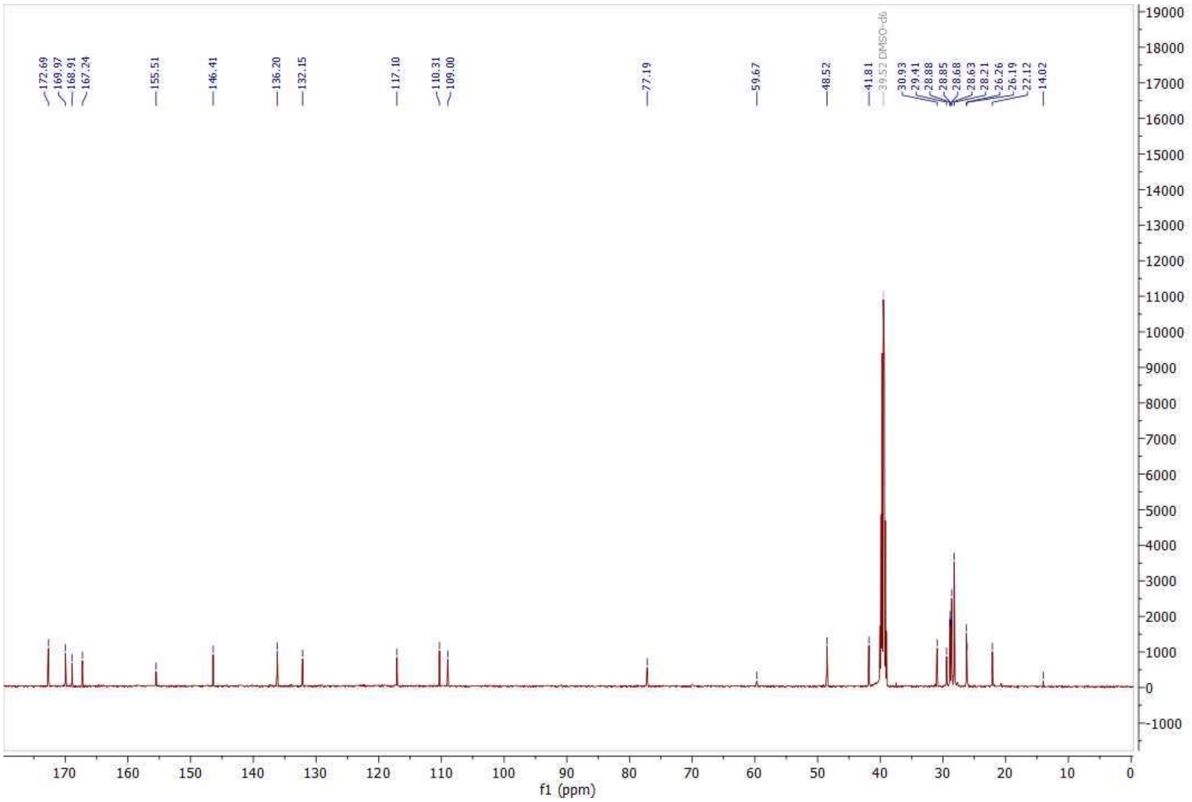

^{1}H-NMR spectrum of **3a**

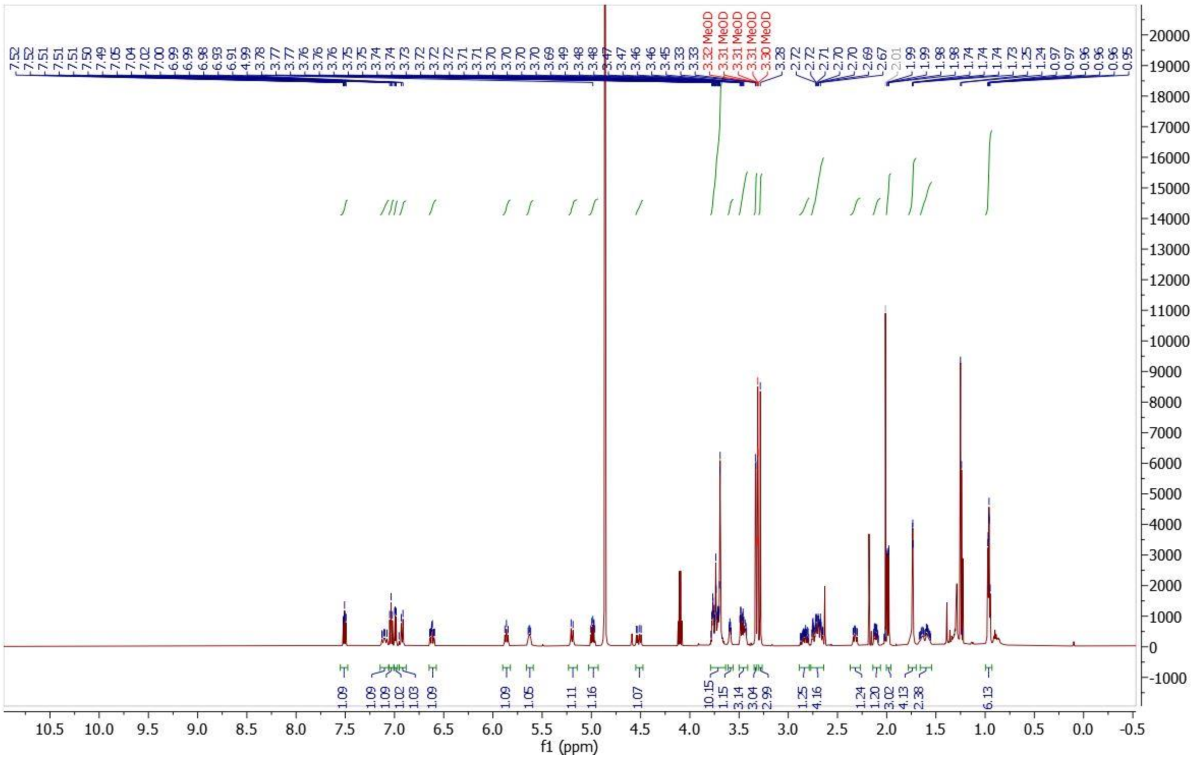

^{13}C-NMR spectrum of **3a**

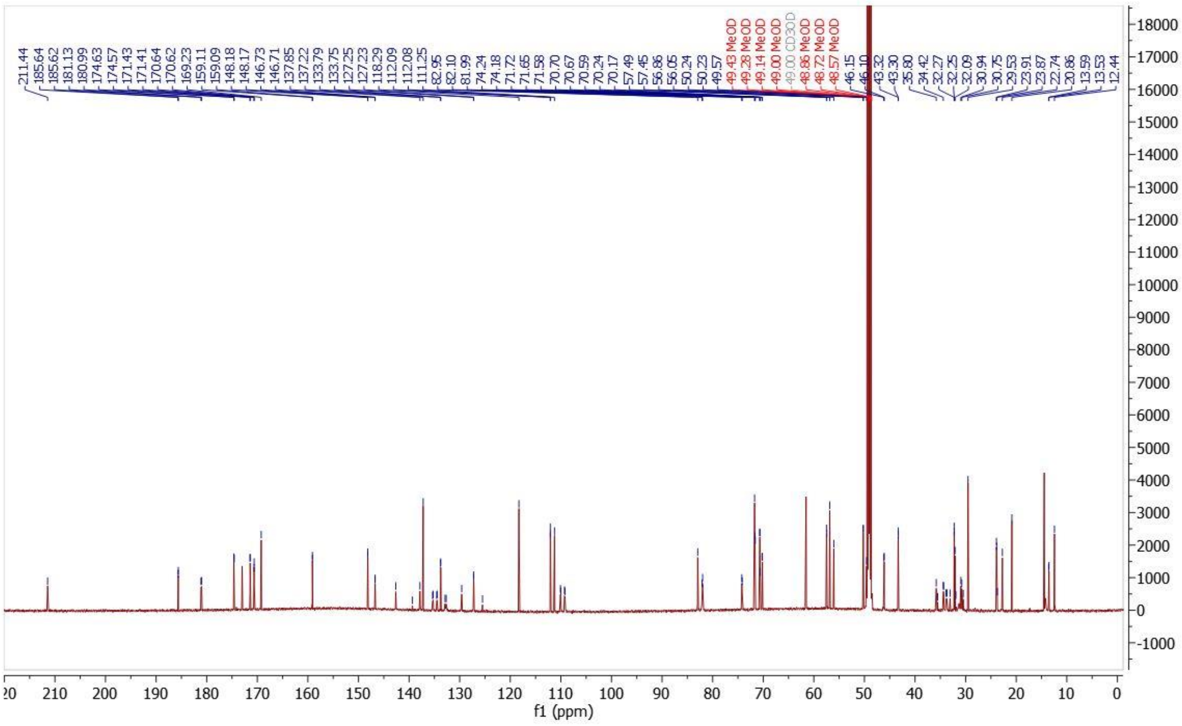

^{1}H-NMR spectrum of **3b**

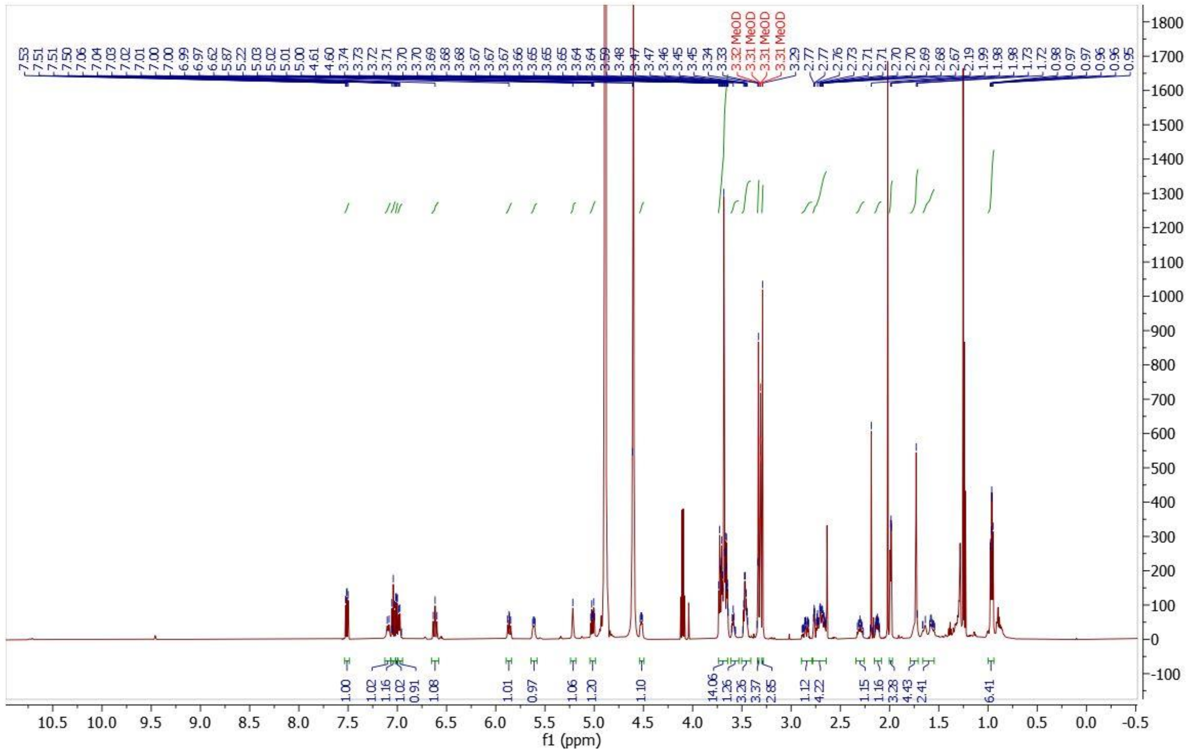

^{13}C-NMR spectrum of **3b**

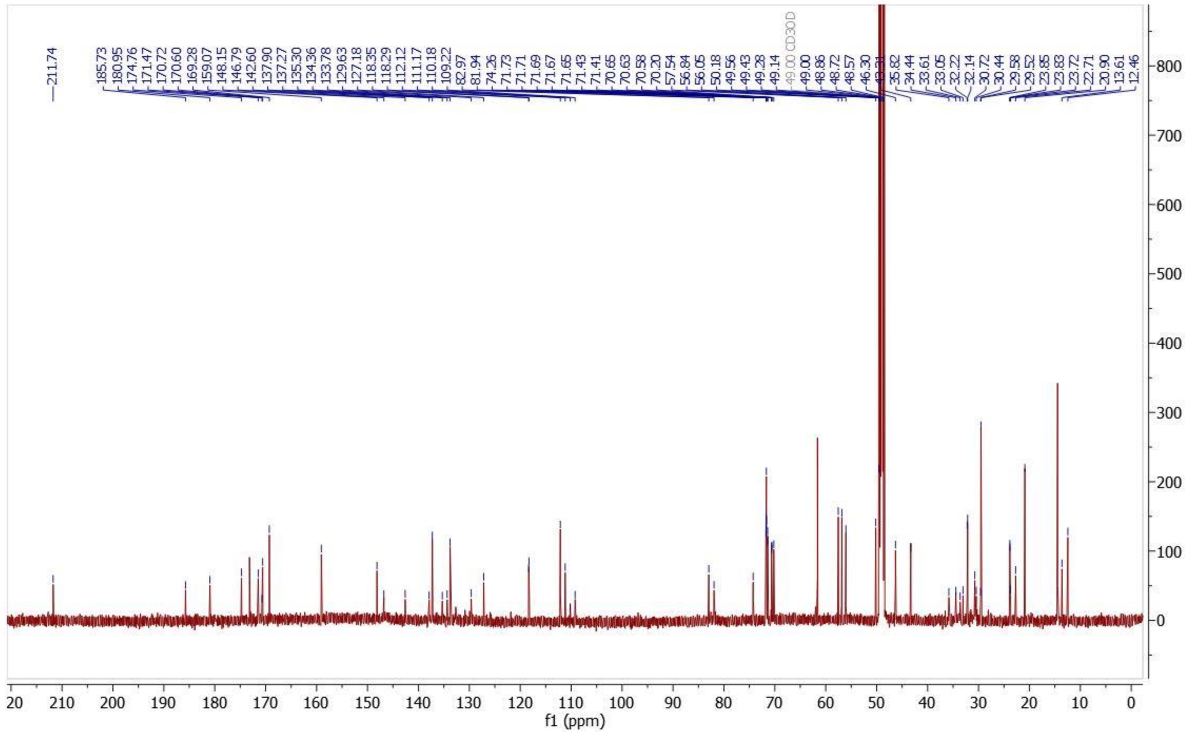

^{1}H-NMR spectrum of **3c**

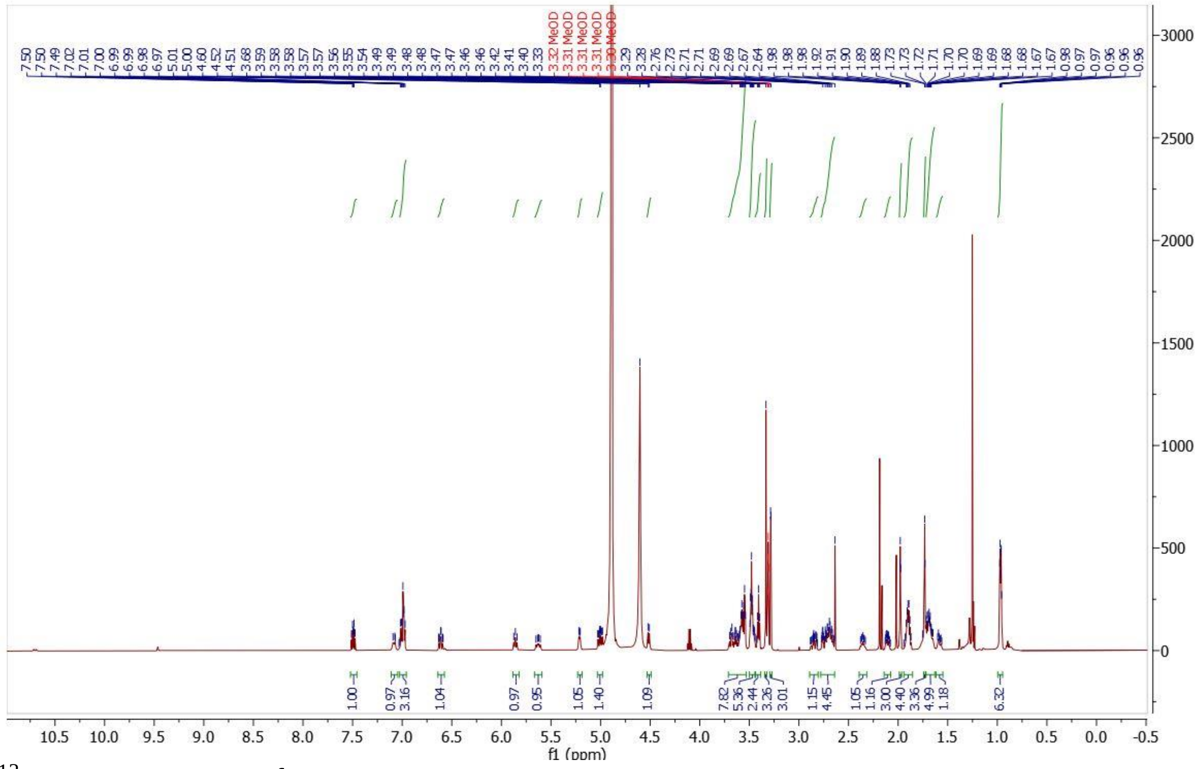

^{13}C-NMR spectrum of **3c**

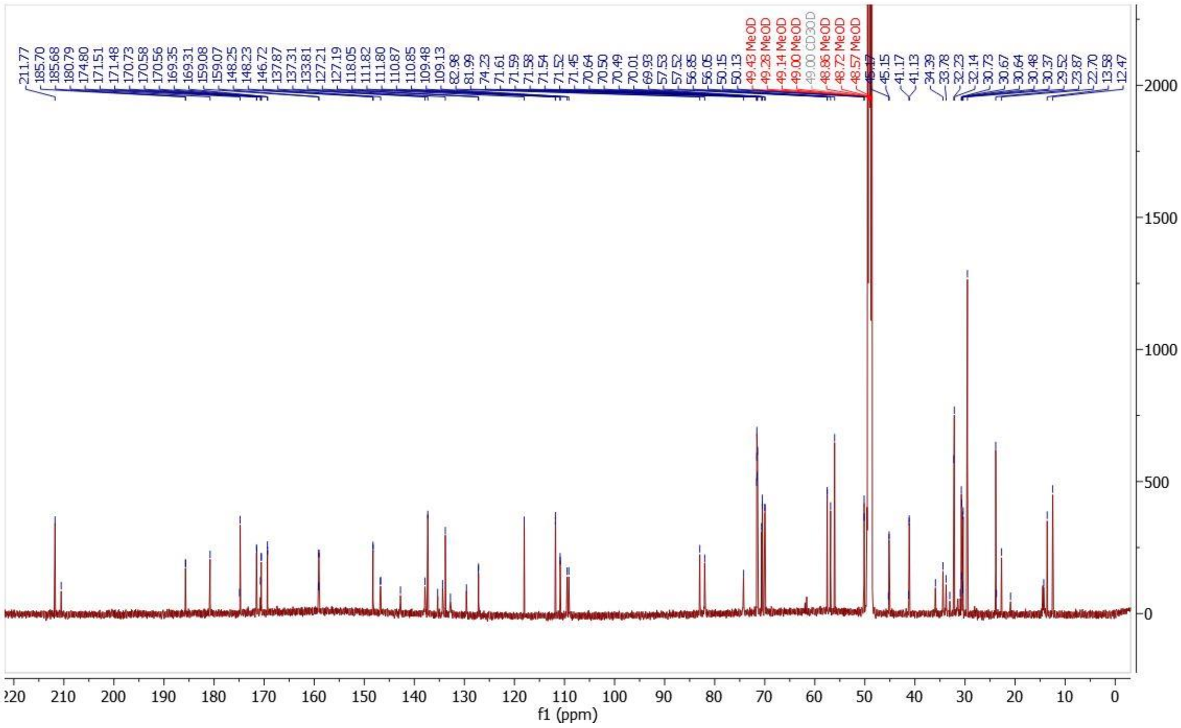

^{1}H-NMR spectrum of **3d**

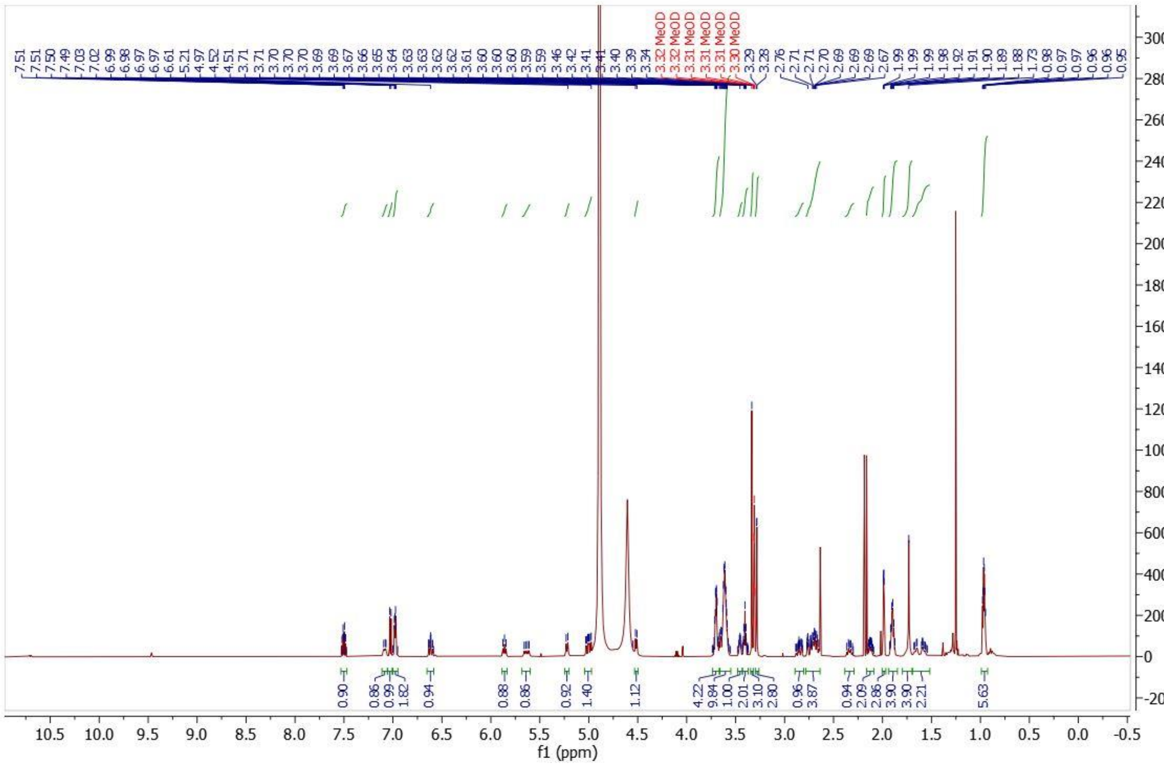

^{13}C-NMR spectrum of **3d**

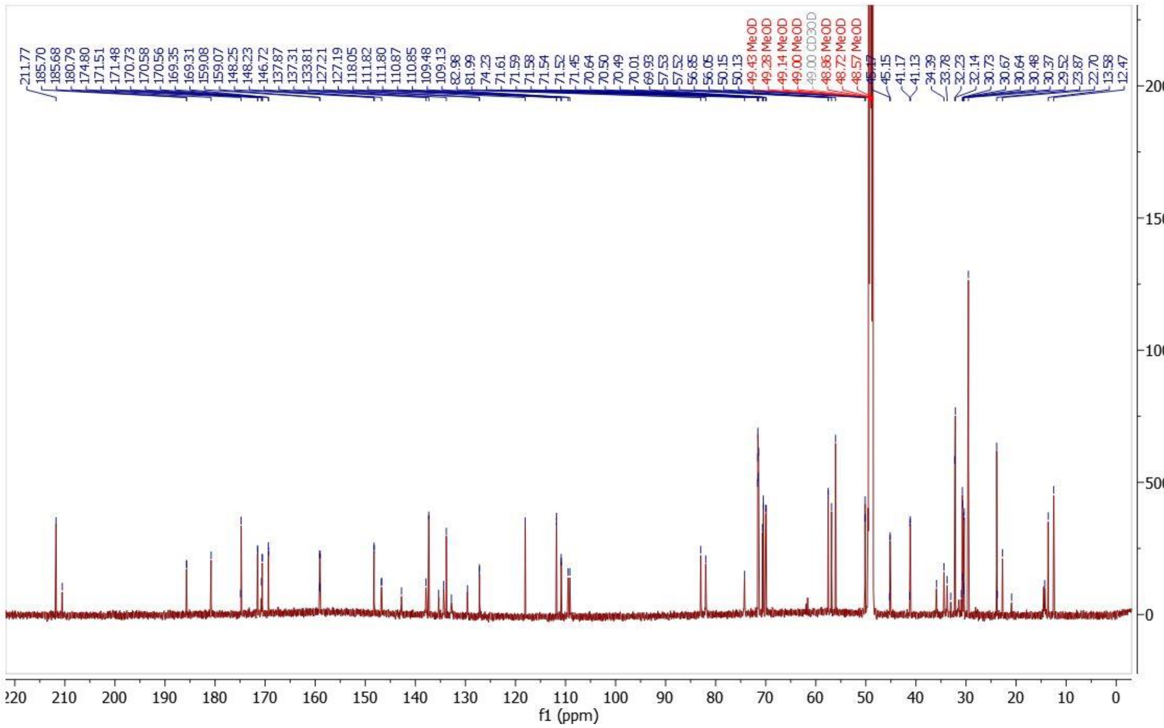

^{1}H-NMR spectrum of **3e**

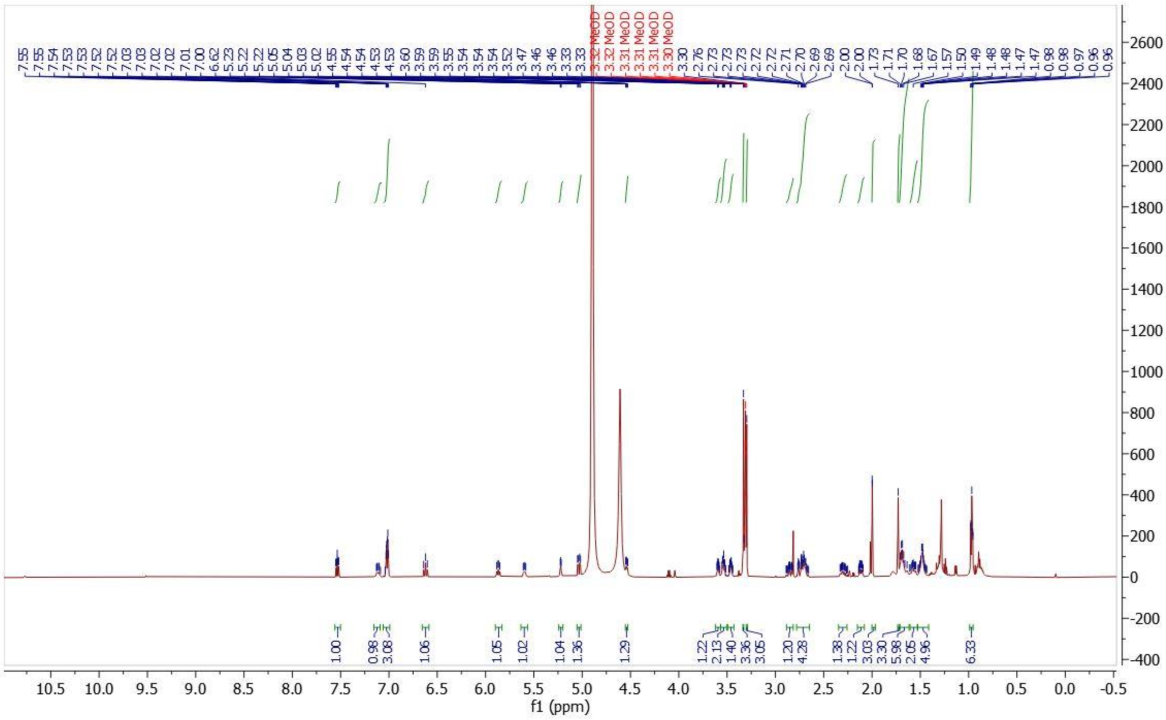

^{13}C-NMR spectrum of **3e**

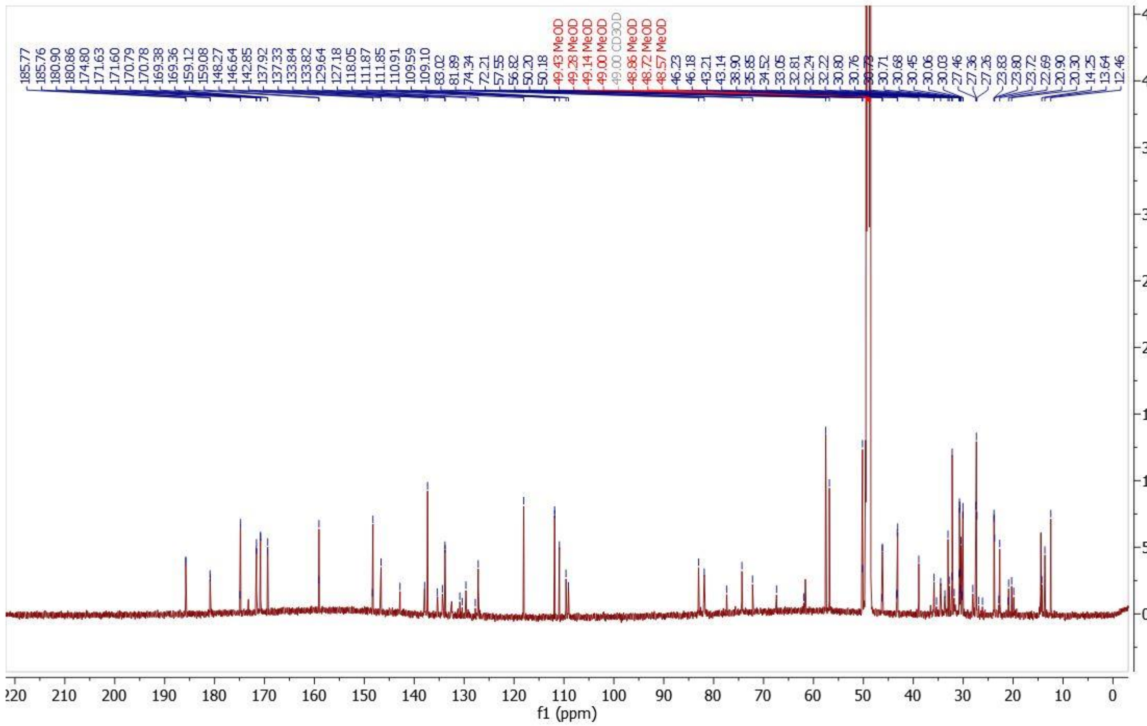

^{1}H-NMR spectrum of **3f**

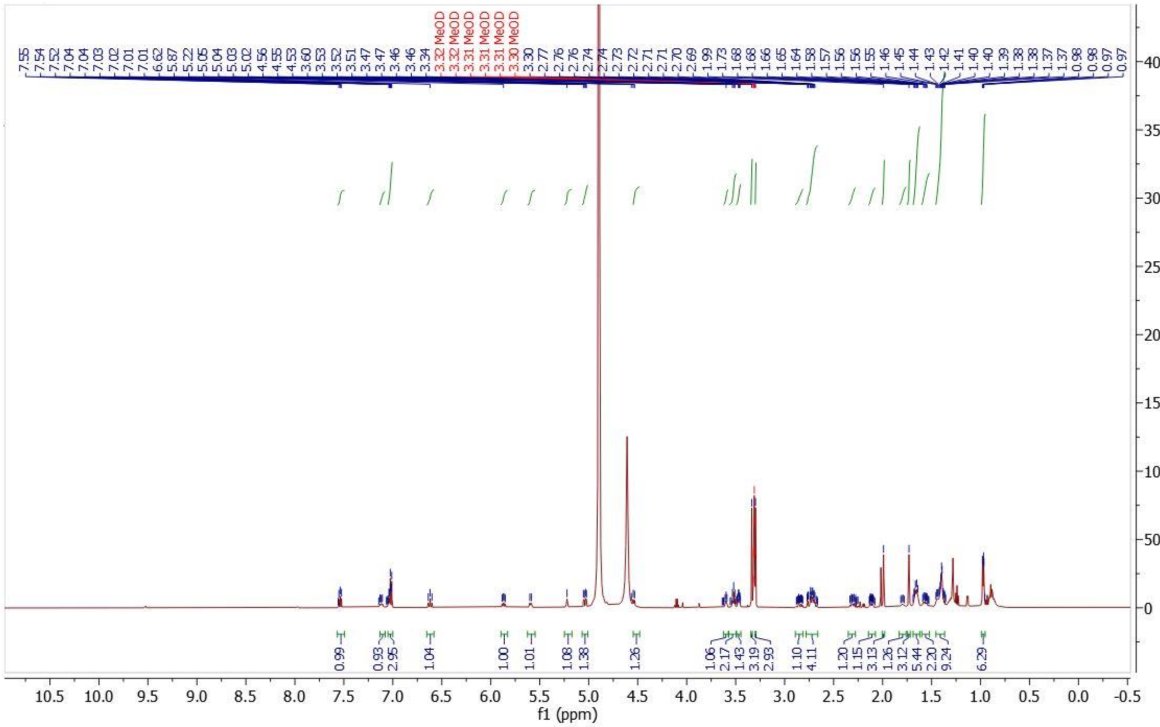

^{13}C-NMR spectrum of **3f**

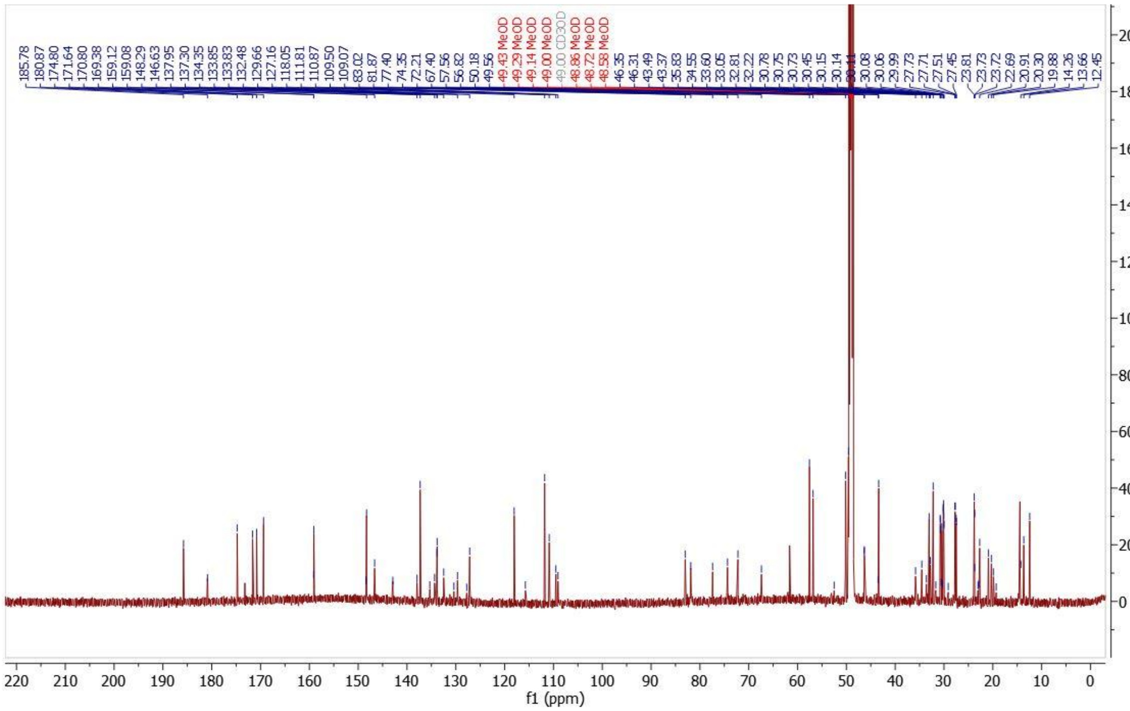

^{1}H-NMR spectrum of **3g**

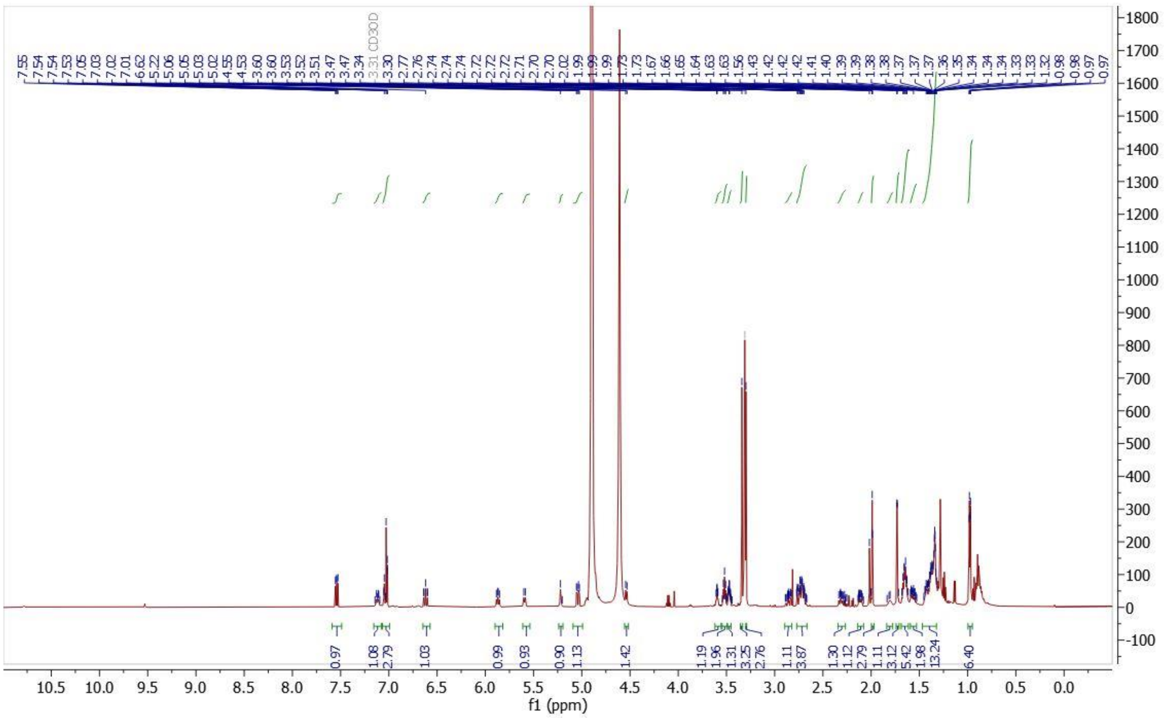

^{13}C-NMR spectrum of **3g**

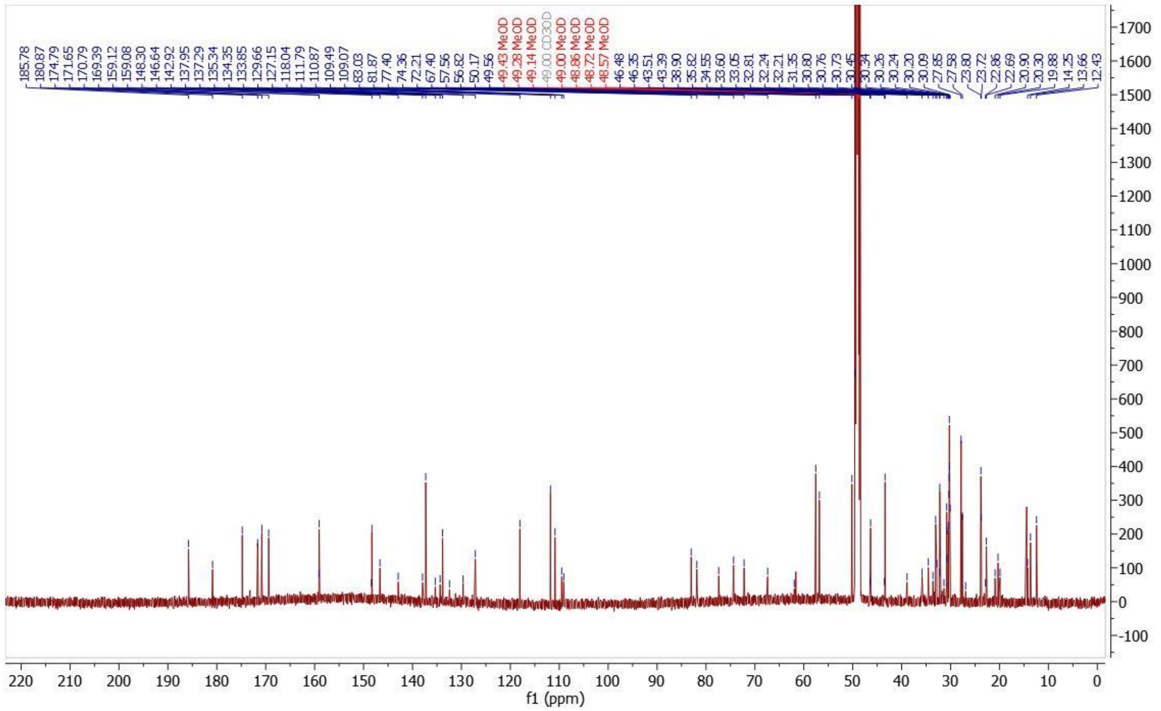

^{1}H-NMR spectrum of **4**

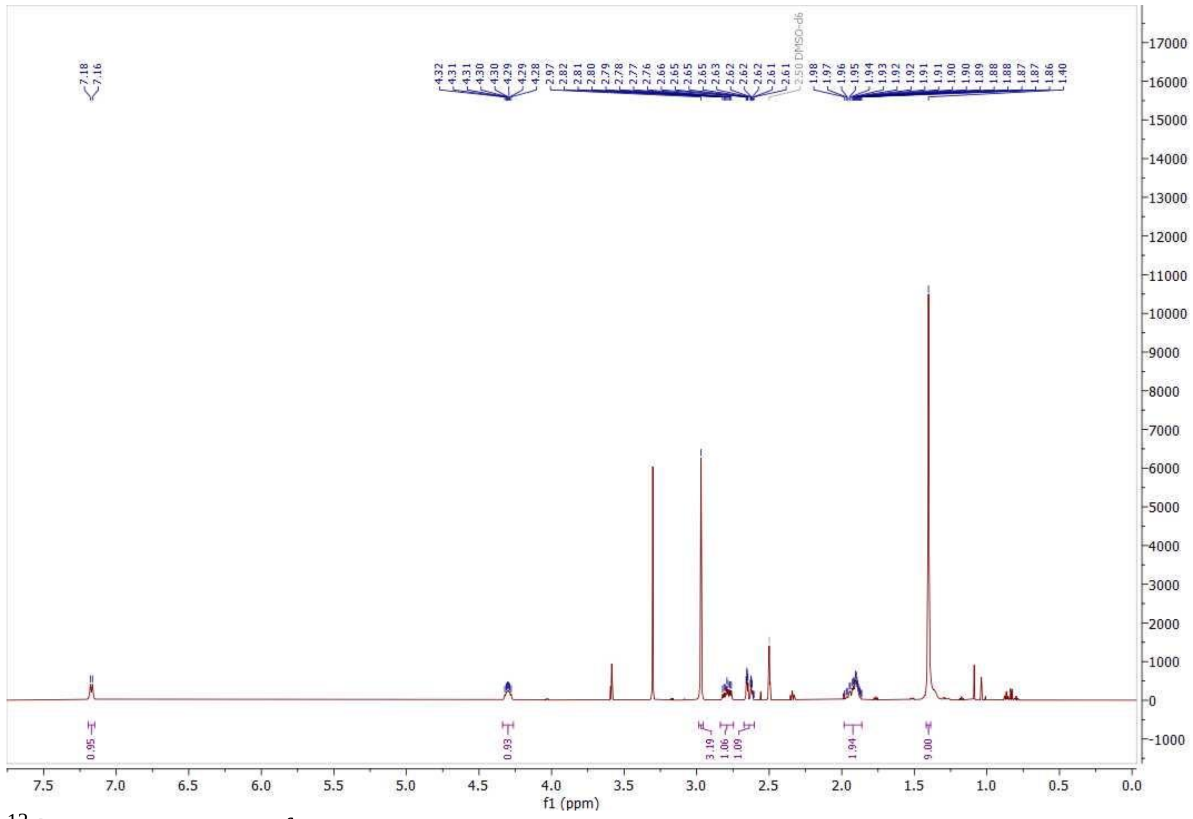

^{13}C-NMR spectrum of **4**

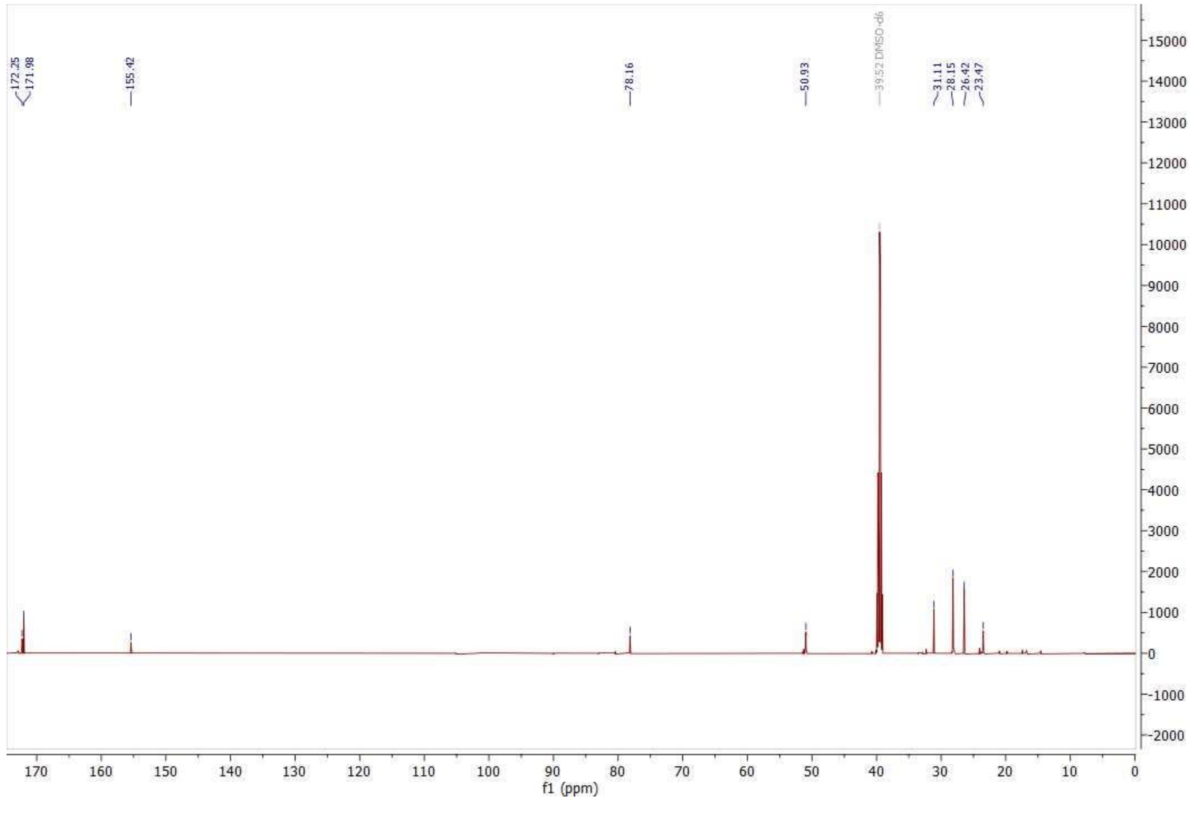

^{1}H-NMR spectrum of **5**

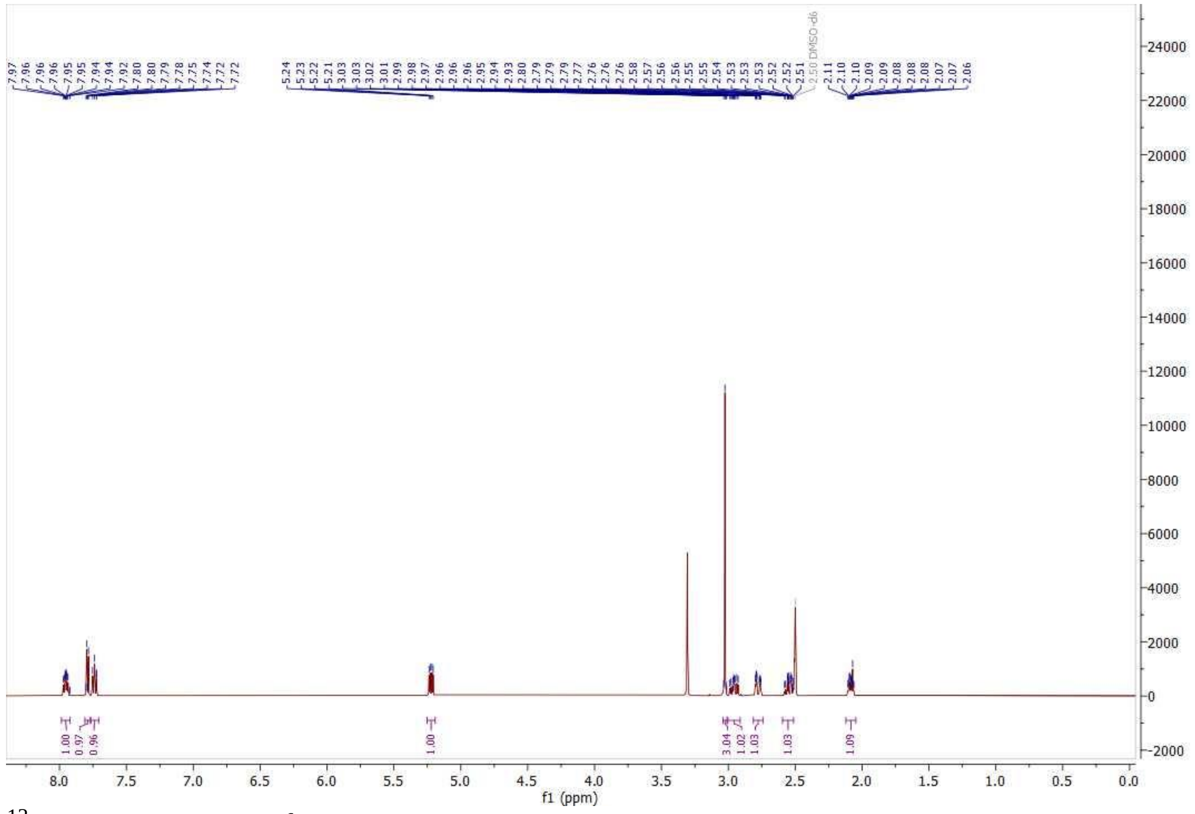

^{13}C-NMR spectrum of **5**

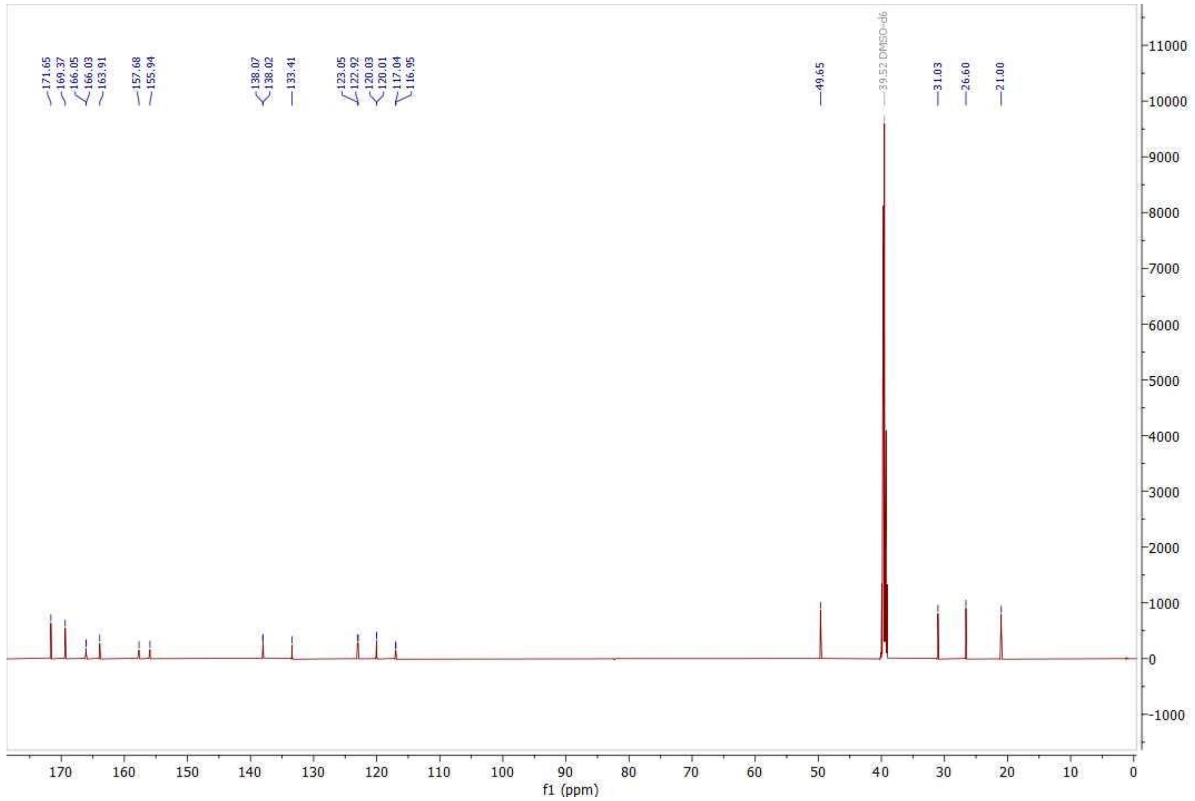

^{19}F-NMR of **5**

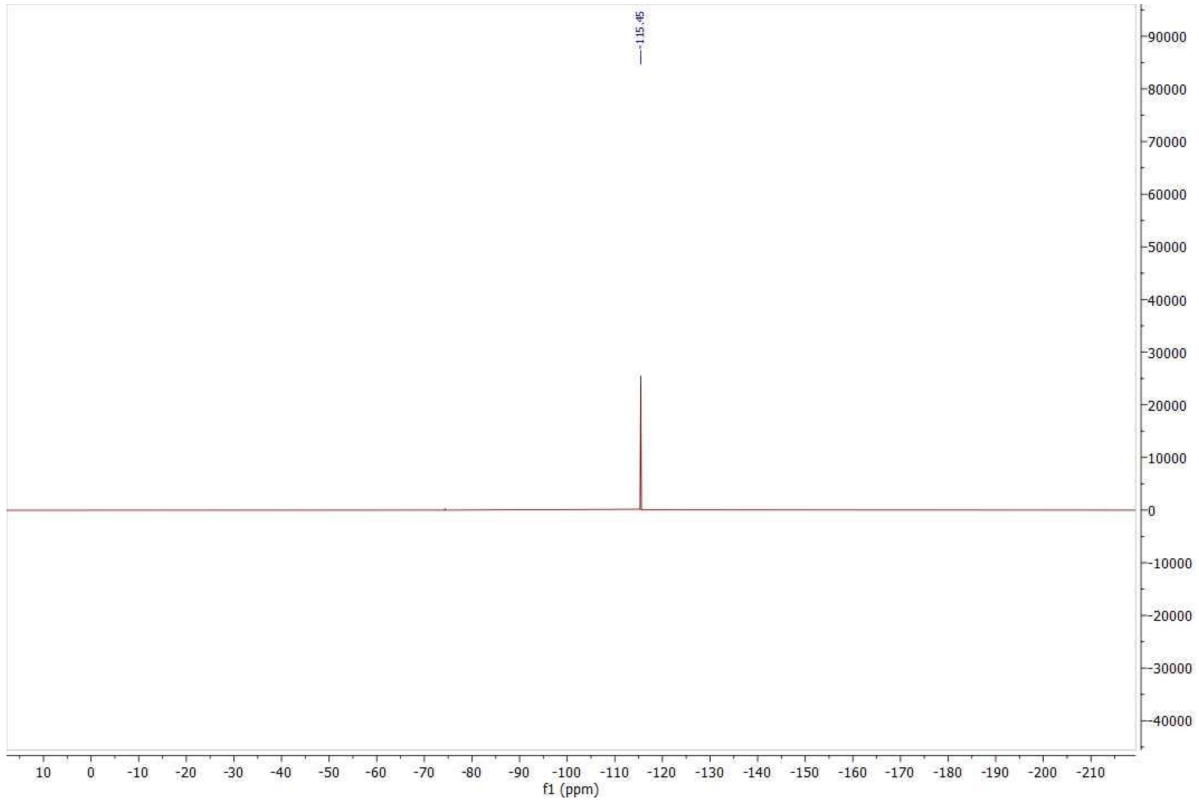

-115.45
90000
80000
70000
60000
50000
40000
30000
20000
10000
0
-10000
-20000
-30000
-40000
10 0 -10 -20 -30 -40 -50 -60 -70 -80 -90 -100 -110 -120 -130 -140 -150 -160 -170 -180 -190 -200 -210
f1 (ppm)

^{1}H-NMR spectrum of **nc-3a**

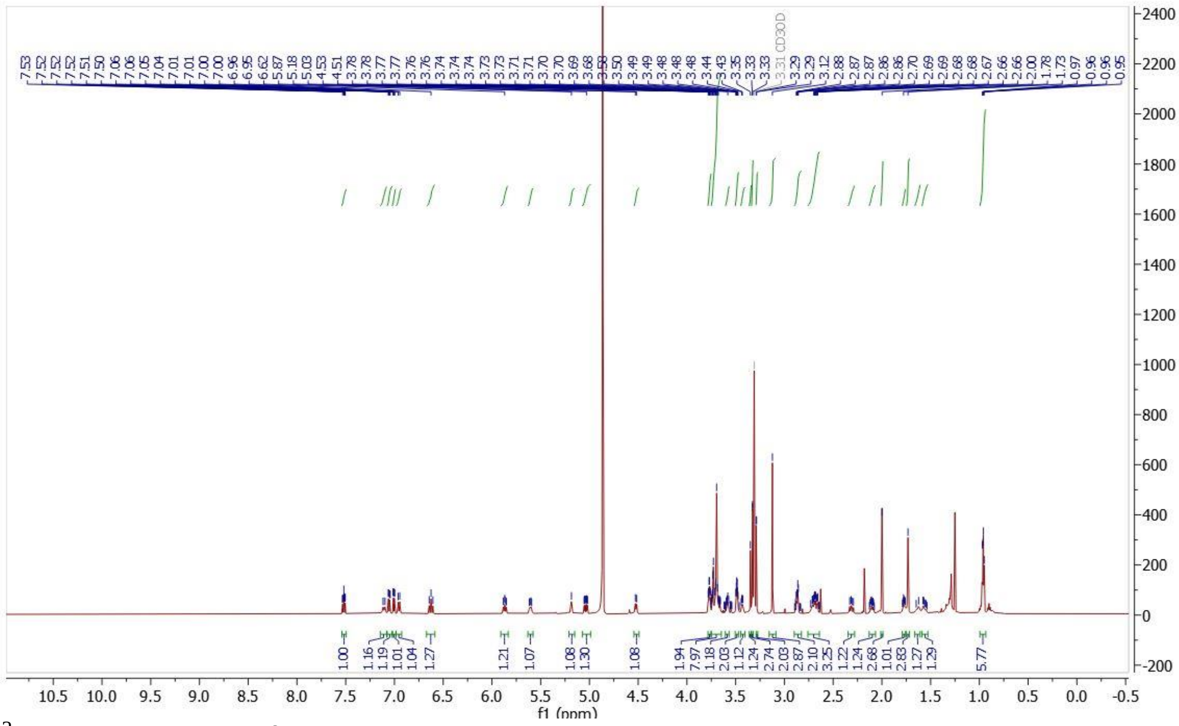

^{13}C-NMR spectrum of **nc-3a**

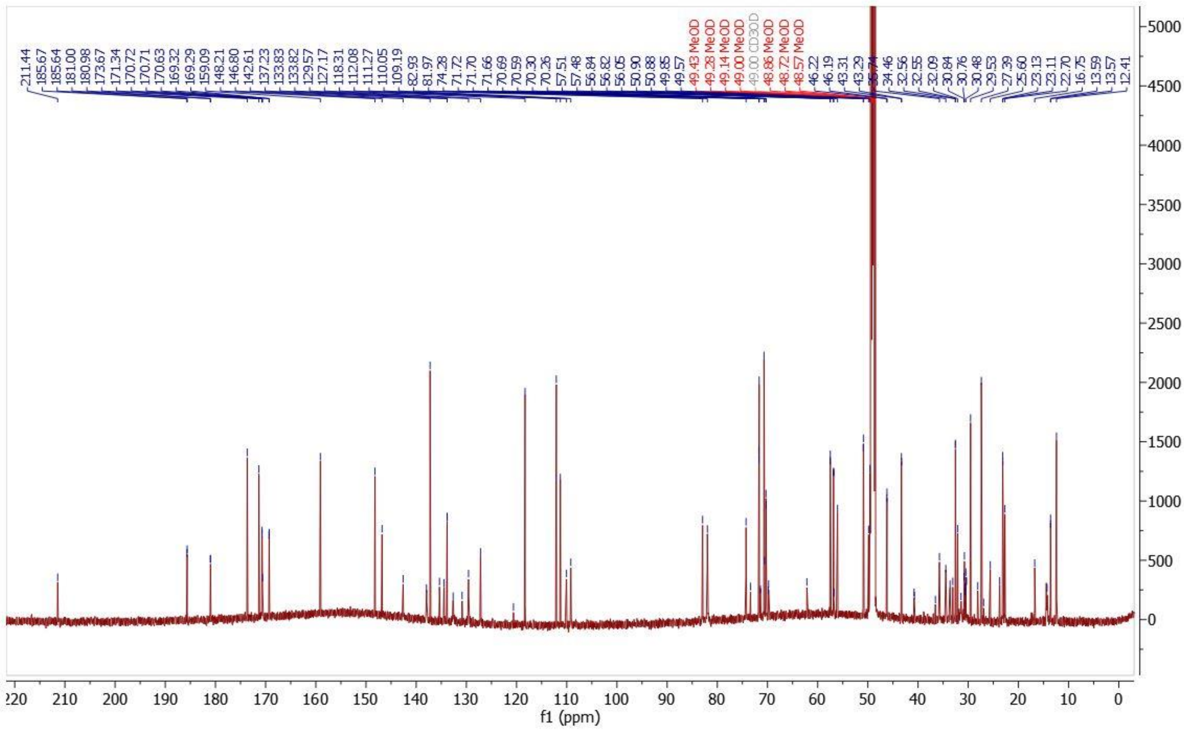

2 Supplementary Figures

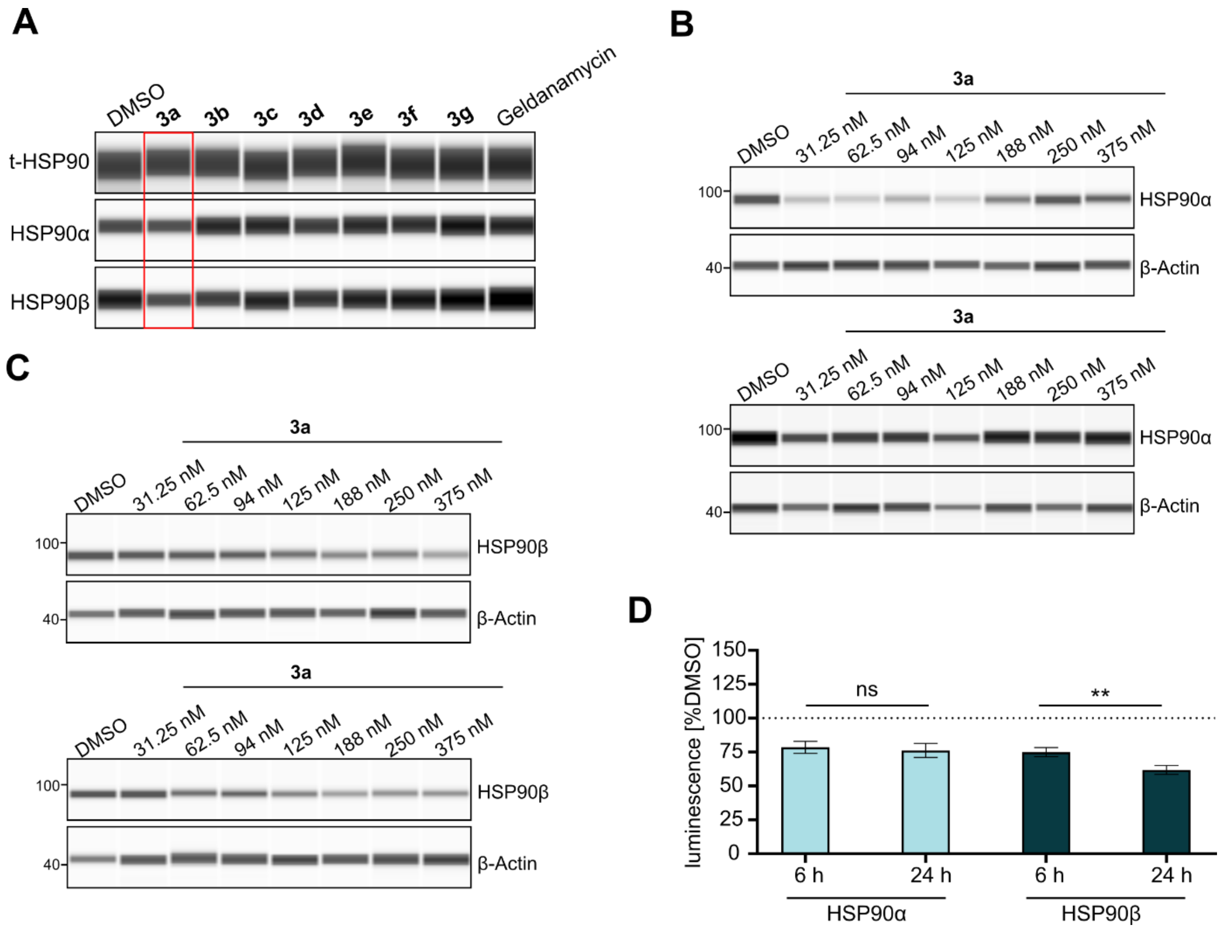

Figure S1: (A) JESS run to select the most active HSP90-degrader at 1 µM. **(B-C)** JESS run replicates with different concentrations of **3a** (31.25 nM - 375 nM) showing the degradation of HSP90α **(B)** and HSP90β **(C)**. **(D)** NanoGlo lytic assay using K562 HiBiT cells treated with **3a** (200 nM) for 6 h and for 24 h.

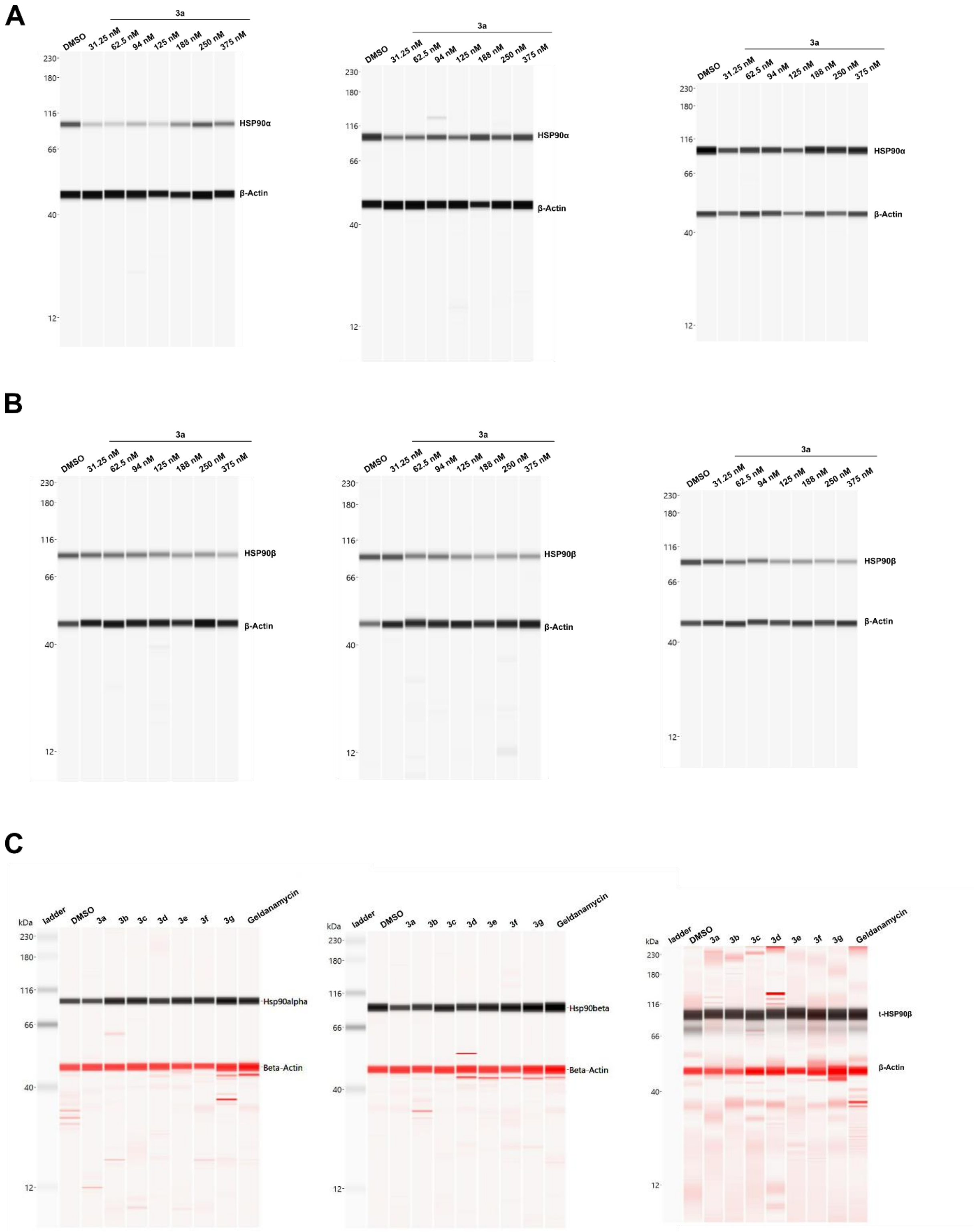

Figure S2: Full Blots of replicates with different concentrations of **3a** (31.25 nM - 375 nM) of HSP90α **(A)** and HSP90β **(B)**. **(C)** Full Blots of the selection of the most active HSP90-degrader at 1 µM.
